# Characterizing rhizosphere microbiota of peanut (*Arachis hypogaea* L.) from pre-sowing to post-harvest of crop under field conditions

**DOI:** 10.1038/s41598-021-97071-3

**Published:** 2021-08-31

**Authors:** Ankit T. Hinsu, Ketankumar J. Panchal, Ramesh J. Pandit, Prakash G. Koringa, Ramesh K. Kothari

**Affiliations:** 1 Department of Biosciences, Saurashtra University, Rajkot, 360005 India; 2 Department of Animal Biotechnology, College of Veterinary Sciences & A.H., Anand Agricultural University, Anand, 388001 India

**Keywords:** Metagenomics, Microbial ecology, Microbiome, Soil microbiology

## Abstract

The rhizosphere, a narrow zone of soil near plant roots, is a hot spot for microbial activity. Rhizosphere microbiota directly or indirectly benefit plants by supplementing nutrients, producing beneficial chemicals, or suppressing pathogens. Plants attract and modulate bacteria within the rhizosphere by releasing exudates. Plants also tend to select the rhizosphere microbiota based on their needs; a phenomenon termed as “rhizosphere effect”. In this study, we characterized the rhizosphere microbiota of peanut plants across the crop development cycle from pre-sowing of seeds to post-harvest of crop under field conditions. The rhizosphere and bulk soil samples from different crop developmental stages were also compared. The composition of bulk soil microbiota resembled microbiota of pre-sowing and post-harvest soil and was markedly different from rhizosphere soil samples. Rhizosphere samples were enriched with multiple organisms mostly from the Proteobacteria, Firmicutes and Bacteroidota phyla. Differences in diversity were observed among the rhizosphere samples but not in bulk soil across different crop development stages. *Pseudomonas_M indica* was highly enriched during the germination of seeds. Furthermore, Plant Growth Promoting (PGP) bacteria like *Bacillus* were enriched during the middle stages of crop development but there was a decline in PGP organisms in the matured crop stage. We also observed a significant association of pH and Electrical Conductivity (EC) with the profiles of microbial community. Overall, this study portrayed the changes in rhizosphere microbiota of peanut during different developmental stages of crop and may help to design stage specific bio-strategies such as bio-fertilizer to improve crop yield.

## Introduction

Leonardo da Vinci said that “We know more about the movement of celestial bodies than about the soil underfoot”. This remains true in the twenty-first century. Most of these mysteries can be attributed to the microscopic lives in the soil. Microorganisms, especially bacteria, are abundant in soil with concentrations as high as 10^11^ cells per gram of soil^1,2^. Soil microbes are not only involved in major biogeochemical processes but also help plants in various essential functions including nutrient acquisition, stress tolerance and pathogen resistance^3^. Plant-associated microbes can be differentiated into different types based on their locations and vicinity to plants^4^. The rhizosphere is a zone near the vicinity of roots and is a hotspot for microbial activities^3^. The rhizosphere hosts a dynamic microbial community involved in microbe-microbe and microbe-plant communications mediated by plant exudates^5^. Rhizosphere microbes respond to plant exudates and help plants via various plant growth promoting impacts including defence against pathogens^1^. This makes understanding the rhizosphere microbiome an important part of sustainable agriculture. However, the rhizosphere microbiome is very dynamic and changes in response to various internal and external factors making it an exceptionally complex ecosystem^4,6^.

Sequence driven metagenomics has emerged as the approach of choice to study microbiota from various habitats including soil. Many of the previous studies have used 16S rRNA gene based community-profiling to study rhizosphere microbiota from diverse plant and crops like Arabidopsis, rice, millet, soybean, corn, barley, wheat, tomato, grapes and many others^3,4,7–12^. While many studies have looked at the effects of soil type, geographic location, crop genotypes and several other factors, very few have looked at the changes in microbiome across the crop developmental cycle^2–4,6–8,13–26^. Also, very few studies have been conducted on the peanut rhizosphere^27,28^. Peanut, also known as groundnut in some parts of world, is a leguminous crop cultivated in more than 100 countries worldwide^29^. The plant is peculiar in the respect that while the flower fertilises above ground, the pod matures in the soil. Furthermore, peanut is a rotation crop which benefits the succeeding crop by enriching soil fertility through atmospheric nitrogen fixation^30^.

In this study, we used a 16S rRNA gene amplicon-based approach to study the microbiota of the rhizosphere associated with peanut and the nearby bulk soil. The study looks at the horizontal profile of microbiota throughout the crop developmental cycle starting from pre-sowing of seeds to post-harvest of the crops. The samples included seedling (S), PreNodulating (PN), Nodulating (N), Flowering (F) and Matured (M) stages of crop development. Although, the microbiota structure changes with the minutest of changes in several factors, we believe that looking at this kind of horizontal structural changes can form a foundation for a sustainable agriculture approach.

## Results

In this study, we looked at the structural changes in the community of soil organisms (rhizosphere and nearby bulk soil) during the entire cycle of peanut development starting from pre-sowing of seeds to post-harvest of crop (Figure S1, Table 1). We also checked physical properties and concentrations of important macronutrients and micronutrients in the soil. We started by looking at the changes in the soil properties. Among the four groups of soil samples (PreSowing (PS), PostHarvest (PH), Bulk and Rhizosphere), significant differences (Kruskal–Wallis p-value < 0.05) were observed in pH, Electrical Conductivity (EC), and concentrations of all macro and micro nutrients except phosphate and %Organic Carbon (OC) (Table S1). Furthermore, significant differences (paired Wilcoxon-test p-value < 0.05) were observed only in pH and EC between paired samples of Bulk and Rhizosphere soil (Table S1). Significant differences (Kruskal–Wallis p-value < 0.05) were also observed in pH, EC, OC and the concentrations of Sulphur (S), Manganese (Mn), and Iron (Fe) among different developmental stages in rhizosphere soil samples (Table S1).

**Table 1:** Details about the sample collection.

Sr. No	Date	Plant age from sowing (days)	Sample codes	Crop development stage	Remarks/details
1	07-05-2017	Not applicable	PS(1–5)	PreSowing	15 days prior to sowing and 5 days after addition of DAP
2	21-05-2017	5	R1(1–5), B1(1–5)	Seedling	5 days after sowing of seeds, irrigated with water from borewell during sowing
3	02-07-2017	47	R2(1–5), B2(1–5)	PreNodulating	No rain during the period and hence, no change in the crop
4	23-07-2017	68	R3(1–5), B3(1–5)	PreNodulating	Presence of nodules, last rainfall before 3 days
5	06-08-2017	82	R4(1–5), B4(1–5)	Nodulating	Rainfall during the period, presence of 2–3 groundnut pods
6	20-08-2017	96	R5(1–5), B5(1–5)	Nodulating	5–10 flowers per plant, rainfall during the period, presence of 5–10 groundnut pods
7	03-09-2017	110	R6(1–5), B6(1–5)	Flowering	10–15 flowers per plant, presence of 10–15 groundnut pods
8	17-09-2017	124	R7(1–5), B7(1–5)	Flowering	Matured crop with 15–25 groundnut pods
9	08-10-2017	145	R8(1–5), B8(1–5)	Matured	Crop fully matured with 25–40 groundnut pods. Harvest of crop from 09-10-2017
10	18-10-2017	155	PH(1–5)	PostHarvest	Post-harvest sample

### Diversity from sequencing data

The V3-V4 region of 16S rRNA gene was amplified and sequenced on Illumina MiSeq using 250 × 2 paired-end chemistry. Around 6.1 million sequence reads from all 90 samples with an average count of 67,766 reads per sample was generated. The data was processed using DADA2 package in R and the default pipeline inferred 31,742 Amplicon Sequence Variants (ASVs) from 3.8 million reads (63.12%). ASVs with less than 6 supporting reads and samples with less than 10,000 processed reads were filtered out, leaving behind 21,764 ASVs from 88 samples, which were analysed further. Five databases used for taxonomy assignment showed different levels of annotation (Figure S2) however, assignment from GTDB r89 was considered for further analysis (more details in discussion section). All these ASVs were taxonomically classified as Bacteria and assigned uniquely to 38 phyla, 80 classes, 195 orders, 313 families, 664 genera and 90 species.

**Figure 1:**
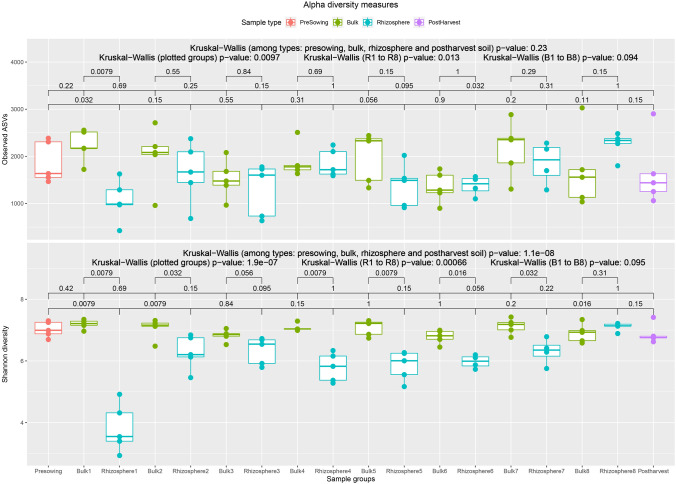
Alpha diversity measures Observed ASVs (top), and Shannon Index (bottom) plotted against sample groups. p-value from Wilcoxon-test between groups are given by bracket pointing to groups. p-value from Kruskal–Wallis test among four types/groups of samples is mentioned on the top. Second line mentions p-value from Kruskal–Wallis test of all groups (as plotted), all rhizosphere sample groups/collections and bulk soil sample groups/collections.

The number of ASVs ranged from 425 (in R1 group) to 3,030 (in B8 group) (Fig. 1). No significant differences were observed among the four groups of samples (Kruskal–Wallis p-value = 0.23). Significant differences were observed among all the collections in rhizosphere samples (Kruskal–Wallis p-value = 0.013) but not in samples from bulk soil (Kruskal–Wallis p-value = 0.094). Shannon diversity index, which accounts for the relative proportion of each ASV, was observed in range of 2.94 (in R1 group) to 7.43 (in B7 group) (Fig. 1). Unlike Observed ASVs, Shannon index differed significantly among the four sample types (Kruskal–Wallis p-value < 0.001) and among all collections of rhizosphere samples (Kruskal–Wallis p-value = 0.0007) but not among all collections of bulk samples (Kruskal–Wallis p-value = 0.095). Furthermore, no significant differences were observed among samples of PS, PH and bulk soil. Significantly higher Shannon diversity (Kruskal–Wallis p-value < 0.05, except Collection-3 where p-value = 0.056) was observed in bulk samples compared to rhizosphere samples of same collection except from samples of Collection-8.

### Taxonomic content of microbial communities

**Figure 2:**
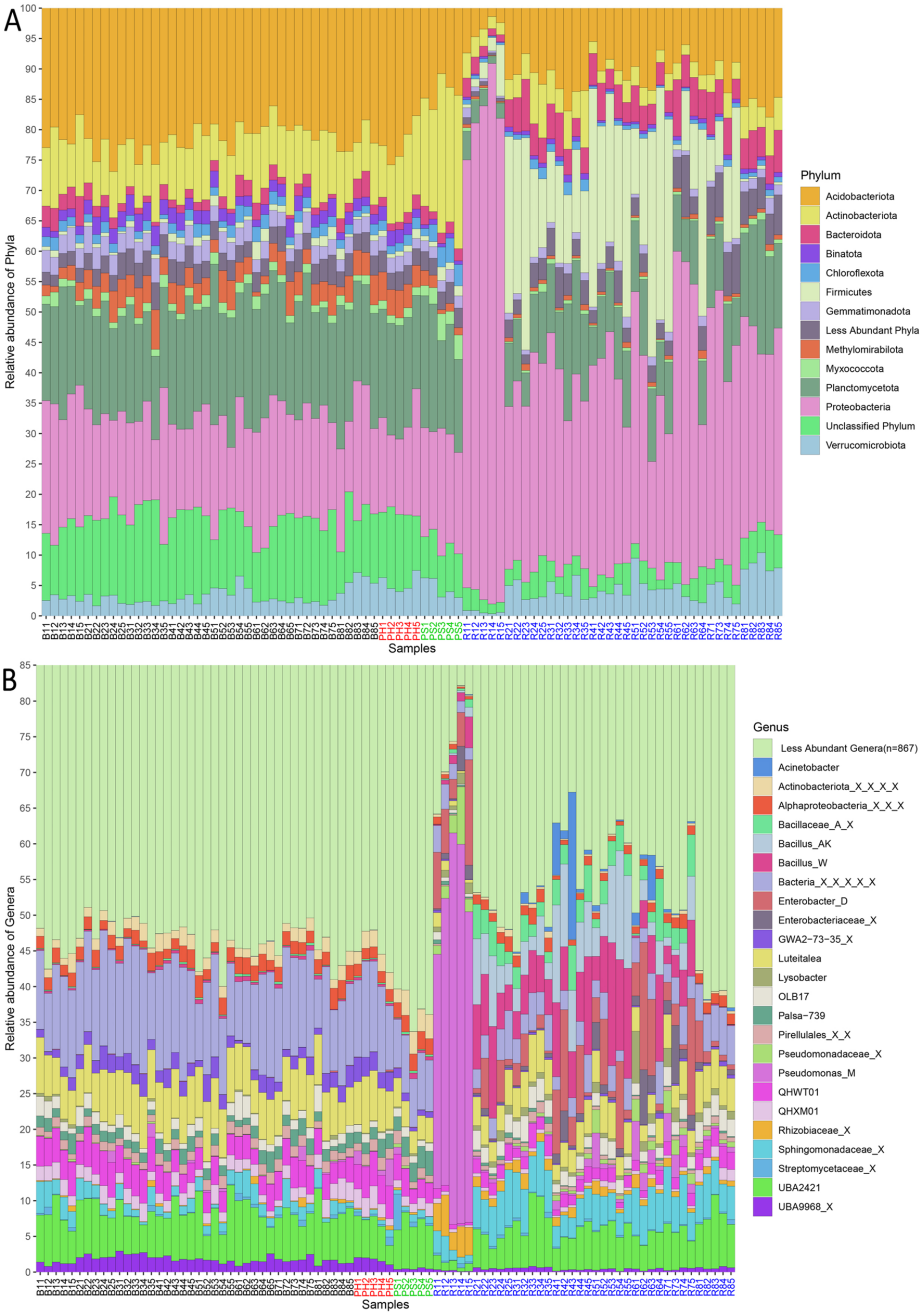
Taxonomic distribution at (**A**) phylum level and (**B**) Genus level. Only the top taxa are plotted for both levels. Sample names are coloured by type of sample.

All the ASVs were classified as Bacteria and were distributed in 38 phyla and 664 genera. Common soil phyla like Acidobacteriota, Actinobacteriota, Planctomycetota and Proteobacteria were abundant (median relative abundance > 5%) (Fig. 2A). Additionally, Firmicutes and Bacteroidota phyla had higher abundance in rhizosphere samples. At the genus level, *Enterobacter_D, Bacillus_W, Luteitalea, UBA2421, QHWT01, Pseudomonas_M*, unknown member from Sphingomonadaceae family and some unknown bacteria were the most abundant (mean relative abundance > 2%) (Fig. 2B). Clear differences were observed between rhizosphere samples and other samples. R1 group samples were overly abundant with Proteobacteria phyla (~ 70%—85%) and genera like *Pseudomonas_M, Enterobacter_D* and unclassified genera of *Rhizobiaceae* and *Pseudomonadaceae* family, which explains the lower diversity in the group. Very few highly abundant ASVs were assigned up to species level. However, many of these ASVs were dominant in rhizosphere samples (Figure S3). The most abundant species were *Pseudomonas_M indica, Bacillus_AK korlensis* and *Enterobacter_D cloacae*, all of which belonged to most abundant genera in rhizosphere samples. Other species observed abundantly in rhizosphere samples include *Microvirga vignae, Inquilinus limosus, Flavobacterium anhuiense, Stenotrophomonas maltophilia_J, Chryseobacterium geocarposphaerae, Sphingopyxis macrogoltabida, Exiguobacterium profundum,* and *Cupriavidus alkaliphilus*.

### Differences between four groups of samples

**Figure 3:**
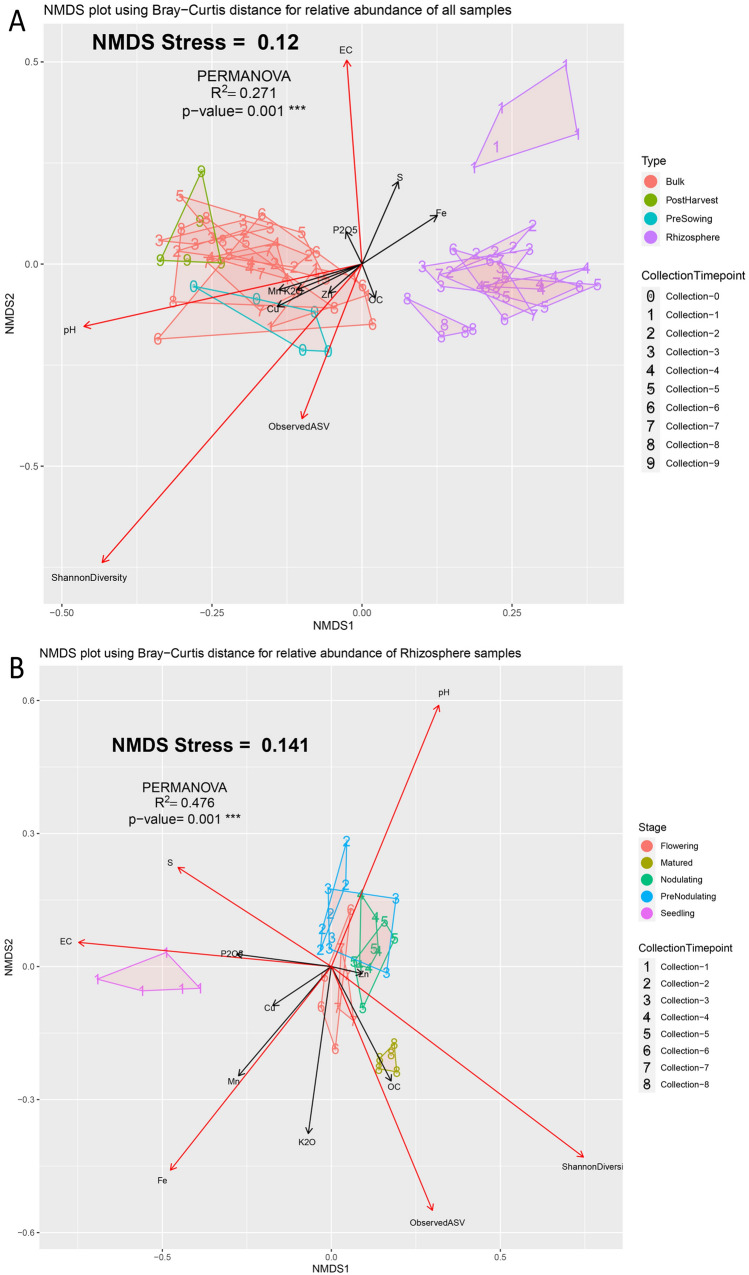
NMDS plot of Bray–Curtis distance calculated from (**A**) all samples and (**B**) Rhizosphere samples only. Collection number of each sample is used as shape to denote the sample. Environment fit vectors representing physical parameters and nutrient concentrations are shown as arrows. Vectors with significant associations are shown in red coloured arrow.

Non-Metric Dimensional Scaling (NMDS) of Bray–Curtis distance was performed to check the patterns of separation between microbial communities across groups. Samples from bulk soil, PS and PH groups formed a separate cluster from rhizosphere soil samples (Fig. 3A). Separate clusters were also observed for R1 and R8. Permutational multivariate analysis of variance (PERMANOVA) corroborates that four groups of samples showed significant differences (R^2^ = 0.271, p-value < 0.001). Furthermore, this differentiation also associated significantly (p-value < 0.05) with the distribution of pH, EC, Shannon diversity and Observed ASV. These differences were also evident from the observation that almost half of the observed genera (420 of 891, including the most abundant genera) differed significantly (Kruskal–Wallis BH p-value < 0.05) among the four types of samples (Table S2, Figure S4). However, limited significant changes were observed among bulk, PS and PH samples (Table S3).

The differences between types of samples were also observed in terms of significant (adjusted p-value < 0.01) log2FoldChange (lFC) of ASVs between pairs of groups as checked using DESeq2 (Figure S5). There were more significant differences in lFC between ASVs in rhizosphere samples compared to bulk (n = 827), PH (n = 254) and PS (n = 110) with lFC as high as 30. Significantly differing ASVs from phyla Firmicutes, Firmicutes_A, Firmicutes_I and Patescibacteria were more enriched in rhizosphere samples. Specifically, genera like *Pseudosphingobacterium, Chryseobacterium, Neorhizobium, Haloferula, Verrucomicrobium, Sphingosinicella*, and members of *Bacillaceae_A, Pseudomonadaceae, Enterobacteriaceae, Alteromonadaceae, Rhizobiaceae* and *Burkholderiaceae* family were present with lFC more than 20 in rhizosphere samples. While very few ASVs differed significantly between PS and bulk (n = 3); PS and PH (n = 13); bulk and PH (n = 15).

### Differences between pairwise bulk and rhizosphere samples

The intriguing differences between bulk and rhizosphere samples compelled us to look at the differences between these groups for each collection. In total, 446 genera out of 891 differed significantly (Wilcoxon test p-value < 0.05) between bulk and rhizosphere samples (Table S3). Furthermore, 41, 97, 126, 93, 86, 51, 47 and 56 ASVs were observed to have significant (adjusted p-value < 0.01) lFC between bulk and rhizosphere samples from Collection 1 to 8, respectively (Figure S6). ASVs belonging to Proteobacteria, especially multiple ASVs of *Enterobacter_D, Pseudoxanthomonas_A, Cupriavidus* and some unknown members of *Pseudomonadaceae* and *Enterobacteriaceae* family, were more abundant in rhizosphere (with lFC > 10) across all collections. Additionally, ASVs from Firmicutes phylum (*Bacillus_AK* and some unknown *Bacillaceae_A* family) were also abundant in R1 to R7, but were significantly enriched (lFC > 10, p-value < 0.01) in B8. Some changes were also observed confirming the developmental stage-like patterns. For example, members of *Rhizobia* (*Neorhizobium* and multiple ASVs assigned to unknown *Rhizobiaceae* family), are nitrogen fixers associated with root nodules of legumes, and were observed to be significantly enriched with a lFC as high as 40 in Collection 1 to 5, corresponding to the early S, PN and N developmental stages, but were not significantly different thereafter.

### Changes among rhizosphere samples associated with crop development stages

Lastly, we looked at the changes in the rhizosphere samples with respect to crop developmental stages. NMDS ordination on Bray–Curtis distance revealed a separate and distinct cluster for R1 (S stage) and R8 (M stage) (Fig. 3B). Except for 2 samples from R3, all other samples from R2 to R7 were grouped according to developmental stage (PN, N and F). However, all these collections formed clusters quite close to each other. The difference among developmental stages were further confirmed through PERMANOVA where significant difference was observed (R^2^ = 0.476, p-value < 0.001). A pairwise-adonis between all pairs of collections were significant except for R4–R5 (p-value = 0.215), R6–R7 (p-value = 0.557) and R2–R3 (p-value = 0.057), all of which are the collections from the same developmental stage (Table S4). An environmental fit of all variables also revealed a significant association of pH, EC, concentrations of S and Fe, Observed ASVs and Shannon diversity. Further, increase in OC, Shannon diversity and Observed ASVs with crop development and decrease in EC, phosphate, S concentration with crop development was observed from ordisurf plots (Figure S7).

At the phylum level, 28 out of 30 phyla (with mean relative abundance > 0.0001) were significantly different (Kruskal–Wallis, BH p-value < 0.05) among developmental stages (Table S5, Figure S8). We believed that this could be happening because of the hugely different and less diverse samples in R1 group. Removing the R1 group samples decreased the number of significantly differing phyla (Kruskal–Wallis, BH p-value < 0.05) to 22 out of 30. Similarly, at genus level, 306 out of 611 and 236 out of 611 genera (mean relative abundance > 0.00001) differed significantly (Kruskal–Wallis, BH p-value < 0.05) among developmental stages with and without R1 group, respectively (Table S6, Figure S9). Many of the higher abundance genera like *Acinetobacter, Bacillus_AK, Bacillus_W*, unknown genera from Bacillales order and *Bacillaceae* family seemed to increase with crop development stage till N stage and then decrease, forming a bell-like shape. While other abundant genera like *Chthoniobacter, QHWT01, UBA2421* and unknown genera from *Burkholderiaceae* family seemed to increase with crop developmental stages throughout the life cycle. However, very few ASVs (less than 25) were observed to differ significantly (adjusted p-value < 0.01) as per DESeq2 (Figure S10). Around 53 ASVs differed significantly (p-value < 0.01) between PS and S stage, most of which were PGP bacteria from the Proteobacteria. Further, 23, 22 and 3 ASVs differed significantly between S, PN, N and F stage, respectively. Nineteen ASVs, most of which were from Bacillales order, reduced (lFC > 5) significantly (p-value < 0.01) in M stage compared to F stage. Similarly, 139 ASVs, most of which reduced with lFC > 5, differed significantly between M and PH stage.

**Figure 4:**
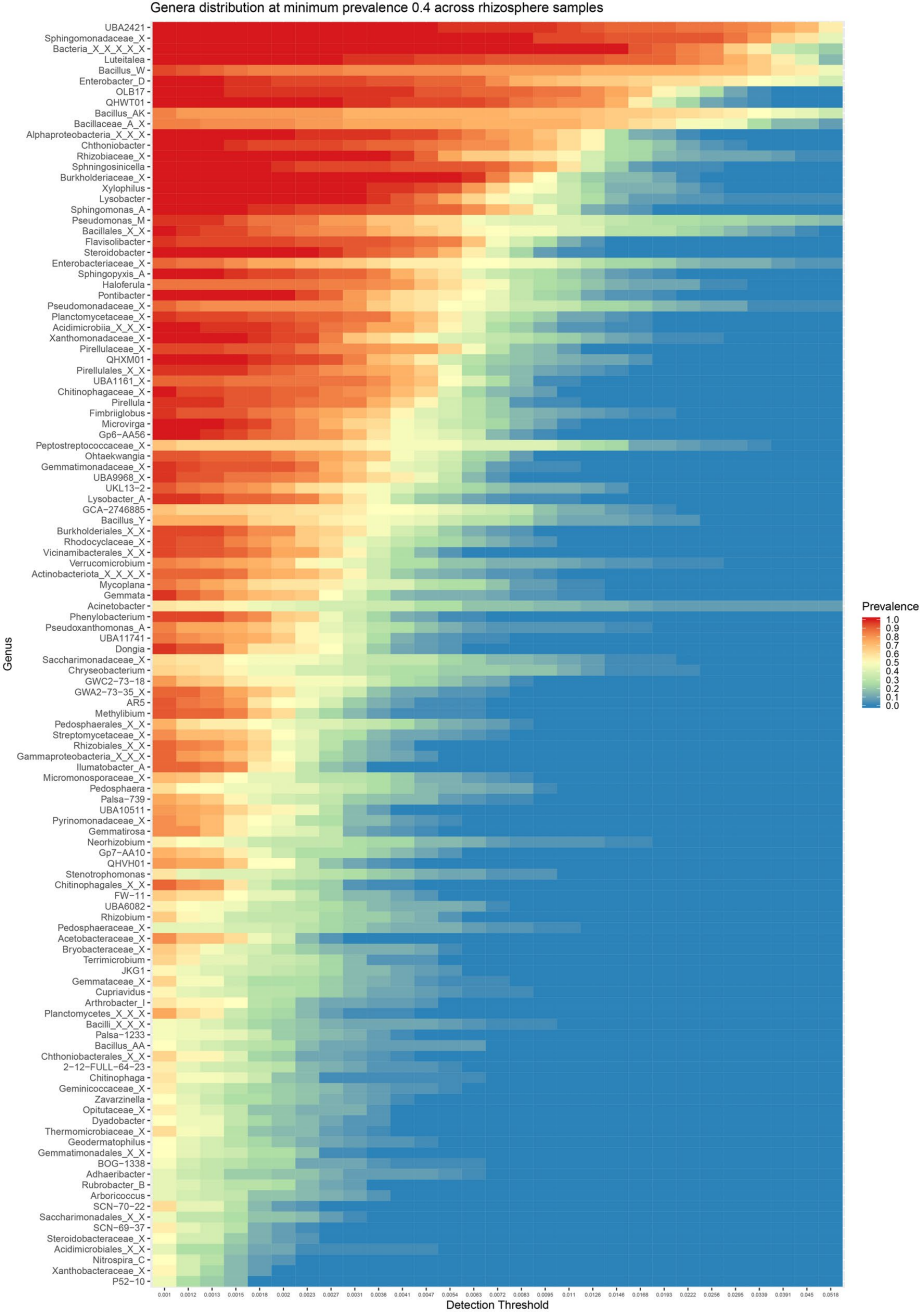
Plot representing core microbiome from rhizosphere samples. The plot compares prevalence of genus in samples across varying levels of abundance. Only the genera with minimum prevalence of 0.4 at 0.001 abundance are plotted.

**Figure 5:**
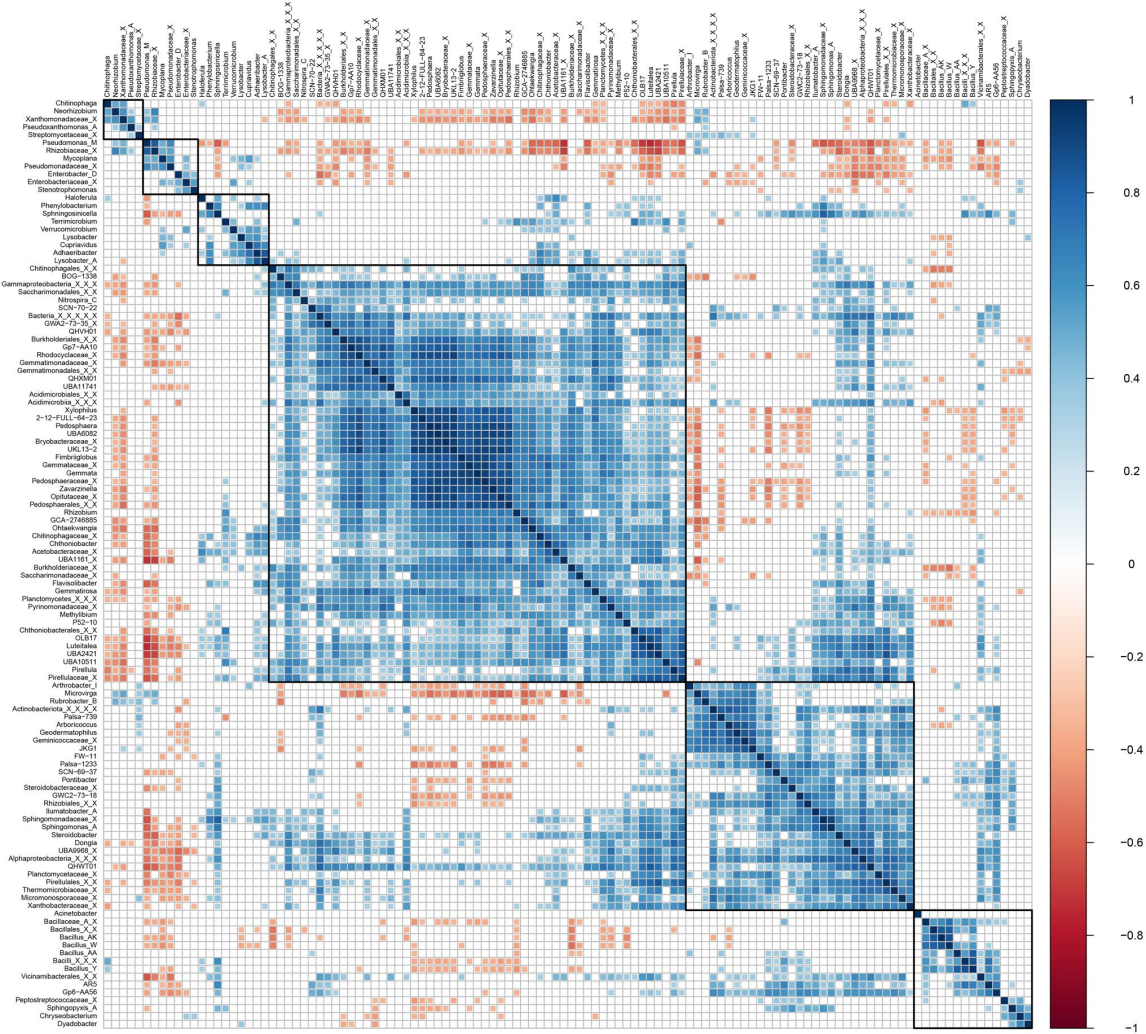
Correlation plot among genera from core microbiome. Only the significant (p-value < 0.05) correlations are plotted.

There were 118 genera identified as part of core microbiome of rhizosphere samples with minimum abundance 0.1% across 40% samples (Fig. 4). These taxa were further correlated among each other to see the co-occurrence pattern. Six different clusters of genera could be made out from the significantly (p-value < 0.05) correlating genera (Fig. 5). Two clusters were observed to be showing a negative correlation with all the other genera. These contained genera like *Neorhizobium, Pseudomonas_M, Xanthomonadaceae_X, Rhizobiaceae_X, Pseudomonadaceae_X, Enterobacter_D* and *Enterobacteriaceae_X*, many of which were overly abundant in S stage. A separate cluster could also be made out containing mostly Bacilli class members like *Bacillus_AK, Bacillus_W, Bacillus_AA, Bacillus_Y* and unknown members of *Bacillaceae_A* family, Bacillales order and Bacilli class. Many of these genera, especially higher abundant ones showed negative correlation with all other genera probably explaining their increase during PN, N and F stages. Apart from these, two other clusters were observed which included majority of the genera but showed no specific pattern of correlation.

## Discussion

The 16S rRNA gene sequencing-based approach is commonly used to characterise various microbiomes including soil and rhizosphere under various conditions and stresses. In this study, we analysed the bulk soil and rhizosphere microbiota using Illumina based 16S rRNA gene sequencing. To provide more resolution to the analysis, we opted for the DADA2 approach for data analysis^31^. DADA2 is a denoising algorithm developed specifically for Illumina data and infers Amplicon Sequence Variants (ASVs) with differences of a single nucleotide and hence, retains strain-level information. In our study, we could observe several ASVs distinguished to species level (Figure S3) including some ASVs with higher abundances. However, the power of analysis also depends on the choice of database for taxonomy assignment. We used SILVA v132, RDP trainset 16, RDP trainset 16 + RefSeq (sequences from NCBI accessed on 14-05-2018) and GTDB versions 86 and 89 for taxonomy assignment and decided to use GTDB v89 for the entire analysis. Our decision to use GTDB was based on the observation that higher number of variants were annotated at genus level in GTDB (Figure S2) and also because of the taxonomy lineage assignment method in GTDB^32,33^. GTDB is a curated database with comprehensive genome-based taxonomy based on monophyly and relative evolutionary divergence of taxa, which is an added advantage while annotating ASVs. The reclassifications by GTDB works well by distributing/reclassifying popular genera into several novel ones^32^. For example, *Pseudomonas* genus was divided into 15 genera, 1 retaining the name *Pseudomonas* consisting of *P. aeruginosa* while, 14 other labelled with a letter from A to O (*Pseudomonas_A* to *Pseudomonas_O*)^34^. This gives a higher resolution to the observed organisms in the study. For example, we observed higher abundances of *Pseudomonas_M*, which is represented by a single species *P. indica*, and not any other *Pseudomonas* genera suggesting the differences in levels among all *Pseudomonas* genera. Similar observations were also made with *Bacillus* genus where ASVs classified as *Bacillus_AK, Bacillus_W* and some unclassified *Bacillaceae_A* (which includes *Bacillus_AK, Bacillus_W* and other genera) were the most abundant.

Previous studies have focussed on the microbiome from different compartments of soil^4^. In this study, we compared the rhizosphere microbiota with nearby bulk soil microbiota. In our study, we observed significantly higher Shannon diversity in bulk soil samples compared to rhizosphere, except for samples from M stage. This type of pattern is reported in several earlier studies^3,17,23,24^, while many other studies have reported mixed results^7,14^. While the rhizosphere effect (i.e., compositional and functional difference of microbiome between rhizosphere and bulk soil) attracts beneficial microbes and increases the microbial activity in the rhizosphere, the bacterial diversity remains lower compared to bulk soil apparently^3^. We also observed the significant differences in rhizosphere microbiota diversity across crop cycle but not in bulk soil which suggests that the development stage of crop is one of the major drivers of the rhizosphere effect. Although samples of PS and PH were collected from barren farm, we were expecting more similarity between PH and rhizosphere samples. However, PH samples were very similar to bulk samples. This shows that bacteria community changed from rhizosphere-like to bulk-like within span of 10 days in the absence of plants.

In accordance with previous studies, we observed higher abundances of Proteobacteria, Acidobacteria (named as Acidobacteriota in GTDB), Actinobacteria (named as Actinobacteriota in GTDB) and Planctomycetes (named as Planctomycetota in GTDB) in all samples^3,22^. Additionally, higher abundance of Firmicutes, Bacteroidetes (named as Bacteroidota in GTDB) and Verrucomicrobia (named as Verrucomicrobiota in GTDB) were also observed in the rhizosphere samples. A higher abundance of Firmicutes has been reported in the rhizosphere in a few earlier studies^16,19,25,35^ including a study on peanut microbiome^27^, however this was not observed in other studies on the peanut rhizosphere^28,36^. The majority of DESeq2 based enriched ASVs in rhizosphere samples also belonged to Proteobacteria, Firmicutes and Bacteroidota phyla while ASVs enriched in bulk soil belonged to Acidobacteriota, Actinobacteriota and Planctomycetota (Figure S5). Similar findings have been reported in earlier studies where plant growth promoting bacteria from Proteobacteria and Firmicutes phyla were enriched in rhizosphere^3,26,37^. Overall, *Pseudomonas_M, Acinetobacter, Enterobacter, Bacillus_W, Bacillus_AK, UBA2421* were the most abundant genera in rhizosphere. All of these are reported to show PGP activities. For example, *Pseudomonas* and *Acinetobacter* are well studied for their biofilm properties^38^, and siderophore production^39^, *Enterobacter cloacae* (most of *Enterobacter* in present study were annotated as *Enterobacter cloacae* in rhizosphere) are associated with heavy metal and salinity stress removal^40,41^, and *Bacillus* are reported to possess several beneficial activities including solubilization of phosphate, nitrogen fixation and siderophore production^42^. *UBA2421*, a genus observed with > 3% abundance in all samples, is a yet uncharacterised organism belonging to Planctomycetota phylum. To date, this genera has only been described in databases as a Metagenome assembled genome (MAG) with no known cultured isolates^43^. Several other genera described exclusively by MAGs are expected depending on whether the selected database includes MAGs. This highlights the fact that there are several higher abundant organisms whose roles are yet to be evaluated in environmental context.

Earlier studies have hypothesized and reported that plants initially perform a general recruitment near the root vicinity and then allows microbes with species-specific signatures to colonise within^9^. Our study shows similar results wherein Proteobacteria and specifically *Pseudomonas_M indica* was observed in higher abundance in R1 group and then decreased in abundance. Only two ASVs (ASV1 and ASV31726) were annotated as *Pseudomonas_M* and present in sufficient abundance for inclusion in the study (mean rel. abundance > 0.0001). These ASVs were observed in all samples, including PS samples, at lower abundances. This implies that the organism was already part of soil but enriched during the germination of seeds, similar to what have been observed in previous studies^4^. It could be speculated that *Pseudomonas_M* was part of the bulk and PS soil microbiota, recruited on seed germination, colonised the roots, and then abundance reduced in rhizosphere as the plant matured. It is possible that stable higher abundance of *Pseudomonas_M* could be present in rhizoplane and endosphere microbiota. Many other studies have reported N-fixing and other PGP properties of *Pseudomonas* including *P. indica*^44^ and reports are available for their use in fields as growth promoter. *Pseudomonas* are also shown to produce biofilm, which is crucial for colonization of bacteria on plant root surface^45^. *P. indica* was first isolated from oilfields in Gujarat, India while the sample source for this study is also from Gujarat, India^46^. Furthermore, earlier study have also reported higher abundance of Proteobacteria (as high as 50–55%) in the rhizosphere up to 13 days after germination and a comparatively higher proportion in the rhizoplane^4^. Similar to our results, the previous study also highlighted that plants recruit the microbiome from soil rapidly, stabilising the communities within two weeks. Since this study did not involve sterilization of seeds, introduction of microbiota from seed is also a possibility.

Many recent studies have reported on the transmission of microbiota through seeds^47,48^ . These microbes inhabit the rhizosphere after germination of seeds through cotyledon defoliation and root development. These microbes are also linked to seedling survival during early stages^49^. It has also been postulated that microbiota of rhizosphere and several other endophytic microbes colonise the seed and are transmitted. PGP organisms like *Pseudomonas*, *Bacillus*, *Acinetobacter*, *Pantoea*, etc. are commonly identified as seed endophytes with the potential to attract more beneficial microbes and suppress pathogens^49,50^. In the current study, there were fewer genera found exclusively in R1 (n = 52) compared to PS (n = 143) (data not shown). Since the farm has sown the same variety/genotype of seeds for the previous three years, it can be speculated that the diversity was already introduced in the soil, explaining the lower number of genera observed exclusively in R1 samples. However, these genera could also be introduced to the soil and have their growth elevated by irrigation. The farm also followed the practice of using water from wells during early sowing followed by a dependence on rain for water thereafter. This led to an extensive dry period without water to plants of approximately forty days during the sampling cycle. However, the samples collected during this period (R2) and after the first rainfall (R3) showed similar microbiota profile (pairwise Adonis p-value = 0.057). Moisture content is one of the important abiotic factors influencing microbiota structure. Therefore, we tried to minimise possible biases arising due to favourable environment provided by moisture content by collecting samples a minimum of three days after the last rainfall^51^.

It was postulated way back in 1995^52^ that plants release exudates to modulate surrounding rhizobacterial communities. It was also postulated that secretion of exudates changes with change in plant development stage^15^. The plants have co-evolved with the surrounding microbes in order to utilize their potential benefits. The plants regulate the microbes through exudate secretion and selects for the best organisms which can help in plant growth promotion through different ways^53^. This makes the rhizosphere microbiome highly dynamic and dependent on abiotic factors as well^10^. One of the objectives of this study was to observe the rhizosphere community throughout the cropping season by looking at crop developmental stage specific trends. In this study, we observed significant changes across R1 to R8 (Fig. 1) and all stages in rhizosphere samples (data not shown) for both number of observed ASVs and Shannon diversity. However, the majority of these variations were due to the S stage samples and M stage samples. The effect of these two stages is visible by looking at the number of significant phyla across developmental stages (Table S5, Figure S8). Such changes were also observed at genus level. For example, PGP organisms like *Bacillus* had comparatively higher abundance in N stage followed by PN and F stages of crop cycle. Notably, abundance of *Bacillus* was least during S and M stage of crop cycle. *Bacillus* are known for their Phosphate-solubilising efficiency which explains their role and presence in higher amount during PN, N and F stages^54,55^. Similar trends were also observed for *Acinetobacter* which are reported to possess several PGP activities^56^. Modulation of rhizosphere microbes has commonly been attributed to change in soil pH^21,57^. In our study, we observed a strong significant link of pH and EC with rhizosphere microbiota similar to previous studies^18,20^. Low pH (< 8) and high EC (> 1.1) was observed during germination of seeds among all rhizosphere samples. While significant changes were observed in the diversity among rhizosphere samples, this was not reflected in DESeq2 based differential abundances. The observation from DESeq2 enrichment corroborates that the differences in the microbiome during the crop cycle are minimal, but these changes are more prominent when compared with PS and PH soil microbiome. Moreover, changes were observed for M stage sample which was much different than previous stage samples and started exhibiting likeness to the bulk soil samples (decrease of Firmicutes and increase of Acidobacteriota and Planctomycetota). Overall, the observations from DESeq2 enrichment and significant differences among taxa indicate that developmental stage dependent effects are more prominent during early or germinative stage and during late or maturation stages and includes recruitment of beneficial and specific taxa . The differences in the intermediate stages, including PN, N, and F or the vegetative and production stage of crop are less prominent and involves modulating the abundance of favourable microbes by a minor degree. However, analysing the multiomics data from functional metagenomics and metabolomics can paint a broader and more detailed picture of the interactions in the rhizosphere.

## Methods and materials

### Experimental design and sample collection

The experiment was conducted from the part of farm (~ 13,000 m^2^ area from total farm area of ~ 147,000 m^2^) located near RanaKandorana village (21.618734 N, 69.865700 E) in Gujarat, India during the cropping season of 2017. The farm had history of continuous sowing of G-20 variety of peanut (*Arachis hypogaea* L.) for the previous three years. DAP (Diammonium Phosphate) was added to the farm before sowing the seeds and seeds were irrigated with well water after sowing. The farms in the region followed the practice of early sowing, irrigating once with borewell/well water and then depending on the rainwater for irrigation throughout the season. The selected farm also followed the same practice.

The first samples were collected 15 days prior to sowing of seeds (and five days after addition of Diammonium phosphate fertiliser) (samples termed as PreSowing/PS/Collection-0) (Table 1, Figure S1). During this collection, the field was levelled and tiled ready for sowing of seeds. No intermediate crops were cultivated during the period from last harvest of peanut, making PS soil free from any rhizospheric effects. Next samples were collected 5 days after sowing of seeds, when the seedlings were visible above the ground (samples termed as Collection-1 further grouped into Bulk-1/B1 and Rhizosphere-1/R1). Next samples (Collection-2; Bulk-2/B2; Rhizosphere-2/R2) were collected 6 weeks after Collection-1 because of no rainfall during the period and no irrigation leading to no development in crop (this is common with farms following the practice of early sowing). Next collection (Collection-3; Bulk-3/B3; Rhizosphere-3/R3) was done after 21 days of Collection-2. All subsequent samples (Collection-4 to Collection-8) were collected 14 days after previous collection. Crop was harvested on the very next day of Collection-8. All these samples covered different crop development stages: Seedling (Collection-1), PreNodulating (Collection-2 and Collection-3), Nodulating (Collection-4 and Collection-5), Flowering (Collection-6 and Collection-7) and Matured (Collection-8). Samples were also collected 10 days after harvest of crop (samples termed as PostHarvest/PH/Collection-9). During this collection, the field was completely barren, and samples were collected from the positions where crops were present to check the exact effect on microbiota postharvest.

Rhizosphere (R) and bulk (B) soil samples were collected during all collections from 1 to 8. While PS (Collection-0) and PH (Collection-9) samples were collected from barren farm, B samples were collected from soil between two lines of crop taking care that no roots were visible in dug soil. B soil and samples of PS and PH was collected from depth of 5–10 cm from soil surface. Approximately five gm soil was collected in sterile 50 mL tube for microbiome analysis while around 700gm soil was collected in another sterile container for physicochemical analysis. For collecting the rhizosphere samples, nearby soil was gently brushed aside, and the plants were carefully uprooted. The plants were selected randomly during each collection. The plants were shaken to remove loosely attached soil, which was collected in sterile container for soil property determination. The roots were washed in sterile normal saline (1% NaCl) in flask and the washed soil was then collected in sterile 50 ml falcon tubes for microbiome analysis^14^. During earlier development phases, multiple adjacent plants were uprooted to collect enough soil. The samples for microbiome analysis were transported to lab in − 20 °C portable freezer and then stored at − 20 °C till further processing. The samples for soil property estimation were transported at room temperature. From each collection, 5 samples of rhizosphere soil (n = 40, 8 collections × 5 replicates) and 5 samples of nearby bulk soil (n = 40, 8 collections × 5 replicates) were collected. Also, five replicates were collected for each PS and PH stages making total of 90 samples.

### Sample processing

Soil samples were submitted to a government-approved soil testing facility (Gujarat State Fertiliser Company, GSFC, Vadodara, India) for physicochemical analysis. The samples were tested for physical properties (pH and Electrical Conductivity), macronutrients (%Organic Carbon, concentrations of Phosphate and Potassium) and micronutrient (concentrations of Iron, Sulphur, Manganese, and Zinc and Copper).

The rhizosphere samples for microbiome analysis were thawed and homogenized. The tubes were then centrifuged at 10,000 rpm for 5 min^14^. At this speed, all microbial cells along with soil particles will settle down leaving behind buffer in supernatant which was discarded. The soil was then mixed properly and taken immediately for DNA extraction. Bulk, PreSowing and PostHarvest soil samples were collected without buffer and hence, were processed directly for extraction. DNA was extracted from 1 gm of soil using Qiagen PowerSoil DNA Extraction kit (Qiagen, Germany) following the manufacturer’s instructions.

### Library preparation and sequencing

16S rRNA gene amplicon sequencing libraries were prepared from 12.5 ng DNA as starting material following double-pass PCR protocol as given in Illumina 16S library preparation guide (Illumina, USA). The V3-V4 region of 16S rRNA gene was targeted using 341F and 785R primers fused with Illumina adapters^58^. Libraries were verified on Agilent Bioanalyser (Agilent, USA) and quantified using Qubit v3 (ThermoFisher Scientific, USA). The libraries were sequenced on Illumina MiSeq using 250 × 2 v2 chemistry.

### Data analysis

The raw fastq data was analysed using the Divisive Amplicon Denoising Algorithm 2 (DADA2) pipeline (“dada2” package version 1.14) in R v3.6.1 following the steps given at https://benjjneb.github.io/dada2/tutorial.html^31,59,60^. Taxonomy of ASVs was assigned using multiple databases namely, SILVA v132, RDP (trainset 16, release 11.5), RefSeq + RDP, GTDB v86 and GTDB v89 using the files hosted at zenodo^61–63^. The taxonomy assigned using GTDB v89 was used for all further analysis.

The downstream analysis was done using Phyloseq package v1.30.0 in R v3.6.1 along with other packages like Microbiome v1.9.16, ggpubr v0.2.5, vegan v2.5–6^60,64–67^. In brief, all the data was imported into a Phyloseq object. Alpha diversity was calculated using “alpha” function from Microbiome package. Beta diversity was analysed by Non-Metric Dimensional Scaling (NMDS) on Bray–Curtis distance calculated and plotted using the functions within Phyloseq package. Adonis() function from vegan package and pairwise.adonis() function from pairwiseAdonis package v0.0.1 was used to compare Bray–Curtis distances among groups^68^. Phyloseq package was used to agglomerate taxonomy at phylum and genus levels. ggpubr package was used for comparing different groups. Core-microbiome was determined using the functions in Microbiome package. Differential abundance of taxa was estimated using DESeq2 package v1.26.0 in R along with functions from phyloseq package^69^. All the visualisations were prepared in R using ggplot2 package v3.2.1 along with other packages ggpubr v0.2.5, ggConvexHull v0.1.0, ggnewscale v0.4.1 and ggrepel v0.8.2^70–73^. Other R packages data.table v1.12.8, randomcoloR v1.1.0.1, tidyr v1.0.0, scales v1.1.0 and RColorBrewer v1.1–2 were also used in the analysis^74–78^.

The R script used for analysis is available from github.com/ankit4035/peanutRhizosphere (https://doi.org/10.5281/zenodo.4699352) for reproduction of the entire work. The raw data files can be downloaded from the NCBI SRA (Accessions SRR12732102 to SRR12732191) under Bioproject PRJNA665712.

### Ethics declaration

The study included the use of soil associated with plants. Since no parts of plants were used for the study, no permission was needed for ethical considerations. Furthermore, the owner/farmer was informed about the study and the type of samples to be collected. Verbal consent and permission were obtained to collect the soil from his farm for the work.

## Conclusion

In all, we analysed structure of rhizosphere microbiota from pre-sowing of seeds to post-harvest of crops and compared them with consecutive bulk soil microbiota. Significant variations were observed in alpha-diversity and beta-diversity profiles between bulk and rhizosphere samples, while pre-sowing and post-harvest samples closely resembled bulk soil samples. Differences were also observed among several taxa across all rhizosphere samples especially PGP bacteria. While such patterns demonstrate the “rhizosphere effect”, co-analysing this microbiome patterns with patterns of root exudates could reveal a bigger picture. However, due to lack of resources, we cannot analyse such information in the current study. Nonetheless, the results of this study provide a background for further studies on peanut microbiome.

## Supplementary Information

Supplementary Information.

## Data Availability

The R script used for analysis is available from github.com/ankit4035/peanutRhizosphere (https://doi.org/10.5281/zenodo.4699352) for reproduction of the entire work. The raw data files can be downloaded from the NCBI SRA (Accessions SRR12732102 to SRR12732191) under Bioproject PRJNA665712.
