# Supplementary material for: Characterizing rhizosphere microbiota of peanut (Arachis hypogaea L.) from pre-sowing to post-harvest of crop under field conditions
Source: Sci Rep. 2021 Aug 31;11:17457. doi: 10.1038/s41598-021-97071-3 (PMC8408145; doi:10.1038/s41598-021-97071-3)
Supplement: Supplementary file 1 — Supplementary Information. [file 41598_2021_97071_MOESM1_ESM.pdf]

Characterizing rhizosphere microbiota of peanut (*Arachis hypogaea* L.) from pre-sowing to post-harvest of crop under field conditions

Ankit T. Hinsu[1,2], Ketankumar J. Panchal[2], Ramesh J. Pandit[2], Prakash G. Koringa[2], Ramesh K. Kothari[1]*

1 Department of Biosciences, Saurashtra University, Rajkot -360005, India

2 Department of Animal Biotechnology, College of Veterinary Sciences & A.H., Anand Agricultural University, Anand – 388001, India

*Address for Correspondence:
Prof. Ramesh K. Kothari
Department of Biosciences,
Saurashtra University,
Rajkot – 360005, INDIA
kothari1971@gmail.com, rkkothari@sauuni.ac.in

E-mail addresses of authors:

Ankit T. Hinsu: ankit4035hinsu@gmail.com

Ketankumar J. Panchal: ketan9589@gmail.com

Ramesh J. Pandit: panditrameshj@gmail.com

Prakash G. Koringa: prakashkoringa@gmail.com

Ramesh K. Kothari: kothari1971@gmail.com

Supplementary figures

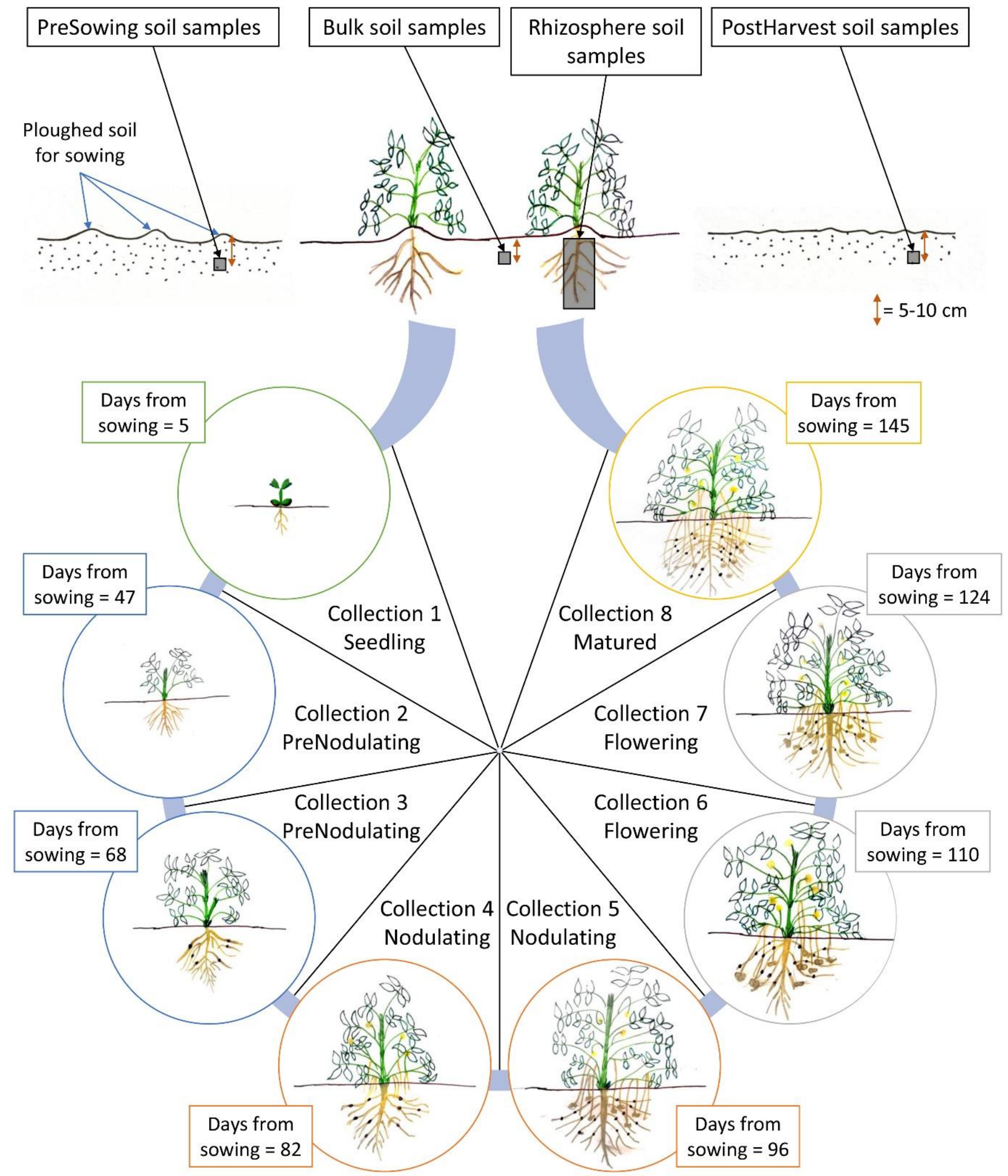

Figure S1: Schematic of experimental design.

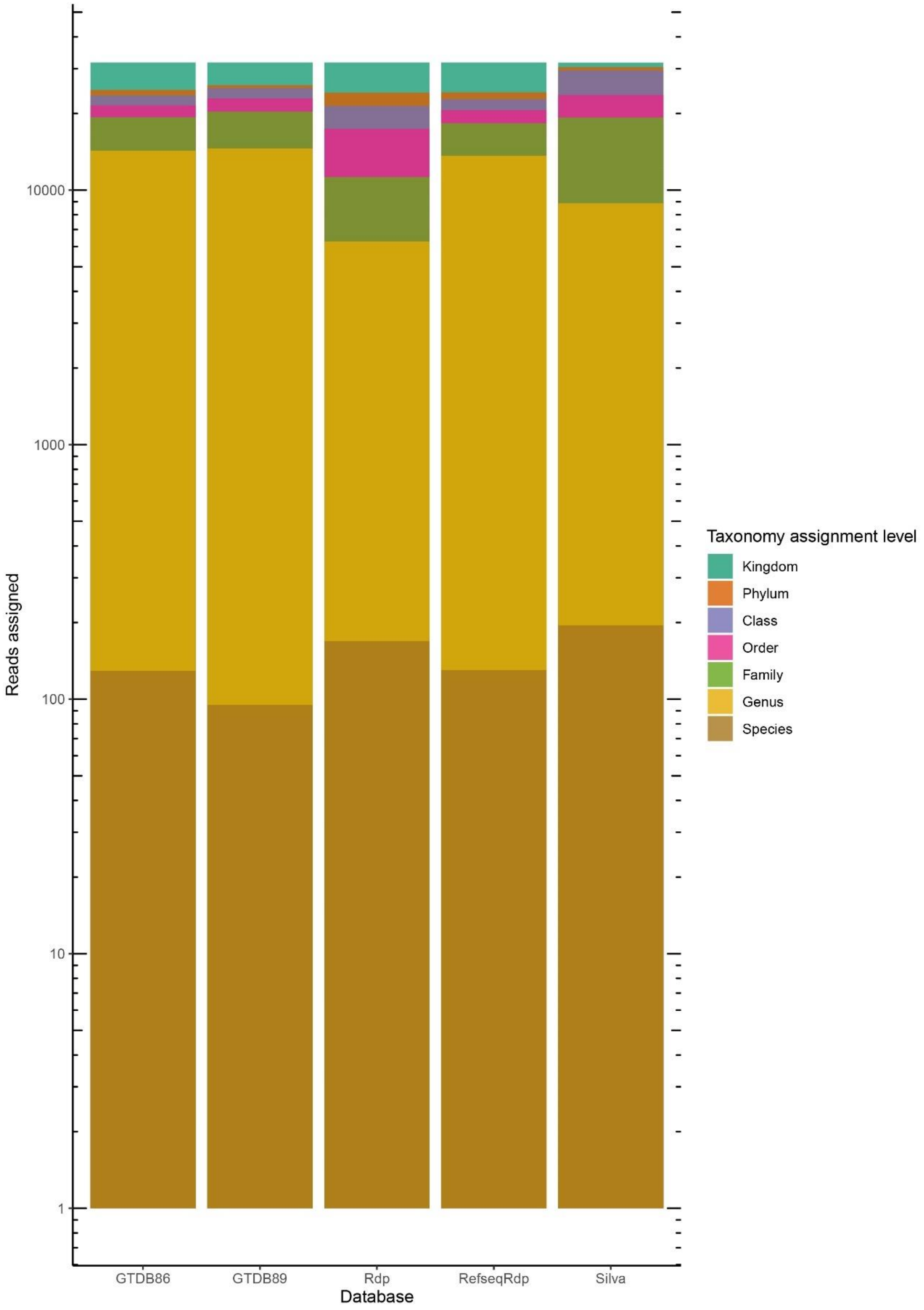

Figure S2: Read annotation distribution across taxonomic levels for five databases. The values on Y-axis is given as logarithmic scale. The plot is prepared as Stacked plot and represent actual value on Y-axis for each taxonomic levels.

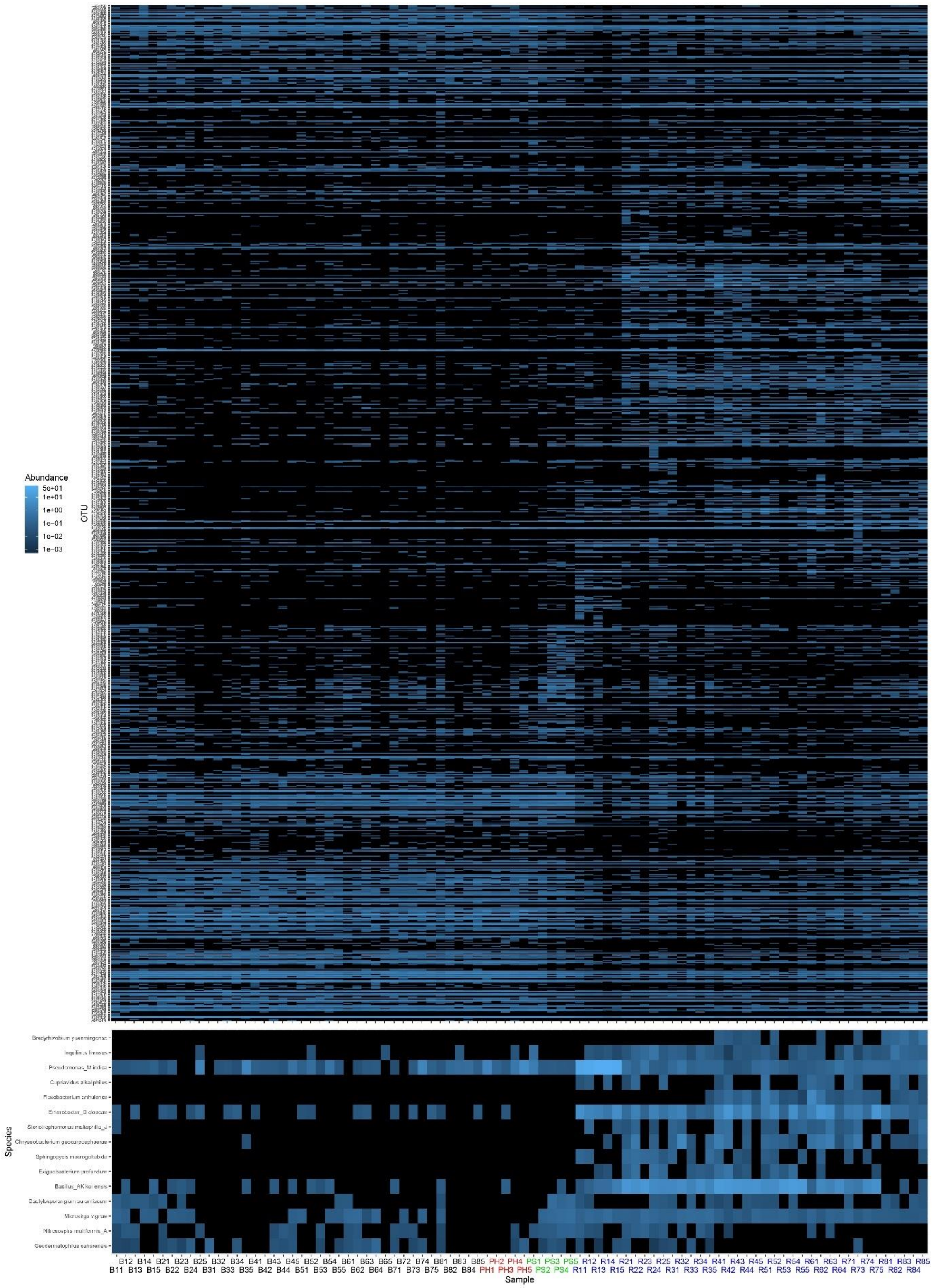

Figure S3: Heatmap representing abundance distribution for all ASV (top) and top ASVs annotated as species (bottom).

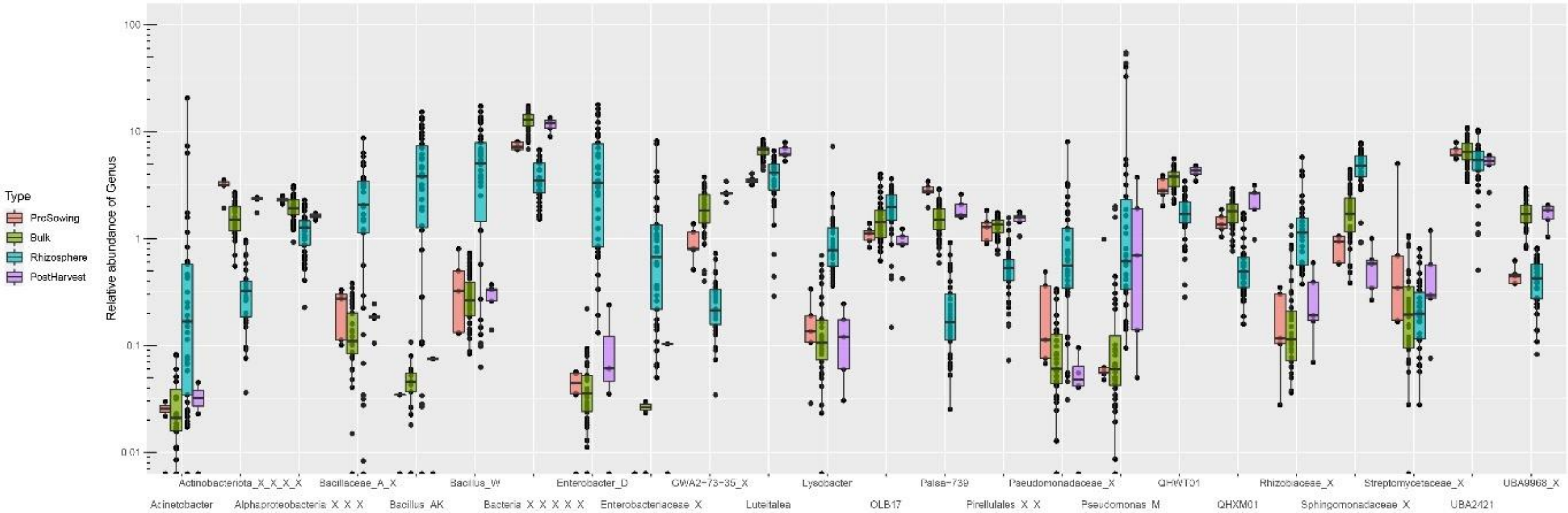

Kruskal-Wallis group test	****	****	****	****	****	****	****	****	****	****	****	****	***	****	****	****	****	****	****	****	****	ns	*	****
PostHarvest vs Bulk	ns	*	ns	ns	ns	ns	ns	ns	ns	*	ns	ns	*	ns	ns	ns	*	ns	ns	ns	***	ns	*	ns
ProSowing vs Bulk	ns	***	*	ns	ns	ns	****	ns	ns	**	****	ns	ns	***	ns	*	ns	ns	ns	ns	**	ns	ns	****
PreSowing vs PostHarvest	ns	ns	**	ns	ns	ns	**	ns	ns	**	**	ns	ns	*	ns	*	ns	*	ns	ns	ns	ns	ns	**
Rhizosphere vs Bulk	****	****	****	****	****	****	****	****	****	****	****	****	*	****	****	****	****	****	****	****	****	ns	**	****
Rhizosphere vs PostHarvest	**	****	**	*	**	*	****	***	***	****	****	****	**	****	****	***	ns	****	****	***	****	ns	ns	****
Rhizosphere vs PreSowing	**	****	****	*	**	*	****	****	***	****	ns	****	**	****	***	ns	**	**	**	****	****	ns	ns	ns

Figure S4: Box-plot distribution across four sample types for top genera. p-values from across-group comparison by Kruskal-Wallis test and each pair of groups by Wilcoxon-test is given in the table below for each genera plotted.

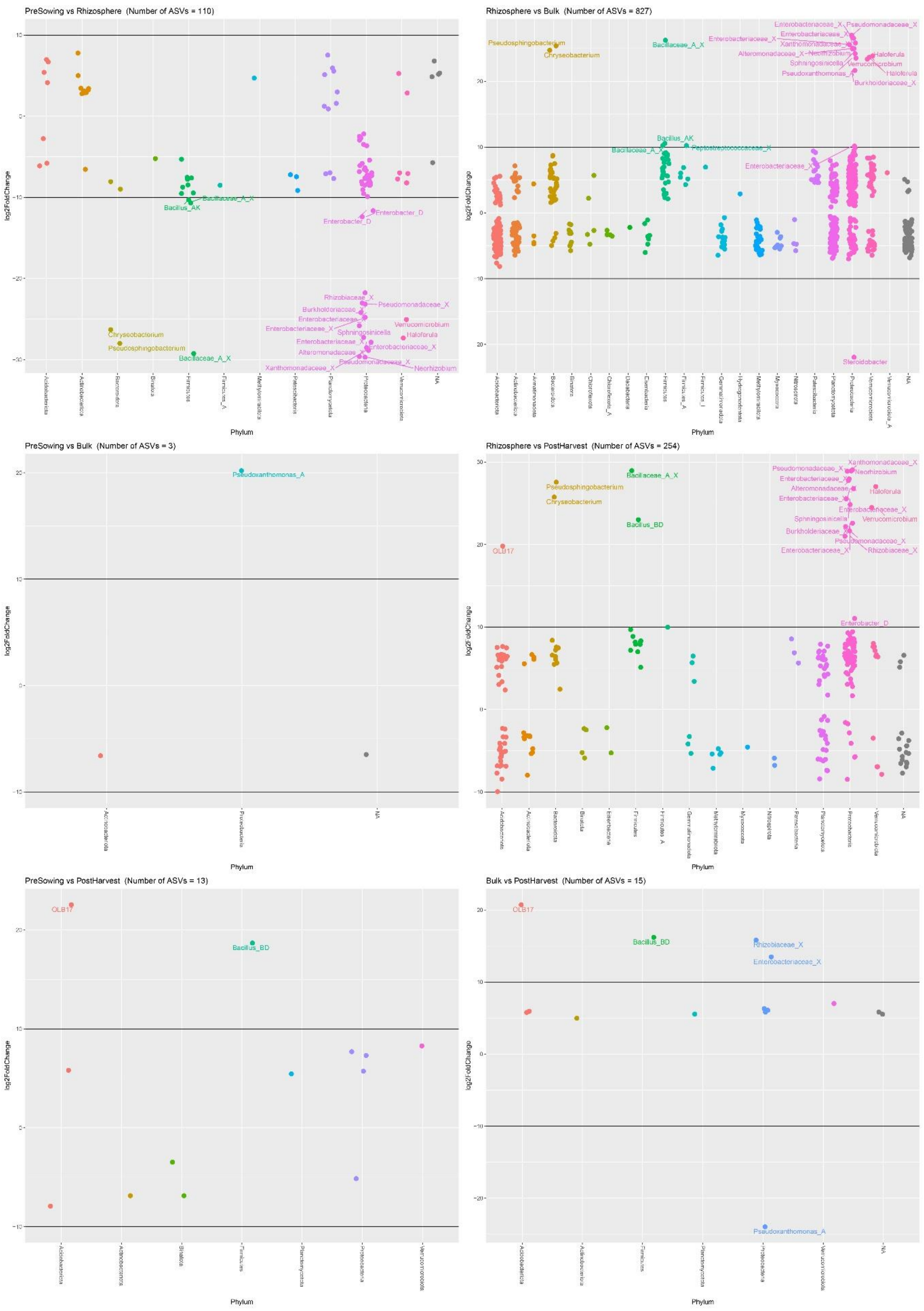

Figure S5: Distribution plot of DESeq2 based log2FoldChange values between all pairs of sample types. Only the significant (p-value < 0.01) changes are plotted. The points are coloured by phylum and labelled if fold change more than 10. Number of significant ASVs are written in the bracket in the title for each comparison.

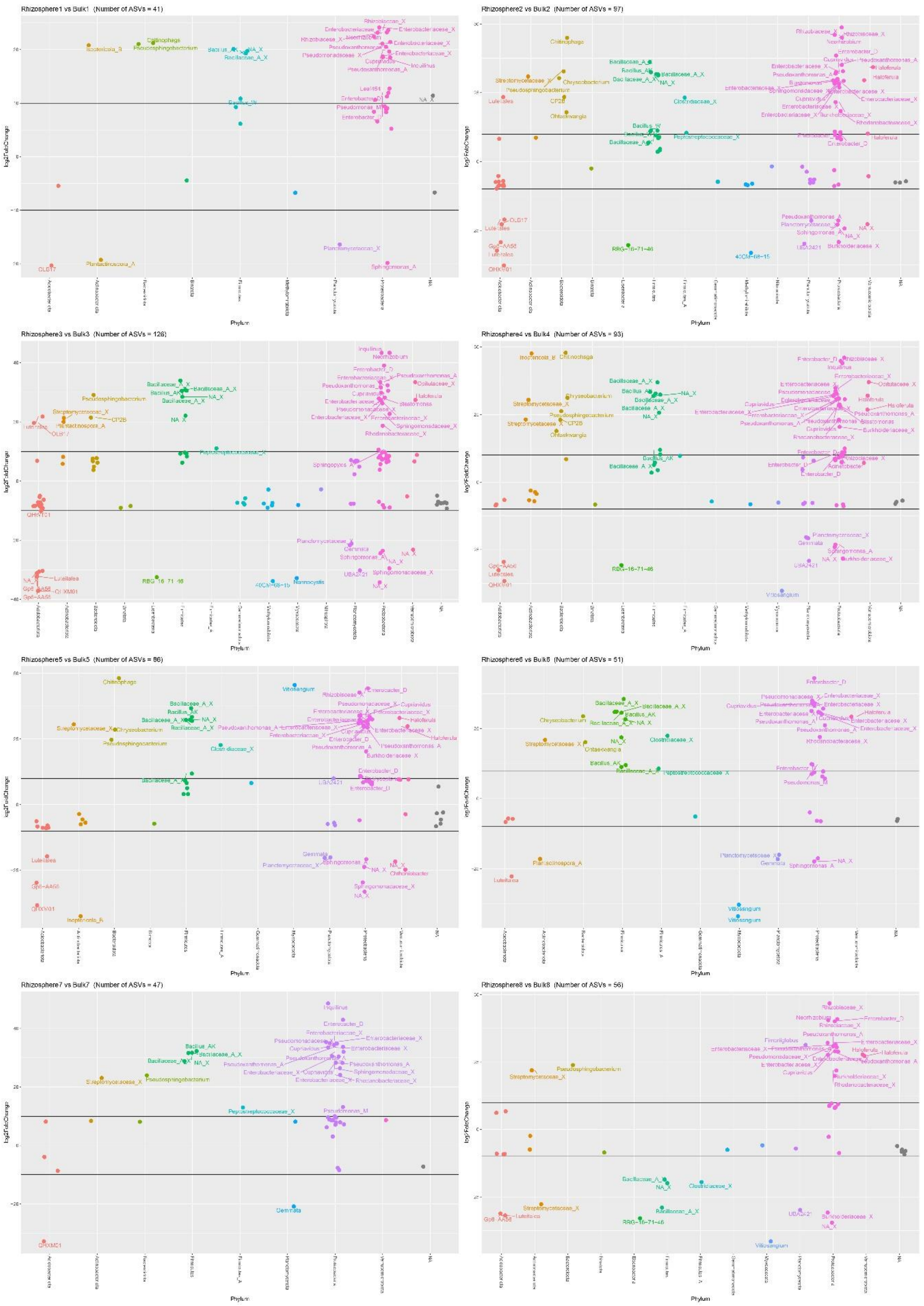

Figure S6: Distribution plot of DESeq2 based log2FoldChange values between rhizosphere and bulk samples of each collection. Only the significant (p-value < 0.01) changes are plotted. The points are coloured by phylum and labelled if fold change more than 10. Number of significant ASVs are written in the bracket in the title for each comparison.

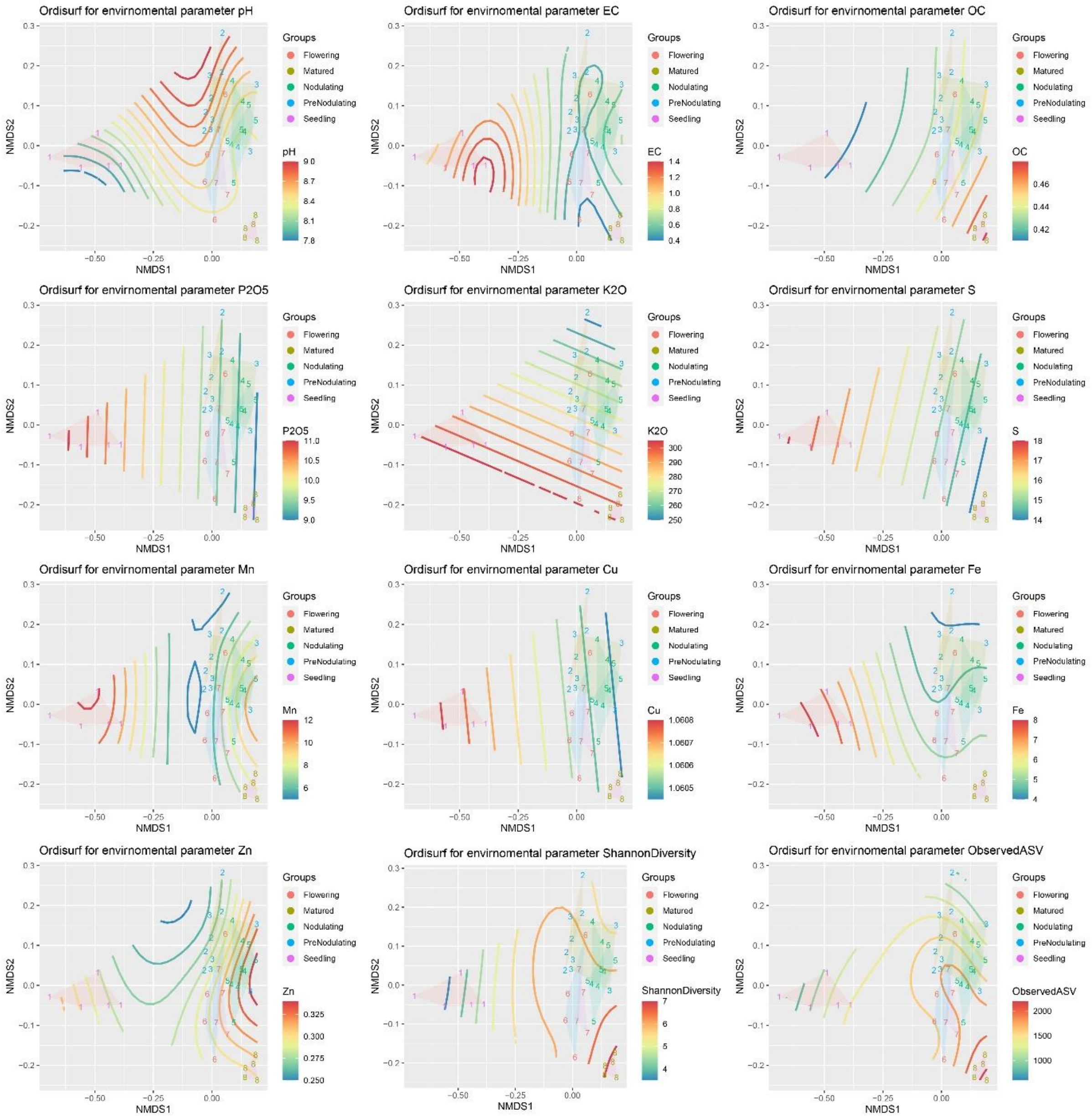

Figure S7: Ordisurf plots for all environmental variable and alpha diversity metrics overlapped on NMDS plot of all Rhizosphere samples.

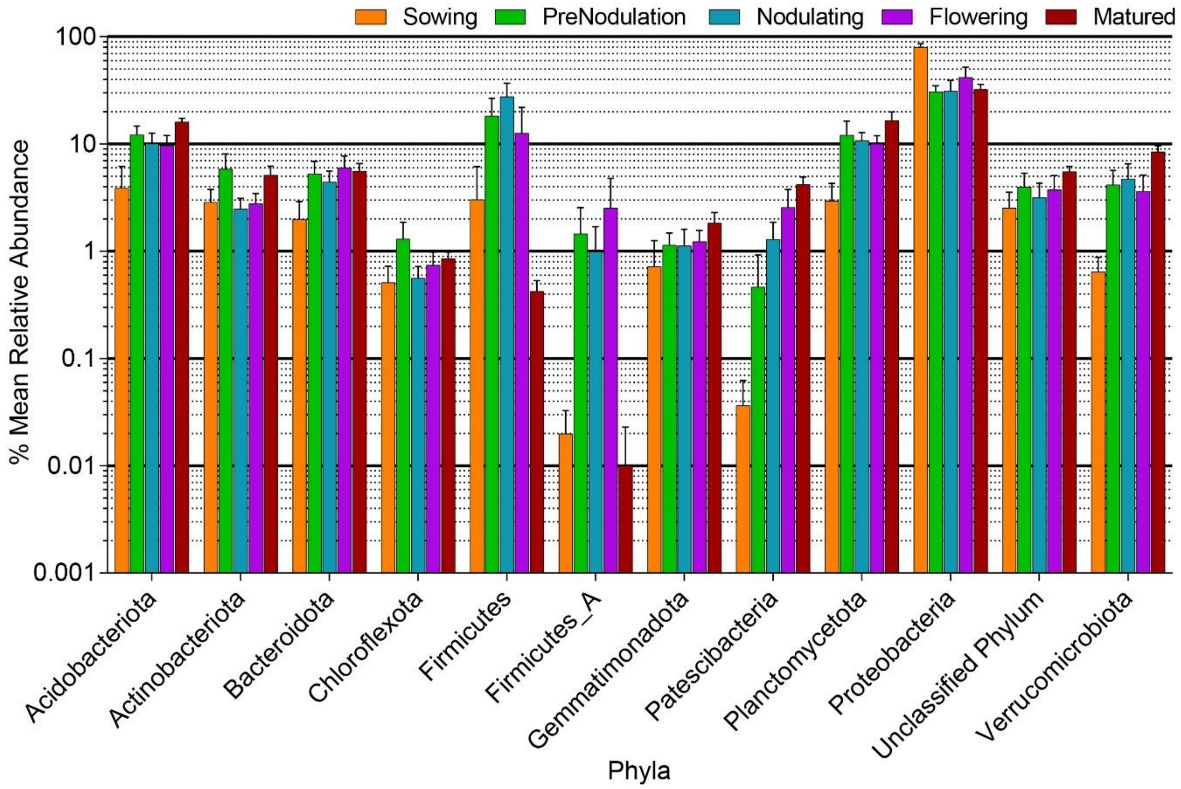

Figure S8: Phylum level distribution of rhizosphere samples across crop developmental stage. Phlya with abundance > 1% in at least one stage are plotted.

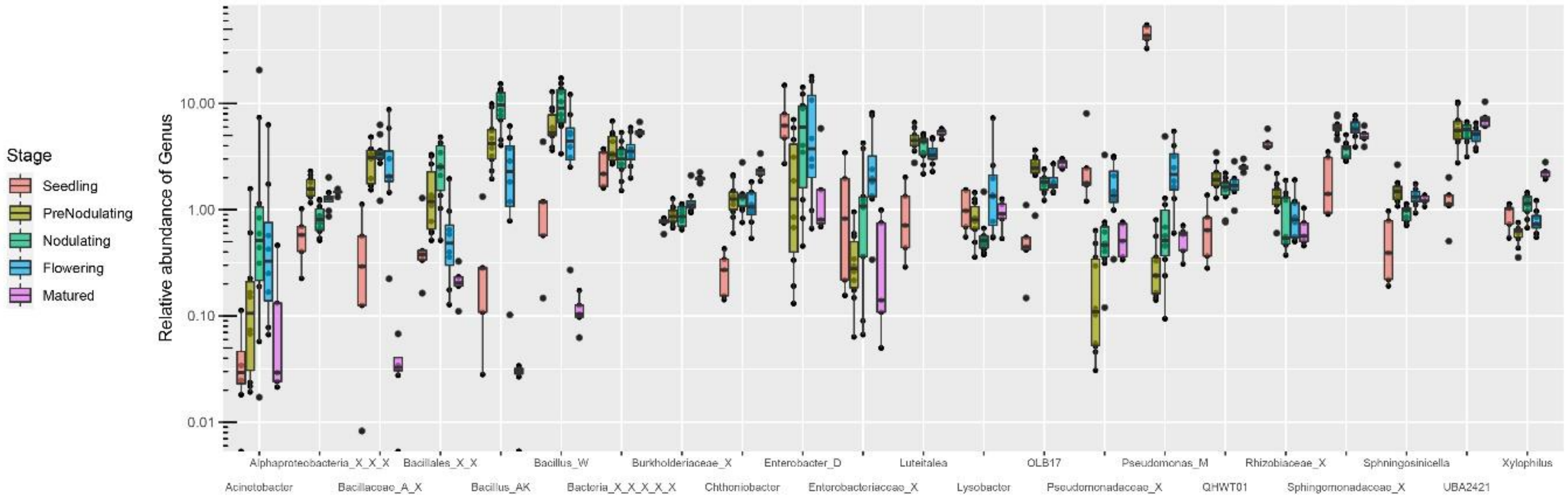

Kruskal–Wallis group test	*	****	***	***	****	****	*	***	***	ns	**	***	*	***	***	****	***	***
Matured vs Flowering	ns	ns	**	ns	**	**	ns	*	**	ns	**	**	ns	*	*	**	*	ns
Nodulating vs Flowering	ns	**	ns	**	***	*	ns	*	ns	ns	ns	ns	**	ns	*	**	ns	ns
Nodulating vs Matured	ns	***	***	***	**	***	**	***	**	ns	ns	**	*	**	ns	ns	***	ns
PreNodulation vs Flowering	ns	*	ns	*	ns	ns	ns	**	ns	ns	***	*	ns	ns	***	****	ns	ns
PreNodulation vs Matured	ns	ns	***	***	**	***	ns	***	**	ns	ns	ns	ns	ns	*	ns	ns	**
PreNodulation vs Nodulating	ns	****	ns	ns	**	ns	ns	ns	ns	ns	ns	ns	ns	*	*	ns	ns	ns
Sowing vs Flowering	**	**	*	ns	*	ns	ns	**	**	ns	ns	**	ns	**	ns	**	**	**
Sowing vs Matured	ns	**	ns	ns	*	*	**	**	**	*	ns	**	ns	**	**	**	**	**
Sowing vs Nodulating	*	ns	***	**	***	**	ns	ns	***	ns	ns	***	*	***	**	***	*	***
Sowing vs PreNodulation	ns	***	***	*	***	**	ns	ns	***	*	ns	***	ns	**	***	***	**	***

Figure S9: Box-plot distribution of rhizosphere samples across all crop developmental stages for top genera. p-values from across-group comparison by Kruskal-Wallis test and each pair of groups by Wilcoxon-test is given in the table below for each genera plotted.

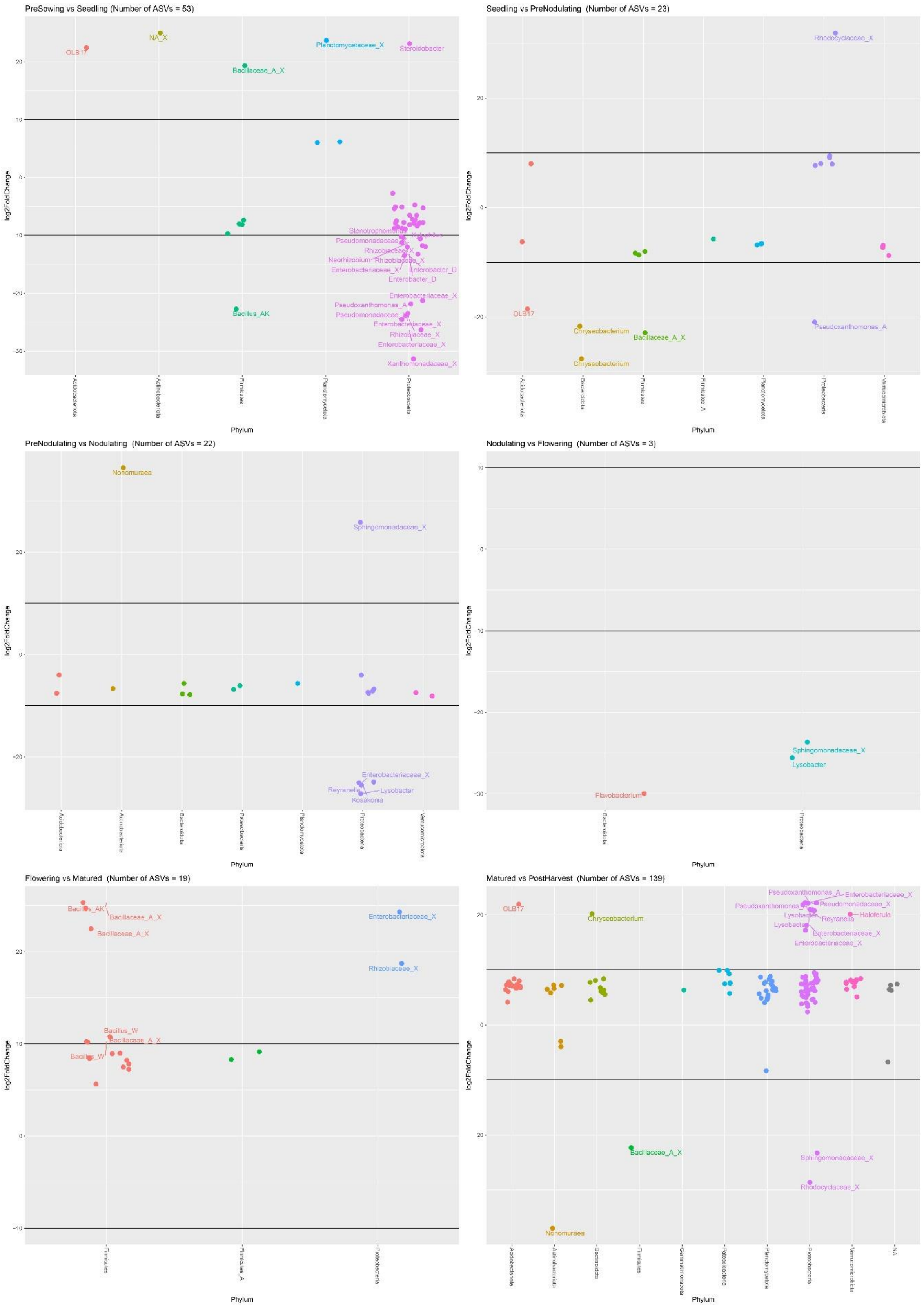

Figure S10: Distribution plot of DESeq2 based log2FoldChange values of rhizosphere samples between pairs of consecutive crop developmental stages. Only the significant (p-value < 0.01) changes are plotted. The points are coloured by phylum and labelled if fold change more than 10. Number of significant ASVs are written in the bracket in the title for each comparison.

Supplementary tables

Table S1: Comparisons of environmental parameters and nutrient concentration across multiple groups.

	**Among types of samples (presowing, bulk, rhizosphere and postharvest),** Kruskal-Wallis p-value	**Between paired samples of bulk and rhizosphere,** paired Wilcoxon p-value	**Among different developmental stages within rhizosphere samples,** Kruskal-Wallis p-value
**pH**	0.00003	0.00001	0.00001
**EC, Electrical Conductivity**	0.00352	0.02565	0.00005
**OC, %Organic Carbon**	0.65250	0.47706	0.00245
**P2O5, Phosphate**	0.08321	0.95868	0.17060
**K2O, Potash**	0.02439	0.90188	0.06741
**S, Sulphur**	0.00172	0.17666	0.00736
**Mn, Manganese**	0.00085	0.12216	0.00011
**Cu, Copper**	0.00783	0.60555	0.91301
**Fe, Iron**	0.00139	0.14659	0.00413
**Zn, Zinc**	0.03587	0.66338	0.86102

Table S2: Kruskal-Wallis test comparison of all genera across four sample types. BH = Benjamini-Hochberg. "_X" are used to represent unannotated taxa at a level, multiple suffixes indicate that many level was the last annotation.

Genus	p-value	BH adjusted p-value
**Pseudomonas_M**	4.65E-09	3.50E-08
**Acinetobacter**	6.17E-10	5.80E-09
**Enterobacter_D**	9.64E-14	3.90E-12
**Bacteria_X_X_X_X_X**	1.06E-14	1.60E-12
**Bacillus_W**	1.63E-06	7.80E-06
**Bacillus_AK**	3.85E-11	4.70E-10
**UBA2421**	1.05E-02	2.50E-02
**Bacillaceae_A_X**	1.14E-06	5.50E-06
**Luteitalea**	6.62E-12	1.00E-10
**Enterobacteriaceae_X**	3.41E-16	2.40E-13
**Pseudomonadaceae_X**	3.63E-10	3.50E-09
**Sphingomonadaceae_X**	3.16E-11	4.00E-10
**Lysobacter**	1.60E-12	3.00E-11
**Rhizobiaceae_X**	2.68E-11	3.60E-10
**QHWT01**	1.01E-12	2.20E-11
**Streptomycetaceae_X**	9.68E-02	1.70E-01
**Bacillales_X_X**	5.70E-04	1.80E-03
**OLB17**	9.75E-04	2.80E-03
**GWA2-73-35_X**	2.64E-14	2.00E-12
**Actinobacteriota_X_X_X_X**	1.33E-14	1.70E-12
**Peptostreptococcaceae_X**	2.68E-10	2.70E-09
**Palsa-739**	1.69E-14	1.70E-12
**Chthoniobacter**	2.48E-04	8.30E-04
**Opitutaceae_X**	1.06E-02	2.50E-02
**QHXM01**	1.29E-11	1.90E-10
**Alphaproteobacteria_X_X_X**	2.36E-08	1.60E-07
**Flavobacterium**	2.10E-10	2.20E-09
**Haloferula**	5.45E-11	6.50E-10
**UBA9968_X**	4.73E-14	2.60E-12
**Burkholderiales_X_X**	9.28E-14	3.90E-12
**Micromonosporaceae_X**	1.19E-06	5.80E-06
**Pseudosphingobacterium**	1.21E-07	6.80E-07
**Xylophilus**	3.06E-05	1.20E-04
**UBA11741**	1.88E-13	5.60E-12
**Sphningosinicella**	3.13E-06	1.40E-05
**Xanthomonadaceae_X**	9.05E-07	4.50E-06
**Verrucomicrobium**	7.10E-12	1.10E-10
**Microvirga**	1.09E-02	2.50E-02
**Chryseobacterium**	5.08E-15	1.20E-12
**Acidimicrobiia_X_X_X**	2.37E-09	2.00E-08
**Bacillus_Y**	5.22E-10	4.90E-09
**Burkholderiaceae_X**	7.26E-08	4.30E-07
**Pirellula**	1.44E-11	2.10E-10
**Gp6-AA56**	4.58E-12	7.30E-11
**Steroidobacter**	2.10E-03	5.70E-03
**Vitiosangium**	1.21E-08	8.40E-08
**Planctomycetaceae_X**	1.47E-04	5.10E-04
**Vicinamibacterales_X_X**	4.58E-14	2.60E-12

**Fimbriiglobus**	2.57E-09	2.10E-08
**Saccharimonadaceae_X**	7.82E-06	3.30E-05
**Sphingopyxis_A**	1.93E-14	1.70E-12
**UBA6082**	4.10E-09	3.10E-08
**Pontibacter**	1.60E-08	1.10E-07
**GCA-2746885**	1.93E-07	1.10E-06
**Sphingomonas_A**	4.53E-05	1.70E-04
**Clostridiaceae_X**	2.07E-03	5.60E-03
**Nonomuraea**	5.17E-04	1.60E-03
**Pirellulales_X_X**	1.83E-11	2.50E-10
**AR5**	1.09E-12	2.20E-11
**Neorhizobium**	1.86E-14	1.70E-12
**Pseudoxanthomonas_A**	3.37E-10	3.30E-09
**Clostridium**	3.01E-09	2.40E-08
**Gemmatimonadaceae_X**	8.85E-12	1.30E-10
**GWC2-73-18**	1.59E-09	1.40E-08
**Ohtaekwangia**	4.80E-02	9.20E-02
**UKL13-2**	6.10E-02	1.10E-01
**Geodermatophilus**	3.26E-03	8.40E-03
**FW-11**	6.26E-06	2.70E-05
**Dongia**	5.00E-06	2.20E-05
**JKG1**	1.53E-03	4.30E-03
**Pirellulaceae_X**	9.05E-02	1.60E-01
**Arboricoccus**	5.82E-11	6.70E-10
**Mycoplana**	1.26E-13	4.30E-12
**Flavisolibacter**	5.57E-08	3.40E-07
**Leaf454**	3.59E-07	1.90E-06
**Sphingobacterium**	4.35E-12	7.10E-11
**Gemmata**	1.11E-09	1.00E-08
**Pedosphaerales_X_X**	2.05E-01	3.20E-01
**Clostridium_AD**	8.88E-07	4.40E-06
**Actinobacteria_X_X_X**	1.00E-13	3.90E-12
**Alteromonadaceae_X**	7.55E-02	1.40E-01
**Adhaeribacter**	8.53E-03	2.10E-02
**Nannocystis**	8.64E-01	8.80E-01
**QHVH01**	6.94E-14	3.40E-12
**Bryobacteraceae_X**	3.17E-11	4.00E-10
**Pedosphaeraceae_X**	4.03E-02	8.00E-02
**Rhodocyclaceae_X**	1.72E-09	1.50E-08
**UBA1161_X**	1.88E-01	2.90E-01
**Geminicoccaceae_X**	4.53E-13	1.20E-11
**SCN-69-37**	1.11E-13	4.00E-12
**Lysobacter_A**	4.14E-12	6.80E-11
**Solirubrobacter**	9.98E-14	3.90E-12
**AR19**	1.40E-12	2.70E-11
**Aromatoleum**	1.76E-02	3.90E-02
**Gemmataceae_X**	5.72E-11	6.70E-10
**UBA4664**	7.81E-06	3.30E-05
**Pedosphaera**	2.95E-05	1.20E-04
**Planobispora**	2.35E-01	3.50E-01
**Cellvibrio**	1.49E-10	1.60E-09
**Chitinophagaceae_X**	7.63E-05	2.70E-04

**SZUA-115**	3.96E-07	2.10E-06
**Planctomycetes_X_X_X**	2.98E-13	8.60E-12
**PALSA-1355**	3.21E-12	5.60E-11
**Stenotrophomonas**	2.15E-14	1.70E-12
**Gp7-AA10**	7.02E-08	4.20E-07
**Solirubrobacteraceae_X**	1.01E-12	2.20E-11
**Bacilli_X_X_X**	5.28E-05	2.00E-04
**ARS98**	8.08E-14	3.80E-12
**Rubrobacter_B**	1.03E-11	1.50E-10
**Bacillus_BD**	5.92E-03	1.50E-02
**Thauera**	4.01E-02	8.00E-02
**Catelliglobosispora**	1.96E-03	5.40E-03
**Steroidobacteraceae_X**	1.72E-04	5.90E-04
**Gemmatimonadales_X_X**	1.48E-08	1.00E-07
**Kosakonia**	3.91E-02	7.90E-02
**Cupriavidus**	3.36E-14	2.30E-12
**Oscillochloris**	1.95E-01	3.00E-01
**UBA4665_X**	4.48E-08	2.80E-07
**Pyrinomonadaceae_X**	1.70E-12	3.20E-11
**RBG-16-71-46**	9.68E-13	2.20E-11
**Opitutus**	3.64E-05	1.40E-04
**Thermomicrobiaceae_X**	2.33E-06	1.10E-05
**2-12-FULL-64-23**	2.15E-09	1.80E-08
**Chitinophaga**	6.95E-10	6.50E-09
**Isosphaeraceae_X**	5.33E-07	2.70E-06
**Myxococcus**	6.86E-02	1.30E-01
**Niastella**	1.05E-02	2.50E-02
**Mycobacteriales_X_X**	2.54E-06	1.20E-05
**Gammaproteobacteria_X_X_X**	1.04E-05	4.30E-05
**Polyangiales_X_X**	8.50E-04	2.50E-03
**UBA10511**	2.96E-04	9.80E-04
**Plantactinospora_A**	8.23E-08	4.80E-07
**Nitrospira_C**	2.93E-11	3.80E-10
**Thermoleophilia_X_X_X**	3.76E-14	2.40E-12
**Phenylobacterium**	1.19E-03	3.40E-03
**Bacillus_AA**	2.13E-08	1.40E-07
**Methylibium**	1.86E-03	5.10E-03
**AV2**	1.99E-12	3.60E-11
**40CM-3-62-11**	2.22E-12	4.00E-11
**Pararheinheimera**	8.02E-01	8.40E-01
**BOG-1338**	2.89E-07	1.60E-06
**Exiguobacterium**	3.32E-07	1.80E-06
**Methylophilaceae_X**	8.12E-13	2.00E-11
**Acidobacteriota_X_X_X_X**	3.42E-11	4.20E-10
**Gemmatirosa**	1.65E-02	3.70E-02
**SCN-70-22**	8.91E-13	2.10E-11
**Rhizobium**	5.36E-16	2.40E-13
**Erwinia**	2.01E-02	4.30E-02
**Nocardioidaceae_X**	1.64E-02	3.70E-02
**Arthrobacter_I**	4.33E-09	3.30E-08
**CP2B**	2.83E-03	7.40E-03
**Achromobacter**	1.69E-10	1.80E-09

**Flavobacterium_A**	1.97E-02	4.30E-02
**BOG-1460**	5.30E-13	1.30E-11
**40CM-68-15**	2.63E-11	3.50E-10
**Pseudoxanthomonas**	4.30E-03	1.10E-02
**Rubrobacteraceae_X**	4.57E-08	2.80E-07
**Zavarzinella**	9.81E-02	1.70E-01
**SM1A02_X**	9.63E-13	2.20E-11
**Paenibacillaceae_X**	1.79E-01	2.80E-01
**Byssovorax**	1.05E-13	3.90E-12
**Haliangium**	4.19E-04	1.40E-03
**Dactylosporangium**	1.98E-07	1.10E-06
**Inquilinus**	3.27E-09	2.60E-08
**Paramesorhizobium**	9.69E-03	2.30E-02
**Aeromonas**	1.98E-04	6.70E-04
**Terrimicrobium**	2.96E-10	3.00E-09
**Schlesneria**	3.57E-13	9.60E-12
**G233**	9.95E-08	5.70E-07
**Acidimicrobiales_X_X**	1.45E-01	2.30E-01
**Bacteroidia_X_X_X**	4.91E-02	9.40E-02
**Streptacidiphilus**	1.36E-01	2.30E-01
**Nevskiaceae_X**	1.03E-04	3.60E-04
**UBA6960_X**	5.97E-14	3.10E-12
**UBA2386**	9.02E-08	5.20E-07
**SZUA-42**	5.50E-08	3.40E-07
**Kouleothrix**	5.59E-06	2.40E-05
**SZUA-252**	3.42E-11	4.20E-10
**Pirellula_B**	4.49E-04	1.40E-03
**Jiangella**	1.31E-12	2.60E-11
**Bin18**	7.72E-07	3.90E-06
**Saprospiraceae_X**	1.50E-02	3.40E-02
**Palsa-1233**	6.27E-03	1.60E-02
**Phycisphaerae_X_X_X**	2.94E-06	1.30E-05
**Ilumatobacter_A**	1.78E-03	4.90E-03
**UBA11883**	9.21E-11	1.00E-09
**Dehalococcoidia_X_X_X**	1.03E-10	1.10E-09
**Rhizobiales_X_X**	3.65E-02	7.50E-02
**Polyangiaceae_X**	1.07E-12	2.20E-11
**UBA5946**	3.29E-13	9.20E-12
**Cyanobacteriia_X_X_X**	1.48E-02	3.40E-02
**Bordetella_A**	1.50E-08	1.00E-07
**Bradyrhizobium**	1.98E-04	6.70E-04
**Fictibacillus_B**	1.21E-07	6.80E-07
**Noviherbaspirillum**	5.58E-06	2.40E-05
**Citrobacter**	7.25E-01	7.90E-01
**Chthoniobacterales_X_X**	7.07E-10	6.50E-09
**Chitinophagales_X_X**	4.03E-09	3.10E-08
**PMNU01**	1.59E-01	2.50E-01
**QHBO01**	5.73E-09	4.20E-08
**P52-10**	1.50E-02	3.40E-02
**Burkholderia**	5.90E-06	2.50E-05
**Azospirillum**	5.78E-03	1.40E-02
**Alishewanella**	5.69E-01	6.80E-01

**Gemmatimonadetes_X_X_X**	3.12E-09	2.50E-08
**Novosphingobium**	4.47E-01	5.60E-01
**Dyadobacter**	6.60E-15	1.20E-12
**UBA1020**	9.40E-07	4.70E-06
**Acidobacteriales_X_X**	2.47E-08	1.60E-07
**MM2**	6.72E-15	1.20E-12
**SWB02**	4.43E-07	2.30E-06
**Geodermatophilaceae_X**	7.62E-03	1.90E-02
**Bin134**	1.51E-03	4.20E-03
**Saccharimonadales_X_X**	8.08E-08	4.70E-07
**Domibacillaceae_X**	5.11E-01	6.20E-01
**Roseimaritima**	2.43E-01	3.60E-01
**Aeromicrobium**	1.49E-02	3.40E-02
**Saccharibacillus**	7.25E-01	7.90E-01
**UBA4655**	1.40E-09	1.20E-08
**Coleofasciculus**	1.79E-02	4.00E-02
**Blastomonas**	2.06E-09	1.70E-08
**Palsa-89**	1.23E-01	2.10E-01
**Chloroflexota_X_X_X_X**	1.58E-03	4.40E-03
**Polymorphum**	7.83E-01	8.20E-01
**Blastococcus**	2.05E-02	4.40E-02
**SZUA-320**	8.93E-09	6.30E-08
**Nitrospira_A**	2.42E-06	1.10E-05
**Rhodobacteraceae_X**	2.28E-01	3.40E-01
**IMCC26207**	2.82E-06	1.30E-05
**55-13**	1.13E-04	3.90E-04
**GCA-2723275**	1.31E-07	7.30E-07
**LHW63021**	3.66E-01	5.00E-01
**Nocardioides**	5.03E-04	1.60E-03
**Isoptericola_B**	3.34E-03	8.60E-03
**ZC4RG30**	5.64E-04	1.80E-03
**Cyanobacteriales_X_X**	1.14E-02	2.60E-02
**Terrimonas**	7.60E-04	2.30E-03
**Planctomicrobium**	3.46E-12	5.80E-11
**Hyalangium**	7.13E-03	1.70E-02
**Acetobacteraceae_X**	3.58E-08	2.30E-07
**Reyranella**	4.49E-01	5.60E-01
**Arenimonas**	3.65E-10	3.50E-09
**Chthonomonas**	2.64E-09	2.10E-08
**Pseudomonas_A**	7.88E-06	3.30E-05
**Taibaiella_A**	3.58E-05	1.40E-04
**Azohydromonas**	6.24E-04	1.90E-03
**Sphingopyxis**	3.32E-07	1.80E-06
**Verrucomicrobiae_X_X_X**	5.80E-01	6.90E-01
**Rhizorhabdus**	4.79E-02	9.20E-02
**Devosia**	7.65E-02	1.40E-01
**Cryptosporangium**	1.56E-03	4.30E-03
**Chryseolinea_A**	5.90E-06	2.50E-05
**Prosthecobacter**	9.85E-07	4.90E-06
**Rhodospirillum_A**	6.59E-02	1.20E-01
**SZUA-318**	1.48E-13	4.90E-12
**Pseudomonas_F**	2.98E-05	1.20E-04

**Rubellimicrobium**	2.32E-03	6.20E-03
**Sorangium**	2.72E-04	9.10E-04
**GCA-002686595_X**	5.07E-10	4.90E-09
**Planctomyces_A**	5.18E-05	1.90E-04
**Pseudomonas_B**	8.60E-01	8.80E-01
**Chryseolinea**	8.22E-02	1.50E-01
**Bosea**	1.90E-10	2.00E-09
**Blastocatellia_X_X_X**	7.17E-04	2.20E-03
**Azoarcus_B**	3.09E-05	1.20E-04
**Herpetosiphon**	6.19E-05	2.30E-04
**NIC37A-2**	2.05E-11	2.80E-10
**Rhodanobacteraceae_X**	2.00E-05	8.10E-05
**Pseudonocardia**	1.72E-04	5.90E-04
**KBS-96**	6.66E-05	2.40E-04
**Xanthobacteraceae_X**	5.11E-01	6.20E-01
**GWC2-71-9_X**	6.86E-04	2.10E-03
**Cystobacter**	1.72E-01	2.70E-01
**NS-102**	5.70E-01	6.80E-01
**Pseudomonas_H**	2.57E-01	3.70E-01
**Flavisolibacter_B**	3.08E-08	2.00E-07
**GWC2-70-10**	1.82E-11	2.50E-10
**Aneurinibacillus_A**	2.67E-02	5.60E-02
**QKVK01**	7.18E-02	1.30E-01
**Anaeromyxobacter**	3.23E-01	4.50E-01
**67-14**	3.33E-04	1.10E-03
**Streptomyces_D**	6.87E-02	1.30E-01
**HRBIN30**	9.12E-10	8.30E-09
**Par-f-2**	9.62E-09	6.70E-08
**Myxococcaceae_X**	3.20E-02	6.60E-02
**UBA5793**	1.56E-10	1.70E-09
**Z2-YC6860**	1.15E-02	2.70E-02
**Proteobacteria_X_X_X_X**	2.90E-02	6.00E-02
**Cnuella**	3.23E-02	6.60E-02
**Euzebya**	1.09E-12	2.20E-11
**UBA4720**	2.53E-03	6.70E-03
**Isosphaera**	7.33E-04	2.20E-03
**Elioraea**	8.83E-05	3.10E-04
**Caulobacteraceae_X**	8.93E-04	2.60E-03
**GCA-2683825**	1.82E-13	5.60E-12
**Root149**	3.91E-02	7.90E-02
**Binatia_X_X_X**	6.54E-09	4.70E-08
**Anaerolineae_X_X_X**	7.68E-08	4.60E-07
**Caulobacter**	2.28E-02	4.90E-02
**Sandaracinus**	6.47E-09	4.60E-08
**URHD0088**	2.97E-04	9.80E-04
**UBA11740**	5.74E-07	2.90E-06
**BD2-11**	3.67E-08	2.40E-07
**IMCC26256**	3.48E-12	5.80E-11
**Agromyces**	1.32E-06	6.30E-06
**Singulisphaera**	1.81E-02	4.00E-02
**Mycolicibacterium**	7.94E-08	4.70E-07
**Ramlibacter**	4.53E-04	1.50E-03

**Fen-1342**	6.38E-09	4.60E-08
**UBA4093**	8.69E-01	8.90E-01
**Nitrospiraceae_X**	3.20E-07	1.70E-06
**Chloroflexales_X_X**	1.02E-06	5.00E-06
**Planctomycetota_X_X_X_X**	1.30E-09	1.20E-08
**Berkiella**	3.08E-06	1.40E-05
**Lentzea**	1.10E-06	5.30E-06
**Catenuloplanes**	5.80E-05	2.10E-04
**Domibacillus**	4.21E-01	5.60E-01
**Herminiimonas**	8.53E-05	3.00E-04
**Yuhushiella**	2.98E-01	4.20E-01
**Gp18-AA60**	4.40E-11	5.30E-10
**W-Chloroflexi-9**	6.51E-08	3.90E-07
**Tumebacillus_A**	2.18E-02	4.70E-02
**Paracoccus**	8.43E-11	9.50E-10
**Brevundimonas**	1.28E-04	4.50E-04
**Delftia**	2.32E-01	3.50E-01
**Hungatella**	4.47E-01	5.60E-01
**Fluviicola**	2.14E-02	4.60E-02
**Bog-159**	2.29E-07	1.30E-06
**Spirillospora**	9.64E-02	1.70E-01
**Geobacterales_X_X**	1.39E-01	2.30E-01
**Ralstonia**	7.55E-02	1.40E-01
**Blastococcus_A**	2.13E-03	5.80E-03
**Acidobacteriaceae_X**	3.04E-01	4.30E-01
**UBA6092**	8.31E-05	3.00E-04
**Idiomarina**	2.57E-01	3.70E-01
**UBA7805**	2.06E-01	3.20E-01
**CSP1-2_X**	1.87E-10	2.00E-09
**Rhodococcus**	4.47E-01	5.60E-01
**HGW-BRC1-1**	3.38E-01	4.60E-01
**Caldilineales_X_X**	1.02E-01	1.80E-01
**Bacillaceae_H_X**	3.82E-01	5.20E-01
**UBA1568**	3.58E-01	4.90E-01
**UBA1268_X**	3.43E-05	1.30E-04
**Acidimicrobiaceae_X**	7.79E-02	1.40E-01
**Minicystis**	4.66E-03	1.20E-02
**Litorilinea**	2.56E-04	8.50E-04
**UTCFX2**	2.03E-06	9.60E-06
**Luteimonas**	1.92E-01	3.00E-01
**Geobacteraceae_X**	1.65E-01	2.60E-01
**Azospirillaceae_X**	1.75E-01	2.80E-01
**Sphingobium**	2.32E-06	1.10E-05
**MEBICO9517**	1.66E-08	1.10E-07
**Methylophilus**	2.65E-05	1.10E-04
**Bacillus_X**	7.25E-01	7.90E-01
**Caldilinea**	1.28E-03	3.60E-03
**Zobellella_B**	4.47E-01	5.60E-01
**Mizugakiibacter**	2.76E-03	7.20E-03
**Mucilaginibacter**	5.84E-04	1.80E-03
**Shinella**	4.47E-01	5.60E-01
**Solitalea**	1.00E-03	2.90E-03

**Ferrovibrio**	2.71E-03	7.10E-03
**Cytophagales_X_X**	5.23E-06	2.30E-05
**Xanthomonadales_X_X**	7.11E-05	2.60E-04
**Anaerolineales_X_X**	7.03E-04	2.20E-03
**Massilia_B**	2.17E-09	1.80E-08
**A52C2**	8.28E-08	4.80E-07
**Flavihumibacter**	5.50E-09	4.10E-08
**R-RK-3**	3.93E-02	7.90E-02
**Altererythrobacter_A**	1.93E-01	3.00E-01
**Parapedobacter**	2.57E-01	3.70E-01
**Nitrosospira**	3.25E-03	8.40E-03
**Arthrobacter_F**	1.17E-03	3.30E-03
**Frateuria_A**	3.25E-04	1.10E-03
**Nocardia**	3.22E-01	4.50E-01
**OLB7**	1.32E-01	2.20E-01
**EW11**	3.85E-03	9.80E-03
**Actinocorallia**	1.37E-01	2.30E-01
**Aquamicrobium**	4.95E-01	6.00E-01
**Labilithrix**	6.36E-08	3.90E-07
**Ga0074140**	2.77E-09	2.20E-08
**Frankia**	2.57E-01	3.70E-01
**Franconibacter**	1.42E-01	2.30E-01
**Microbacteriaceae_X**	5.86E-07	3.00E-06
**Sulfuricaulis**	6.52E-04	2.00E-03
**Pseudoduganella**	8.19E-03	2.00E-02
**Beijerinckiaceae_X**	2.68E-02	5.60E-02
**UBA12294**	3.67E-01	5.00E-01
**Paceibacteria_X_X_X**	9.15E-03	2.20E-02
**MFUC01**	4.89E-01	5.90E-01
**RBG-16-71-46_X_X_X**	8.66E-03	2.10E-02
**Mycobacteriaceae_X**	3.45E-03	8.80E-03
**Lacunisphaera**	6.39E-02	1.20E-01
**Glycomyces**	1.02E-02	2.40E-02
**Planococcaceae_X**	3.14E-01	4.40E-01
**UM-FILTER-40-16**	1.15E-04	4.00E-04
**2-12-FULL-35-15**	3.09E-01	4.30E-01
**Fen-1137**	5.03E-04	1.60E-03
**Algoriphagus**	4.47E-01	5.60E-01
**Chloroflexaceae_X**	6.73E-02	1.20E-01
**Roseomonas**	2.77E-02	5.80E-02
**UBA2475**	7.48E-01	8.00E-01
**Massilia**	1.75E-02	3.90E-02
**Fimbriimonadaceae_X**	5.35E-06	2.40E-05
**UTPRO1**	1.84E-05	7.50E-05
**Conexibacter_A**	2.40E-03	6.40E-03
**Ga0077550**	1.93E-09	1.60E-08
**Azoarcus_A**	3.78E-07	2.00E-06
**Bacillus_BE**	4.47E-01	5.60E-01
**Chamaesiphon**	8.56E-02	1.50E-01
**Paenibacillus_H**	4.12E-01	5.50E-01
**4572-78_X_X**	4.17E-02	8.20E-02
**Omnitrophales_X_X**	3.24E-05	1.30E-04

**Microbacterium**	1.97E-02	4.30E-02
**Myxococcota_X_X_X_X**	4.02E-05	1.50E-04
**GCA-2683135**	1.01E-08	7.00E-08
**Labrys**	4.32E-08	2.70E-07
**GCA-002840015**	7.25E-01	7.90E-01
**Chloroflexia_X_X_X**	5.36E-09	4.00E-08
**Cellulosimicrobium**	8.28E-02	1.50E-01
**HRBIN29**	4.11E-01	5.50E-01
**Pseudomonadales_X_X**	2.81E-05	1.10E-04
**Coriobacteriales_X_X**	3.92E-05	1.50E-04
**Palsa-1382**	2.31E-06	1.10E-05
**HRBIN33**	7.28E-11	8.30E-10
**Comamonas**	8.02E-01	8.40E-01
**Acidovorax_B**	3.91E-02	7.90E-02
**UTBCD1**	4.57E-03	1.10E-02
**GJ-E10**	1.36E-02	3.10E-02
**UBA12499**	1.04E-05	4.30E-05
**Symbiobacterium**	1.34E-01	2.20E-01
**Thermoactinospora**	2.98E-01	4.20E-01
**Cellulomonadaceae_X**	6.98E-02	1.30E-01
**Amantichitinum**	7.25E-01	7.90E-01
**Fimbriimonas**	2.36E-02	5.00E-02
**W-Firmicutes-14**	8.45E-01	8.70E-01
**Clostridiales_X_X**	3.63E-05	1.40E-04
**Stenotrophomonas_A**	2.57E-01	3.70E-01
**Cyclobacteriaceae_X**	5.75E-05	2.10E-04
**Afifella**	4.69E-02	9.10E-02
**UBA4722**	5.91E-06	2.50E-05
**Fuerstia**	4.39E-02	8.60E-02
**Azovibrio**	2.32E-01	3.50E-01
**Rubricoccus**	6.20E-02	1.20E-01
**PALSA-1448**	5.91E-02	1.10E-01
**Verrucomicrobiales_X_X**	1.31E-01	2.20E-01
**Abditibacterium**	1.69E-13	5.40E-12
**SZUA-309**	2.20E-01	3.30E-01
**Pseudonocardiaceae_X**	1.02E-01	1.80E-01
**Tistlia**	2.66E-05	1.10E-04
**HRBIN37**	6.13E-09	4.50E-08
**Ereboglobus**	2.44E-01	3.60E-01
**Nitrolancea**	7.26E-01	7.90E-01
**Phycisphaerales_X_X**	1.20E-03	3.40E-03
**Thermotalea**	1.46E-01	2.30E-01
**Bacillus_C**	5.15E-01	6.20E-01
**Roseiflexus**	9.75E-01	9.70E-01
**Marmoricola**	4.60E-02	9.00E-02
**Conexibacter**	1.80E-02	4.00E-02
**Rubrobacter**	5.69E-05	2.10E-04
**QHYU01**	1.26E-05	5.20E-05
**Mor1**	4.77E-02	9.20E-02
**UBA8087**	3.90E-02	7.90E-02
**Aquamicrobium_A**	2.30E-04	7.70E-04
**SG8-30**	2.03E-01	3.10E-01

**Saccharimonadia_X_X_X**	2.64E-06	1.20E-05
**Micrococcaceae_X**	1.97E-02	4.30E-02
**Hyphomicrobium**	7.08E-01	7.90E-01
**GR16-43**	6.61E-03	1.60E-02
**Kineosporia**	2.75E-03	7.20E-03
**Chloroploca**	1.36E-01	2.30E-01
**Fen-1247**	1.12E-01	1.90E-01
**Patescibacteria_X_X_X_X**	2.57E-01	3.70E-01
**Hymenobacteraceae_X**	2.80E-01	4.00E-01
**Bacilli_A_X_X_X**	3.70E-01	5.00E-01
**Arsukibacterium**	4.47E-01	5.60E-01
**Magnetospirillum_A**	4.89E-01	5.90E-01
**Tumebacillales_X_X**	7.65E-01	8.00E-01
**Vampirovibrionia_X_X_X**	2.58E-05	1.00E-04
**Methylocystis**	3.06E-02	6.30E-02
**Hydrogenedens**	2.15E-03	5.80E-03
**CG2-30-64-16**	5.71E-02	1.10E-01
**MED660**	4.41E-04	1.40E-03
**Vulgatibacter**	6.75E-04	2.10E-03
**Streptosporangiaceae_X**	4.04E-01	5.40E-01
**Bdellovibrio**	1.37E-02	3.10E-02
**Crossiella**	8.54E-04	2.50E-03
**Dermatophilaceae_X**	5.91E-05	2.20E-04
**2-12-FULL-57-22**	1.29E-01	2.20E-01
**Firmicutes_E_X_X_X_X**	8.95E-02	1.60E-01
**Alkaliphilus**	1.42E-01	2.30E-01
**ABY1_X_X_X**	2.93E-03	7.60E-03
**Methyloligella**	1.74E-01	2.70E-01
**Blastochloris**	8.25E-01	8.60E-01
**Kribbella**	4.06E-03	1.00E-02
**Rhodomicrobiaceae_X**	1.70E-01	2.70E-01
**Brevibacillus**	6.04E-01	7.20E-01
**Pseudomonas**	1.42E-01	2.30E-01
**Lysinibacillus_B**	7.53E-01	8.00E-01
**Bdellovibrionales_X_X**	5.63E-01	6.70E-01
**UBA3495_X**	2.67E-03	7.10E-03
**Ga0077541**	1.88E-04	6.40E-04
**TOLSYN**	1.25E-05	5.20E-05
**Hydrogenophaga**	1.21E-07	6.80E-07
**HZ-65**	9.69E-03	2.30E-02
**Crocinitomicaceae_X**	4.89E-01	5.90E-01
**Bacillus_AJ**	1.69E-03	4.60E-03
**GCA-2725915**	2.00E-04	6.70E-04
**Cohnella**	9.11E-01	9.20E-01
**Zambryskibacteraceae_X**	1.18E-04	4.10E-04
**Bacteroidota_X_X_X_X**	1.02E-05	4.20E-05
**Enterobacterales_X_X**	7.25E-01	7.90E-01
**PALSA-1337**	5.81E-04	1.80E-03
**SCGC-AG-212-F23**	6.96E-03	1.70E-02
**Bacillus_F**	2.57E-01	3.70E-01
**Mariniblastus**	2.22E-01	3.30E-01
**Pseudorhodoferax**	2.19E-03	5.90E-03

**JJ-A5**	2.19E-03	5.90E-03
**Elizabethkingia**	4.47E-01	5.60E-01
**Obscuribacter**	8.60E-01	8.80E-01
**Bacillus_AG**	6.40E-01	7.60E-01
**Sporomusaceae_X**	4.20E-01	5.60E-01
**Sphingobacteriaceae_X**	2.05E-02	4.40E-02
**Pajaroellobacter**	1.24E-01	2.10E-01
**Clostridium_AK**	1.97E-02	4.30E-02
**Zhizhongheella**	1.47E-02	3.40E-02
**Microlunatus**	4.42E-04	1.40E-03
**Mycobacterium_A**	1.12E-01	1.90E-01
**Bacillus**	4.16E-02	8.20E-02
**Pseudorhodoplanes**	2.87E-02	6.00E-02
**OLB9**	4.88E-01	5.90E-01
**UBA10212_X**	4.32E-08	2.70E-07
**Planctopirus**	5.09E-04	1.60E-03
**Moranbacterales_X_X**	2.16E-06	1.00E-05
**C7867-001**	8.40E-01	8.70E-01
**Bin61**	1.54E-02	3.50E-02
**4572-78**	9.67E-01	9.70E-01
**HRBIN40**	2.23E-05	9.00E-05
**AG11**	2.91E-01	4.20E-01
**Filimonas**	2.20E-02	4.70E-02
**Asinibacterium**	2.29E-03	6.10E-03
**Rummeliibacillus**	5.35E-01	6.40E-01
**OLB13**	5.50E-05	2.00E-04
**Gp7-AA6**	1.13E-01	1.90E-01
**Ga0074140_X**	4.02E-02	8.00E-02
**Desulfuromonadales_X_X**	8.56E-04	2.50E-03
**Exiguobacterium_A**	9.69E-03	2.30E-02
**Clostridium_AM**	2.14E-01	3.30E-01
**Ilumatobacter**	2.58E-01	3.70E-01
**UBA4735**	4.04E-02	8.00E-02
**Elusimicrobia_X_X_X**	5.45E-02	1.00E-01
**Enhygromyxa**	3.78E-02	7.70E-02
**Feb-22**	7.53E-01	8.00E-01
**Leptolyngbyaceae_X**	8.54E-04	2.50E-03
**Rhizobacter**	1.42E-01	2.30E-01
**UBA2337**	1.96E-01	3.00E-01
**Propionibacteriaceae_X**	8.54E-04	2.50E-03
**GCA-2737725**	9.69E-03	2.30E-02
**Paenibacillus_G**	4.89E-01	5.90E-01
**Piscinibacter**	3.84E-01	5.20E-01
**Methylobacterium**	4.56E-03	1.10E-02
**GWA1-54-10**	6.49E-01	7.60E-01
**Solirubrobacterales_X_X**	3.03E-02	6.30E-02
**Nitrosomonas**	4.85E-01	5.90E-01
**Pseudomonas_D**	4.47E-01	5.60E-01
**Flavobacteriaceae_X**	1.42E-01	2.30E-01
**Pararhizobium**	8.54E-04	2.50E-03
**UBA4416**	3.79E-01	5.20E-01
**Palsa-465**	3.79E-01	5.20E-01

**SG-1**	7.25E-01	7.90E-01
**Lachnospiraceae_X**	9.69E-03	2.30E-02
**Actinomycetales_X_X**	2.14E-01	3.30E-01
**UBA4207**	1.28E-01	2.20E-01
**Fen-455**	4.89E-01	5.90E-01
**Acetivibrio**	6.46E-01	7.60E-01
**Gp1-AA17**	2.59E-01	3.70E-01
**Paludisphaera**	9.48E-03	2.30E-02
**21-14-0-10-47-8**	3.10E-01	4.30E-01
**Limnobacter**	8.76E-01	8.90E-01
**UBA6065**	9.67E-01	9.70E-01
**UKL13-3**	2.15E-01	3.30E-01
**AG29**	4.89E-01	5.90E-01
**Paenibacillus_C**	4.47E-01	5.60E-01
**UBA2396**	1.37E-03	3.90E-03
**Myxococcales_X_X**	5.02E-01	6.10E-01
**Sulfurifustaceae_X**	1.67E-02	3.70E-02
**Azotobacter**	7.53E-01	8.00E-01
**UBA9983_A_X_X**	7.48E-02	1.40E-01
**UBA4124**	2.98E-01	4.20E-01
**Asticcacaulis**	1.00E-03	2.90E-03
**Rickettsiaceae_X**	6.28E-01	7.40E-01
**Paenibacillus_E**	8.49E-01	8.70E-01
**Klebsiella_B**	1.17E-01	2.00E-01
**Vitreoscilla_A**	9.83E-02	1.70E-01
**Paracraurococcus**	8.33E-06	3.50E-05
**20-14-0-80-50-8**	1.80E-05	7.40E-05
**SZUA-46**	7.53E-01	8.00E-01
**Diaphorobacter**	4.47E-01	5.60E-01
**Caulobacterales_X_X**	6.45E-01	7.60E-01
**Mongoliimonas**	2.57E-01	3.70E-01
**Gimesia**	1.90E-02	4.20E-02
**Streptomyces**	4.19E-01	5.60E-01
**Nitrospira_D**	2.22E-01	3.30E-01
**Palsa-1315**	4.89E-01	5.90E-01
**Dyella**	4.47E-01	5.60E-01
**Nannocystaceae_X**	1.35E-01	2.20E-01
**Palsa-1392**	1.06E-01	1.80E-01
**Jidaibacter**	1.97E-02	4.30E-02
**Bog-1198**	1.19E-01	2.00E-01
**Promicromonospora**	8.40E-01	8.70E-01
**Rhodothermales_X_X**	5.95E-02	1.10E-01
**Enterococcaceae_X**	4.47E-01	5.60E-01
**UBA10190**	2.45E-01	3.60E-01
**GCA-002862325**	2.69E-01	3.90E-01
**Pseudobacteroides**	8.54E-04	2.50E-03
**Porphyrobacter**	1.53E-01	2.40E-01
**Fulvimonas**	4.74E-02	9.20E-02
**Peptostreptococcales_X_X**	3.91E-02	7.90E-02
**Bacteriovoracaceae_X**	7.55E-03	1.80E-02
**GCA-2770975**	8.64E-03	2.10E-02
**Bin94**	1.54E-02	3.50E-02

**Rubritepida**	1.23E-02	2.80E-02
**Obscuribacteraceae_X**	7.96E-04	2.40E-03
**Paracaedibacter**	4.47E-01	5.60E-01
**Elainella**	6.15E-03	1.50E-02
**Geminicoccus**	4.21E-01	5.60E-01
**SG8-24_X**	4.89E-01	5.90E-01
**Paenibacillus**	7.55E-02	1.40E-01
**A4b_X**	6.10E-01	7.20E-01
**UBA1268**	4.89E-01	5.90E-01
**UBA12115**	1.40E-02	3.20E-02
**GCA-002050365**	2.28E-02	4.90E-02
**Ga0077555**	1.49E-01	2.40E-01
**2013-40CM-41-45**	3.15E-01	4.40E-01
**ZC4RG24**	3.03E-02	6.30E-02
**Parachlamydiales_X_X**	5.63E-01	6.70E-01
**GCA-2699025**	3.30E-01	4.60E-01
**Clostridium_T**	2.57E-01	3.70E-01
**Moheibacter**	3.91E-02	7.90E-02
**Cronobacter**	7.25E-01	7.90E-01
**Gorillibacterium**	1.02E-01	1.80E-01
**Pelomonas**	4.04E-01	5.40E-01
**Hydrocarboniphaga**	2.17E-01	3.30E-01
**Gemmatimonas**	1.66E-01	2.60E-01
**Bacillus_BC**	7.37E-01	8.00E-01
**UBA1018_X_X**	5.37E-02	1.00E-01
**Spirosomaceae_X**	2.57E-02	5.40E-02
**Tatlockia**	3.36E-01	4.60E-01
**Pigmentiphaga**	4.47E-01	5.60E-01
**Micavibrio_B**	8.83E-02	1.60E-01
**GAS474**	4.47E-01	5.60E-01
**Hyphomicrobiaceae_X**	7.73E-01	8.10E-01
**Enterococcus**	8.54E-04	2.50E-03
**Saccharopolyspora**	4.89E-01	5.90E-01
**Tol-SR**	3.49E-01	4.80E-01
**Anaerobacillus**	1.75E-01	2.80E-01
**Weeksellaceae_X**	2.57E-01	3.70E-01
**Chryseomicrobium**	7.55E-02	1.40E-01
**Niabella**	2.57E-01	3.70E-01
**Bdellovibrionota_X_X_X_X**	9.67E-01	9.70E-01
**O2-12-FULL-42-9**	2.98E-01	4.20E-01
**HO2-45-11b**	7.53E-01	8.00E-01
**XYD1-FULL-53-11**	2.32E-02	4.90E-02
**GWC2-37-73_X**	4.87E-02	9.30E-02
**Angustibacter**	7.25E-01	7.90E-01
**Babeliales_X_X**	2.38E-01	3.60E-01
**Actinoplanes**	7.36E-03	1.80E-02
**Amycolatopsis**	1.25E-01	2.10E-01
**2-01-FULL-59-12**	2.56E-01	3.70E-01
**Nostocaceae_X**	4.51E-01	5.60E-01
**F0540**	1.42E-01	2.30E-01
**Desulfonispora**	2.63E-02	5.50E-02
**2-12-FULL-44-17**	1.74E-01	2.70E-01

**SZUA-149**	8.90E-02	1.60E-01
**Bacillus_H**	7.25E-01	7.90E-01
**UBA10450_X**	7.10E-01	7.90E-01
**UBA920**	1.47E-01	2.40E-01
**Chitinibacteraceae_X**	7.25E-01	7.90E-01
**Xiphinematobacter**	7.53E-01	8.00E-01
**MPNL01**	7.52E-01	8.00E-01
**Sumerlaeaceae_X**	2.43E-07	1.30E-06
**Acidicaldus**	8.54E-04	2.50E-03
**1-14-0-10-56-10**	8.49E-01	8.70E-01
**PMMR1**	4.66E-03	1.20E-02
**Desulfitobacteriaceae_X**	7.25E-01	7.90E-01
**Herpetosiphonaceae_X**	1.33E-01	2.20E-01
**Mesorhizobium_A**	7.25E-01	7.90E-01
**Cesiribacter**	4.47E-01	5.60E-01
**OLB11**	1.42E-01	2.30E-01
**Uliginosibacterium**	1.38E-01	2.30E-01
**Propionibacteriales_X_X**	4.26E-02	8.30E-02
**Streptosporangium**	1.94E-01	3.00E-01
**Parachlamydiaceae_X**	3.04E-01	4.30E-01
**2-01-FULL-44-21**	9.87E-02	1.70E-01
**SZUA-47**	7.53E-01	8.00E-01
**UBA922_X**	7.53E-01	8.00E-01
**Sporichthya**	3.41E-01	4.70E-01
**Thermanaerothrix**	9.49E-02	1.70E-01
**Peribacteraceae_X**	1.07E-01	1.80E-01
**Ellin6529_X_X_X**	1.29E-01	2.20E-01
**UBA6164_X**	7.53E-01	8.00E-01
**AKYH767_X_X**	4.26E-02	8.30E-02
**Taibaiella_B**	8.63E-01	8.80E-01
**32-67-11**	7.25E-01	7.90E-01
**Enterobacter_B**	2.57E-01	3.70E-01
**Paeniclostridium**	7.53E-01	8.00E-01
**UBA6821**	4.47E-01	5.60E-01
**Mycolicibacillus**	4.89E-01	5.90E-01
**Legionellaceae_X**	2.15E-01	3.30E-01
**Methyloceanibacter**	1.15E-01	2.00E-01
**2-02-FULL-42-43**	2.51E-01	3.70E-01
**2-02-FULL-39-32**	4.89E-01	5.90E-01
**Koribacter**	9.67E-01	9.70E-01
**Kapabacteriales_X_X**	3.67E-01	5.00E-01
**Rickettsiella**	7.25E-01	7.90E-01
**Pseudescherichia**	2.57E-01	3.70E-01
**UBA9655**	7.10E-01	7.90E-01
**Geobacter_B**	4.47E-01	5.60E-01
**UBA9649**	2.00E-01	3.10E-01
**Lysinibacillus**	1.04E-01	1.80E-01
**Ga0074137**	5.46E-01	6.50E-01
**UBA9934**	8.25E-01	8.60E-01
**Melittangium**	2.98E-01	4.20E-01
**Rudanella**	1.23E-02	2.80E-02
**Solimonas**	7.53E-01	8.00E-01

**Comamonas_E**	7.25E-01	7.90E-01
**Microtrichales_X_X**	1.40E-01	2.30E-01
**Sporomusa**	4.16E-02	8.20E-02
**Risungbinella**	7.07E-02	1.30E-01
**PALSA-555**	7.53E-01	8.00E-01
**Eubacterium_F**	7.25E-01	7.90E-01
**UBA11600**	7.57E-01	8.00E-01
**Acidisphaera**	3.32E-01	4.60E-01
**UBA9628**	5.56E-02	1.00E-01
**DDH964**	9.67E-01	9.70E-01
**Stackebrandtia**	8.54E-04	2.50E-03
**Methylacidiphilales_X_X**	1.71E-02	3.80E-02
**UBA11359_X**	2.54E-01	3.70E-01
**Brevibacillus_B**	7.53E-01	8.00E-01
**UBA12053**	9.67E-01	9.70E-01
**Oligoflexus**	6.48E-01	7.60E-01
**Phreatobacter**	3.91E-02	7.90E-02
**SG8-24_X_X**	9.65E-05	3.40E-04
**2-02-FULL-39-32_X_X**	2.98E-01	4.20E-01
**Altererythrobacter_B**	3.94E-01	5.30E-01
**Turicibacter**	7.25E-01	7.90E-01
**Actinotalea**	7.25E-01	7.90E-01
**UBA11704**	4.47E-01	5.60E-01
**Thermoanaerobaculia_X_X_X**	6.48E-01	7.60E-01
**SZUA-55**	1.85E-01	2.90E-01
**QKMZ01**	9.12E-01	9.20E-01
**Nucleicultrix**	7.25E-01	7.90E-01
**Sphaerospermopsis_A**	5.00E-02	9.50E-02
**Faecalicatena**	7.53E-01	8.00E-01
**Polyangia_X_X_X**	4.15E-01	5.60E-01
**Bacillus_L**	4.47E-01	5.60E-01
**OLB15**	3.54E-03	9.10E-03
**01-FULL-45-10b**	8.46E-01	8.70E-01
**UBA1931**	7.55E-02	1.40E-01
**GCA-2699585**	3.27E-03	8.40E-03
**UBA1547**	2.57E-01	3.70E-01
**2-12-FULL-60-19**	8.54E-04	2.50E-03
**Aquimonas**	7.25E-01	7.90E-01
**Bacillus_AX**	4.47E-01	5.60E-01
**Khelaifiella**	7.25E-01	7.90E-01
**OLB5**	7.58E-02	1.40E-01
**OLB20_X**	7.69E-01	8.10E-01
**OLB23**	4.89E-01	5.90E-01
**GCF-001939115**	4.26E-02	8.30E-02
**Paenibacillus_T**	7.25E-01	7.90E-01
**Vermiphilus**	7.55E-02	1.40E-01
**Nevskiales_X_X**	4.89E-01	5.90E-01
**HR-BB**	1.15E-01	2.00E-01
**Cyanobacteria_X_X_X_X**	8.39E-02	1.50E-01
**Tumebacillus**	2.98E-01	4.20E-01
**HdN1**	8.46E-01	8.70E-01
**2-02-FULL-49-12**	7.25E-01	7.90E-01

**Bacillus_Q**	7.25E-01	7.90E-01
**Sphingomonas**	7.25E-01	7.90E-01
**BOG-1112**	2.10E-01	3.20E-01
**UBA9973_X**	7.53E-01	8.00E-01
**SZUA-521**	7.72E-01	8.10E-01
**UBA9631**	2.68E-01	3.80E-01
**Algiphilus**	7.55E-02	1.40E-01
**Gastranaerophilales_X_X**	1.42E-01	2.30E-01
**UBA1844**	8.76E-01	8.90E-01
**Larkinella**	2.02E-01	3.10E-01
**Bdellovibrionaceae_X**	4.16E-02	8.20E-02
**UBA4658**	7.63E-01	8.00E-01
**PALSA-1347**	7.25E-01	7.90E-01
**Deinococcaceae_X**	7.53E-01	8.00E-01
**Serratia**	7.25E-01	7.90E-01
**Aquincola**	7.25E-01	7.90E-01
**Alsobacter**	7.46E-07	3.80E-06
**Chondromyces**	4.24E-01	5.60E-01
**UTPRO2**	8.68E-01	8.90E-01
**Bacillus_AT**	2.98E-01	4.20E-01
**UBA11063**	4.47E-01	5.60E-01
**Ancylobacter**	7.25E-01	7.90E-01
**GCA-2707205**	4.47E-01	5.60E-01
**Deinococcus_C**	7.25E-01	7.90E-01
**SZUA-359**	7.25E-01	7.90E-01
**RBG-16-40-8**	4.43E-01	5.60E-01
**Deinococcales_X_X**	7.53E-01	8.00E-01
**Paracaedibacteraceae_X**	6.20E-01	7.30E-01
**Thermoactinomycetaceae_X**	9.12E-01	9.20E-01
**Zoogloea**	4.47E-01	5.60E-01
**Paenibacillus_N**	7.25E-01	7.90E-01
**Oscillatoria**	5.00E-02	9.50E-02
**Segetibacter**	4.89E-01	5.90E-01
**Flavobacteriales_X_X**	5.99E-01	7.10E-01
**Ochrobactrum_A**	7.25E-01	7.90E-01
**Tistrella**	4.89E-01	5.90E-01
**BAL199**	4.89E-01	5.90E-01
**UBA1400_X_X**	7.53E-01	8.00E-01
**UBA1565_X**	9.87E-02	1.70E-01
**UBA920_X_X**	8.60E-01	8.80E-01
**Shewanella**	7.25E-01	7.90E-01
**Chelatococcus**	7.25E-01	7.90E-01
**Pedobacter**	8.31E-01	8.60E-01
**UBA1565_X_X**	9.67E-01	9.70E-01
**UBA1532**	2.98E-01	4.20E-01
**XYD2-FULL-39-9**	7.53E-01	8.00E-01
**UBA10212**	7.53E-01	8.00E-01
**Legionella_A**	1.42E-01	2.30E-01
**UBA11426**	7.53E-01	8.00E-01
**Aquabacterium**	4.47E-01	5.60E-01
**WO2-54-10**	7.53E-01	8.00E-01
**Terribacillus**	8.02E-01	8.40E-01

**Dehalobacterium**	2.98E-01	4.20E-01
**Vogesella**	7.25E-01	7.90E-01
**UBA1573**	4.47E-01	5.60E-01
**UBA6139**	4.89E-01	5.90E-01
**UBA1369_X_X**	4.47E-01	5.60E-01
**Dietzia**	7.25E-01	7.90E-01
**Bacteroidales_X_X**	4.47E-01	5.60E-01
**Thiobacillus**	7.25E-01	7.90E-01
**Amphiplicatus**	8.54E-04	2.50E-03
**Desulfovibrionales_X_X**	4.16E-02	8.20E-02
**Amphibacillaceae_X**	8.54E-04	2.50E-03
**Brevibacillaceae_X**	8.54E-04	2.50E-03
**Paenibacillus_L**	2.39E-03	6.40E-03
**GWA2-52-8**	5.00E-02	9.50E-02
**UBA10190_X**	1.48E-01	2.40E-01
**Elusimicrobiota_X_X_X_X**	7.53E-01	8.00E-01
**SZUA-592**	9.67E-01	9.70E-01
**Micavibrio**	8.60E-01	8.80E-01
**Odyssella**	7.25E-01	7.90E-01
**01-FULL-45-34b**	7.25E-01	7.90E-01
**Rubinisphaera**	9.67E-01	9.70E-01
**Simkania**	7.25E-01	7.90E-01
**Gracilibacteria_X_X_X**	7.25E-01	7.90E-01
**Sphingomonas_D**	7.25E-01	7.90E-01
**DSM-45891**	7.25E-01	7.90E-01
**Gracilibacillus**	8.76E-01	8.90E-01
**Siphonobacter**	2.57E-01	3.70E-01
**Caloramatoraceae_X**	4.47E-01	5.60E-01
**1NLA3E**	7.53E-01	8.00E-01
**Longispora**	7.25E-01	7.90E-01
**Opitutales_X_X**	7.25E-01	7.90E-01
**Salinimicrobium**	4.26E-02	8.30E-02
**Leptolyngbya_A**	2.03E-01	3.10E-01
**Xanthomonas_A**	7.25E-01	7.90E-01
**Deinococcus**	4.47E-01	5.60E-01
**VKM-B-2647**	9.67E-01	9.70E-01
**21-14-0-10-47-8-A**	1.42E-01	2.30E-01
**Bdellovibrio_A**	7.25E-01	7.90E-01
**Ruminococcus_A**	4.47E-01	5.60E-01
**Desulfotomaculum_H**	3.84E-01	5.20E-01
**UBA2163_X**	2.47E-01	3.70E-01
**Ktedonobacter**	8.54E-04	2.50E-03
**2-02-FULL-51-10**	2.98E-01	4.20E-01
**OLB19**	1.42E-01	2.30E-01
**28-YEA-48**	7.25E-01	7.90E-01
**Palsa-1150**	7.25E-01	7.90E-01
**Enterococcus_A**	7.25E-01	7.90E-01
**OLB8**	4.47E-01	5.60E-01
**Kroppenstedtia**	1.07E-01	1.80E-01
**Ketobacteraceae_X**	4.87E-02	9.30E-02
**Elainellaceae_X**	7.53E-01	8.00E-01
**Anaerocolumna**	7.25E-01	7.90E-01

**GCA-2686445**	4.47E-01	5.60E-01
**Bacteriovorax**	4.47E-01	5.60E-01
**Solirubrum**	4.47E-01	5.60E-01
**HO2-37-13b_X**	9.67E-01	9.70E-01
**Protochlamydia**	4.47E-01	5.60E-01
**Desulfobacterota_X_X_X_X**	8.49E-01	8.70E-01
**Diplorickettsia**	4.89E-01	5.90E-01
**Magnetospirillaceae_X**	4.89E-01	5.90E-01
**PALSA-968**	4.47E-01	5.60E-01
**GWA1-44-29**	4.89E-01	5.90E-01
**UBA10103**	4.89E-01	5.90E-01

Table S3: Wilcoxon test comparison p-value of all genera between all pairs of four sample types. "_X" are used to represent unannotated taxa at a level, multiple suffixes indicate that many level was the last annotation. P-values < 0.05 are coloured as red.

	Bulk			PostHarvest		PreSowing
**Genera**	PostHarvest	PreSowing	Rhizosphere	PreSowing	Rhizosphere	Rhizosphere
**Pseudomonas_M**	0.016	0.527	0.000	0.222	0.593	0.004
**Acinetobacter**	0.969	0.875	0.000	0.906	0.004	0.003
**Enterobacter_D**	0.957	0.612	0.000	1.000	0.000	0.000
**Bacteria_X_X_X_X_X**	0.280	0.000	0.000	0.008	0.000	0.000
**Bacillus_W**	0.820	0.793	0.000	1.000	0.013	0.011
**Bacillus_AK**	0.741	0.495	0.000	1.000	0.002	0.002
**UBA2421**	0.042	0.930	0.003	0.056	0.672	0.149
**Bacillaceae_A_X**	0.154	0.080	0.000	0.548	0.021	0.029
**Luteitalea**	0.687	0.000	0.000	0.008	0.000	0.495
**Enterobacteriaceae_X**	0.196	0.649	0.000	0.424	0.001	0.000
**Pseudomonadaceae_X**	0.611	0.023	0.000	0.032	0.000	0.059
**Sphingomonadaceae_X**	0.001	0.007	0.000	0.151	0.000	0.000
**Lysobacter**	0.678	0.338	0.000	0.841	0.000	0.000
**Rhizobiaceae_X**	0.314	0.875	0.000	0.421	0.001	0.000
**QHWT01**	0.128	0.051	0.000	0.032	0.000	0.002
**Streptomycetaceae_X**	0.086	0.080	0.287	0.841	0.160	0.138
**Bacillales_X_X**	0.280	0.128	0.000	0.548	0.101	0.326
**OLB17**	0.020	0.170	0.010	0.421	0.004	0.008
**GWA2-73-35_X**	0.035	0.006	0.000	0.008	0.000	0.000
**Actinobacteriota_X_X_X_X**	0.018	0.000	0.000	0.095	0.000	0.000
**Peptostreptococcaceae_X**	0.375	0.123	0.000	0.180	0.003	0.011
**Palsa-739**	0.118	0.000	0.000	0.032	0.000	0.000
**Chthoniobacter**	0.110	0.148	0.000	0.841	0.149	0.109
**Opitutaceae_X**	0.011	0.144	0.776	0.548	0.001	0.040
**QHXM01**	0.137	0.102	0.000	0.151	0.000	0.001
**Alphaproteobacteria_X_X_X**	0.073	0.042	0.000	0.008	0.010	0.000
**Flavobacterium**	0.330	0.978	0.000	0.424	0.002	0.004
**Haloferula**	0.273	0.865	0.000	0.347	0.001	0.002
**UBA9968_X**	0.986	0.000	0.000	0.008	0.000	0.755
**Burkholderiales_X_X**	0.194	0.000	0.000	0.008	0.000	0.005
**Micromonosporaceae_X**	0.314	0.056	0.000	0.151	0.049	0.001
**Pseudosphingobacterium**			0.000		0.034	0.034
**Xylophilus**	0.023	0.930	0.000	0.056	0.000	0.071
**UBA11741**	0.067	0.016	0.000	0.016	0.000	0.002
**Sphningosinicella**	0.000	0.349	0.000	0.016	0.000	0.172
**Xanthomonadaceae_X**	0.249	0.028	0.000	0.032	0.345	0.000
**Verrucomicrobium**	0.980	0.901	0.000	1.000	0.001	0.001
**Microvirga**	0.349	0.002	0.207	0.016	0.149	0.007
**Chryseobacterium**	0.777	0.777	0.000		0.001	0.001
**Acidimicrobiia_X_X_X**	0.611	0.004	0.000	0.032	0.016	0.000
**Bacillus_Y**	0.330	0.301	0.000	0.180	0.003	0.010
**Burkholderiaceae_X**	0.958	0.687	0.000	0.690	0.006	0.016
**Pirellula**	0.636	0.562	0.000	0.690	0.000	0.008
**Gp6-AA56**	0.170	0.002	0.000	0.095	0.000	0.092
**Steroidobacter**	0.170	0.331	0.001	0.095	0.619	0.016
**Vitiosangium**	0.986	0.026	0.000	0.151	0.005	0.001
**Planctomycetaceae_X**	0.102	0.249	0.000	0.056	0.519	0.007

Vicinamibacterales_X_X	0.087	0.000	0.000	0.008	0.000	0.071
Fimbriiglobus	0.586	0.875	0.000	0.690	0.001	0.002
Saccharimonadaceae_X	0.643	0.751	0.000	0.526	0.035	0.014
Sphingopyxis_A	0.849	0.451	0.000	0.441	0.000	0.000
UBA6082	0.042	0.118	0.000	0.841	0.000	0.000
Pontibacter	0.038	0.504	0.000	0.690	0.000	0.000
GCA-2746885	0.748	0.968	0.000	0.906	0.008	0.013
Sphingomonas_A	0.000	0.000	0.105	0.548	0.000	0.000
Clostridiaceae_X	0.050	0.567	0.000	0.530	0.262	0.311
Nonomuraea	0.011	0.187	0.034	0.151	0.001	0.005
Pirellulales_X_X	0.118	0.611	0.000	0.310	0.000	0.000
AR5	0.539	0.001	0.000	0.016	0.000	0.172
Neorhizobium	0.427	0.691	0.000	0.424	0.000	0.000
Pseudoxanthomonas_A	0.262	0.432	0.000	0.675	0.167	0.000
Clostridium	0.649	0.196	0.000	0.424	0.009	0.032
Gemmatimonadaceae_X	0.661	0.207	0.000	0.151	0.000	0.000
GWC2-73-18	0.847	0.005	0.000	0.008	0.002	0.000
Ohtaekwangia	0.061	0.515	0.015	0.548	0.543	0.405
UKL13-2	1.000	0.264	0.024	0.421	0.257	0.092
Geodermatophilus	0.871	0.004	0.079	0.032	0.092	0.002
FW-11	0.139	0.783	0.000	0.373	0.001	0.011
Dongia	1.000	0.249	0.000	0.421	0.005	0.257
JKG1	0.108	0.013	0.037	0.095	0.032	0.001
Pirellulaceae_X	0.011	0.636	0.528	0.008	0.032	0.783
Arboricoccus	0.636	0.013	0.000	0.056	0.000	0.000
Mycoplana	0.766	1.000	0.000	1.000	0.004	0.001
Flavisolibacter	0.020	0.158	0.000	0.548	0.000	0.000
Leaf454	0.588	0.588	0.000	1.000	0.026	0.039
Sphingobacterium			0.000		0.004	0.004
Gemmata	0.001	0.739	0.000	0.056	0.000	0.002
Pedosphaerales_X_X	0.234	0.713	0.130	0.421	0.149	0.308
Clostridium_AD			0.000		0.050	0.050
Actinobacteria_X_X_X	0.067	0.004	0.000	0.310	0.000	0.000
Alteromonadaceae_X			0.019		0.414	0.414
Adhaeribacter	0.329	0.199	0.007	0.290	0.007	0.820
Nannocystis	0.494	0.698	0.852	0.607	0.555	0.607
QHVH01	0.793	0.001	0.000	0.056	0.000	0.005
Bryobacteraceae_X	0.249	0.137	0.000	0.548	0.000	0.000
Pedosphaeraceae_X	0.280	0.073	0.046	0.421	0.134	0.085
Rhodocyclaceae_X	0.067	0.035	0.000	0.690	0.007	0.040
UBA1161_X	0.713	0.042	0.116	0.690	0.927	0.385
Geminicoccaceae_X	0.128	0.011	0.000	0.151	0.000	0.000
SCN-69-37	0.047	0.073	0.000	0.032	0.000	0.000
Lysobacter_A	0.019	0.616	0.000	0.072	0.000	0.000
Solirubrobacter	0.067	0.000	0.000	0.016	0.000	0.000
AR19	0.958	0.005	0.000	0.032	0.000	0.012
Aromatoleum	0.038	0.704	0.046	0.095	0.006	0.761
Gemmataceae_X	0.006	0.847	0.000	0.056	0.000	0.002
UBA4664	0.375	0.797	0.000	0.424	0.023	0.060
Pedosphaera	0.314	0.006	0.000	0.032	0.008	0.004
Planobispora	0.265	0.130	0.864	0.106	0.177	0.147
Cellvibrio	0.559	0.559	0.000		0.003	0.003

Chitinophagaceae_X	0.249	0.428	0.000	0.151	0.003	0.149
SZUA-115	0.280	0.003	0.000	0.008	0.003	0.000
Planctomycetes_X_X_X	0.249	0.820	0.000	0.548	0.000	0.000
PALSA-1355	0.687	0.042	0.000	0.151	0.000	0.001
Stenotrophomonas	0.649	0.649	0.000		0.001	0.001
Gp7-AA10	0.148	0.000	0.000	0.095	0.023	0.927
Solirubrobacteraceae_X	0.047	0.000	0.000	0.032	0.000	0.000
Bacilli_X_X_X	0.706	0.936	0.000	1.000	0.064	0.042
ARS98	0.009	0.137	0.000	0.310	0.000	0.000
Rubrobacter_B	0.687	0.102	0.000	0.095	0.001	0.001
Bacillus_BD	0.676	0.839	0.001	0.797	0.227	0.124
Thauera	0.138	0.171	0.047	0.072	0.536	0.042
Catelliglobosispora	0.388	0.745	0.000	1.000	0.218	0.149
Steroidobacteraceae_X	0.137	0.006	0.000	0.690	0.956	0.543
Gemmatimonadales_X_X	0.000	0.023	0.000	0.095	0.118	0.519
Kosakonia			0.010		0.362	0.362
Cupriavidus	0.375	0.001	0.000	0.025	0.000	0.014
Oscillochloris	0.257	0.115	0.169	0.917	0.607	0.165
UBA4665_X	0.129	0.129	0.000		0.001	0.001
Pyrinomonadaceae_X	0.158	0.000	0.000	0.095	0.000	0.021
RBG-16-71-46	0.220	0.056	0.000	0.056	0.001	0.006
Opitutus	0.029	0.086	0.000	1.000	0.003	0.005
Thermomicrobiaceae_X	0.626	0.005	0.000	0.095	0.008	0.001
2-12-FULL-64-23	0.018	0.539	0.000	0.310	0.000	0.029
Chitinophaga	0.148	0.028	0.000	0.833	0.460	0.021
Isosphaeraceae_X	0.611	0.000	0.000	0.032	0.035	0.000
Myxococcus	0.597	0.012	0.817	0.067	0.498	0.016
Niastella	0.149	0.741	0.001	0.421	0.646	0.240
Mycobacteriales_X_X	0.626	0.020	0.000	0.151	0.007	0.004
Gammaproteobacteria_X_X_X	0.820	0.000	0.000	0.032	0.211	0.059
Polyangiales_X_X	0.176	0.009	0.003	0.008	0.676	0.007
UBA10511	0.713	0.515	0.000	0.841	0.044	0.211
Plantactinospora_A	0.928	0.004	0.000	0.056	0.007	0.001
Nitrospira_C	0.002	0.000	0.000	0.008	0.044	0.040
Thermoleophilia_X_X_X	0.018	0.000	0.000	0.548	0.000	0.000
Phenylobacterium	0.011	0.713	0.015	0.095	0.000	0.149
Bacillus_AA	0.603	0.691	0.000	1.000	0.018	0.014
Methylibium	0.086	0.015	0.001	0.310	0.727	0.241
AV2	0.000	0.875	0.000	0.008	0.000	0.001
40CM-3-62-11	0.148	0.000	0.000	0.008	0.000	0.790
Pararheinheimera	0.330	0.935	0.969	0.424	0.362	0.930
BOG-1338	0.980	0.747	0.000	1.000	0.020	0.013
Exiguobacterium			0.000		0.042	0.042
Methylophilaceae_X	0.777	0.777	0.000		0.002	0.002
Acidobacteriota_X_X_X_X	0.176	0.018	0.000	0.421	0.001	0.015
Gemmatirosa	0.003	0.428	0.059	0.095	0.026	0.956
SCN-70-22	0.428	0.023	0.000	0.056	0.000	0.002
Rhizobium		0.006	0.000	0.424	0.000	0.001
Erwinia	0.777	0.777	0.006		0.241	0.241
Nocardioidaceae_X	0.455	0.022	0.050	0.106	0.074	0.044
Arthrobacter_I	0.211	0.116	0.000	0.158	0.001	0.583
CP2B	0.487	0.487	0.001		0.099	0.099

**Achromobacter**			0.000		0.009	0.009
**Flavobacterium_A**			0.005		0.316	0.316
**BOG-1460**	0.181	0.958	0.000	0.690	0.000	0.001
**40CM-68-15**	0.240	0.226	0.000	0.059	0.000	0.030
**Pseudoxanthomonas**	0.777	0.777	0.001		0.182	0.182
**Rubrobacteraceae_X**	0.759	0.143	0.000	0.222	0.001	0.001
**Zavarzinella**	0.471	0.264	0.064	0.421	0.925	0.039
**SM1A02_X**	0.039	0.713	0.000	0.421	0.000	0.002
**Paenibacillaceae_X**	0.124	0.408	0.053	1.000	0.803	0.576
**Byssovorax**	0.181	0.220	0.000	1.000	0.000	0.000
**Haliangium**	0.342	0.028	0.009	0.398	0.003	0.000
**Dactylosporangium**	0.220	0.104	0.000	0.151	0.016	0.026
**Inquilinus**	0.835	0.140	0.000	0.347	0.004	0.099
**Paramesorhizobium**			0.002		0.276	0.276
**Aeromonas**			0.000		0.136	0.136
**Terrimicrobium**	0.034	0.361	0.000	0.607	0.012	0.008
**Schlesneria**	0.110	0.181	0.000	0.008	0.000	0.000
**G233**	0.004	0.045	0.000	1.000	0.878	0.939
**Acidimicrobiales_X_X**	0.678	0.024	0.948	0.056	0.718	0.042
**Bacteroidia_X_X_X**	0.871	0.053	0.027	0.095	0.145	0.557
**Streptacidiphilus**	0.109	0.473	0.395	0.824	0.024	0.209
**Nevskiaceae_X**	0.376	0.074	0.000	0.421	0.023	0.011
**UBA6960_X**	0.001	0.000	0.000	0.008	0.000	0.238
**UBA2386**	0.471	0.220	0.000	0.548	0.004	0.118
**SZUA-42**	0.303	0.092	0.000	0.032	0.000	0.139
**Kouleothrix**	0.958	0.031	0.000	0.151	0.020	0.002
**SZUA-252**	1.000	0.006	0.000	0.032	0.000	0.085
**Pirellula_B**	0.070	0.335	0.001	0.161	0.001	0.910
**Jiangella**	0.006	0.000	0.000	0.690	0.000	0.000
**Bin18**	0.586	0.102	0.000	0.421	0.040	0.211
**Saprospiraceae_X**	0.058	0.705	0.030	0.151	0.006	0.495
**Palsa-1233**	0.611	0.449	0.001	0.690	0.138	0.241
**Phycisphaerae_X_X_X**	0.611	0.249	0.000	0.548	0.001	0.000
**Ilumatobacter_A**	0.039	0.031	0.006	0.056	0.543	0.006
**UBA11883**	0.875	0.005	0.000	0.008	0.000	0.138
**Dehalococcoidia_X_X_X**	0.793	0.000	0.000	0.010	0.002	0.067
**Rhizobiales_X_X**	0.181	0.003	0.925	0.095	0.364	0.016
**Polyangiaceae_X**	0.739	0.449	0.000	0.310	0.000	0.001
**UBA5946**			0.000		0.002	0.002
**Cyanobacteriia_X_X_X**	0.610	0.076	0.025	0.389	0.043	0.011
**Bordetella_A**			0.000		0.023	0.023
**Bradyrhizobium**			0.000		0.136	0.136
**Fictibacillus_B**			0.000		0.034	0.034
**Noviherbaspirillum**	0.305	0.299	0.000	0.158	0.003	0.204
**Citrobacter**			0.317		0.772	0.772
**Chthoniobacterales_X_X**	0.587	0.433	0.000	1.000	0.004	0.005
**Chitinophagales_X_X**	0.199	0.625	0.000	0.222	0.000	0.029
**PMNU01**	0.121	0.170	0.115	1.000	0.425	0.423
**QHBO01**	0.428	0.002	0.000	0.151	0.002	0.000
**P52-10**	0.449	0.170	0.006	0.151	0.029	0.985
**Burkholderia**			0.000		0.071	0.071
**Azospirillum**	0.501	0.001	0.188	0.034	0.425	0.003

**Alishewanella**	0.330	0.438	0.908	0.180	0.362	0.357
**Gemmatimonadetes_X_X_X**	0.068	0.053	0.000	1.000	0.011	0.037
**Novosphingobium**			0.149		0.640	0.640
**Dyadobacter**	0.777	0.077	0.000	0.424	0.000	0.006
**UBA1020**	0.649	0.649	0.000		0.028	0.028
**Acidobacteriales_X_X**	0.652	0.134	0.000	0.222	0.001	0.134
**MM2**	0.559	0.559	0.000		0.000	0.000
**SWB02**	0.428	0.428	0.000	1.000	0.001	0.001
**Geodermatophilaceae_X**	0.226	0.017	0.066	0.045	0.649	0.003
**Bin134**	0.530	0.000	0.144	0.012	0.985	0.001
**Saccharimonadales_X_X**	0.086	0.329	0.000	0.034	0.003	0.018
**Domibacillaceae_X**	0.363	0.312	0.635	0.236	0.437	0.280
**Roseimaritima**	0.244	0.886	0.284	0.607	0.035	0.387
**Aeromicrobium**	0.162	0.003	0.020	0.290	0.984	0.263
**Saccharibacillus**			0.317		0.772	0.772
**UBA4655**	0.011	0.376	0.000	0.142	0.000	0.223
**Coleofasciculus**	0.978	0.014	0.302	0.158	0.518	0.002
**Blastomonas**	0.649	0.008	0.000	0.180	0.006	0.146
**Palsa-89**	0.407	0.042	0.157	0.421	0.747	0.103
**Chloroflexota_X_X_X_X**	0.005	0.611	0.010	0.008	0.032	0.016
**Polymorphum**	0.413	0.818	0.702	0.373	0.420	0.677
**Blastococcus**	0.241	0.025	0.089	0.075	0.381	0.013
**SZUA-320**	0.116	0.303	0.000	0.095	0.000	0.018
**Nitrospira_A**	0.006	0.000	0.000	0.548	0.719	0.663
**Rhodobacteraceae_X**	0.232	0.447	0.538	0.173	0.032	0.297
**IMCC26207**	0.319	0.019	0.000	0.548	0.009	0.000
**55-13**	0.212	0.073	0.001	0.008	0.155	0.000
**GCA-2723275**	0.240	0.011	0.000	0.008	0.000	0.437
**LHW63021**	0.668	0.175	0.669	0.347	0.829	0.072
**Nocardioides**	0.142	0.000	0.033	0.032	0.609	0.001
**Isoptericola_B**	0.323	0.002	0.008	0.142	0.893	0.041
**ZC4RG30**	0.713	0.515	0.000	0.548	0.026	0.042
**Cyanobacteriales_X_X**	0.644	0.184	0.009	0.239	0.475	0.002
**Terrimonas**	0.012	0.649	0.000	0.180	0.784	0.085
**Planctomicrobium**	0.986	0.148	0.000	0.310	0.000	0.005
**Hyalangium**	0.823	0.214	0.001	0.265	0.471	0.068
**Acetobacteraceae_X**	0.056	0.062	0.000	0.067	0.001	0.405
**Reyranella**	0.666	0.639	0.238	1.000	0.269	0.269
**Arenimonas**	0.649	0.260	0.000	0.424	0.004	0.012
**Chthonomonas**	0.240	0.871	0.000	0.095	0.000	0.000
**Pseudomonas_A**	0.559	0.559	0.000		0.042	0.042
**Taibaiella_A**			0.000		0.099	0.099
**Azohydromonas**	0.941	0.002	0.001	0.075	0.140	0.066
**Sphingopyxis**			0.000		0.042	0.042
**Verrucomicrobiae_X_X_X**	0.416	0.759	0.278	0.310	1.000	0.394
**Rhizorhabdus**	0.042	0.095	0.431	1.000	0.049	0.059
**Devosia**	0.501	0.011	0.123	0.056	0.834	0.216
**Cryptosporangium**	0.441	0.000	0.930	0.010	0.490	0.000
**Chryseolinea_A**			0.000		0.071	0.071
**Prosthecobacter**	0.143	0.668	0.000	0.398	0.029	0.009
**Rhodospirillum_A**	0.883	0.102	0.100	0.265	0.271	0.017
**SZUA-318**	0.031	0.008	0.000	0.310	0.000	0.000

Pseudomonas_F	0.487	0.487	0.000		0.042	0.042
Rubellimicrobium	0.492	0.003	0.009	0.032	0.109	0.042
Sorangium	0.540	0.041	0.000	0.067	0.481	0.003
GCA-002686595_X	0.074	0.148	0.000	0.032	0.000	0.102
Planctomyces_A	0.385	0.015	0.000	0.034	0.006	0.955
Pseudomonas_B	0.649	0.649	0.579		0.772	0.772
Chryseolinea	0.095	0.095	0.113	1.000	0.245	0.245
Bosea	0.777	0.777	0.000		0.007	0.007
Blastocatellia_X_X_X	0.011	0.376	0.000	0.290	0.125	0.405
Azoarcus_B	0.099	0.160	0.000	0.106	0.002	0.601
Herpetosiphon	0.797	0.151	0.000	0.607	0.065	0.254
NIC37A-2	0.213	0.957	0.000	0.222	0.000	0.001
Rhodanobacteraceae_X	0.839	0.200	0.000	0.265	0.092	0.003
Pseudonocardia	0.164	0.026	0.001	0.310	0.012	0.004
KBS-96	0.286	0.045	0.000	0.222	0.008	0.003
Xanthobacteraceae_X	0.759	0.154	0.940	0.310	0.811	0.172
GWC2-71-9_X	0.108	0.426	0.001	0.075	0.000	0.593
Cystobacter	0.375	0.151	0.149	0.180	0.158	0.375
NS-102	0.839	0.619	0.244	0.833	0.830	0.276
Pseudomonas_H			0.074		0.548	0.548
Flavisolibacter_B	0.224	0.649	0.000	0.424	0.002	0.003
GWC2-70-10	0.097	0.003	0.000	0.067	0.004	0.570
Aneurinibacillus_A	0.786	0.320	0.010	0.530	0.573	0.024
QKVK01	0.034	0.493	0.056	0.398	0.133	0.801
Anaeromyxobacter	0.159	0.596	0.455	0.753	0.105	0.355
67-14	0.181	0.089	0.001	0.045	0.936	0.001
Streptomyces_D	0.291	0.897	0.015	0.424	0.772	0.087
HRBIN30	0.504	0.871	0.000	0.548	0.001	0.005
Par-f-2	0.899	0.576	0.000	0.690	0.010	0.001
Myxococcaceae_X	0.985	0.021	0.051	0.139	0.347	0.051
UBA5793	0.626	0.154	0.000	0.142	0.000	0.175
Z2-YC6860	0.246	0.985	0.002	0.236	0.762	0.050
Proteobacteria_X_X_X_X	0.192	0.926	0.011	0.239	0.049	0.457
Cnuella	0.511	0.695	0.006	0.656	0.069	0.663
Euzebya	0.005	0.011	0.000	0.548	0.000	0.000
UBA4720	0.023	0.001	0.465	0.421	0.029	0.002
Isosphaera	0.796	0.003	0.018	0.032	0.177	0.000
Elioraea	0.305	0.037	0.000	0.724	0.449	1.000
Caulobacteraceae_X	0.258	0.522	0.000	0.158	0.004	0.663
GCA-2683825	0.002	0.386	0.000	0.095	0.000	0.005
Root149			0.010		0.362	0.362
Binatia_X_X_X	0.857	0.001	0.000	0.205	0.208	0.330
Anaerolineae_X_X_X	0.249	0.611	0.000	0.310	0.001	0.007
Caulobacter	0.777	0.777	0.007		0.241	0.241
Sandaracinus	0.903	0.857	0.000	1.000	0.001	0.041
URHD0088	0.279	0.033	0.000	0.600	0.634	0.689
UBA11740	0.130	0.088	0.000	0.075	0.000	0.387
BD2-11	0.118	0.177	0.000	0.914	0.001	0.112
IMCC26256	0.255	0.026	0.000	1.000	0.000	0.000
Agromyces	0.554	0.006	0.000	0.045	0.019	0.470
Singulisphaera	0.666	0.607	0.004	0.451	0.023	0.563
Mycolicibacterium	0.361	0.442	0.000	1.000	0.043	0.032

Ramlibacter	0.006		0.000	0.424	0.426	0.136
Fen-1342	0.005	0.175	0.000	0.021	0.000	0.239
UBA4093	0.499	0.855	0.502	1.000	0.833	0.834
Nitrospiraceae_X	0.571	0.029	0.000	0.239	0.005	0.637
Chloroflexales_X_X	0.676	0.006	0.000	0.205	0.471	0.000
Planctomycetota_X_X_X_X	0.093	0.009	0.000	0.008	0.000	0.216
Berkiella	0.649	0.649	0.000		0.042	0.042
Lentzea	0.684	0.029	0.000	0.158	0.005	0.635
Catenuloplanes		0.000	0.317	0.180	0.772	0.002
Domibacillus	1.000	0.114	0.499	0.265	0.731	0.213
Herminiimonas	0.559	0.442	0.000	0.424	0.042	0.127
Yuhushiella	0.559	0.559	0.090			
Gp18-AA60	0.338	0.004	0.000	0.011	0.000	0.400
W-Chloroflexi-9	0.226	0.086	0.000	0.690	0.004	0.107
Tumebacillus_A	0.338	0.003	0.772	0.295	0.469	0.005
Paracoccus	0.649	0.000	0.000	0.072	0.003	0.262
Brevundimonas	0.361	0.040	0.000	0.797	0.204	0.281
Delftia	0.777	0.777	0.080		0.414	0.414
Hungatella			0.149		0.640	0.640
Fluviicola	0.021	0.559	0.012	0.180	0.375	0.158
Bog-159	0.030	0.207	0.000	0.021	0.000	0.079
Spirillospora	0.573	0.175	0.118	0.158	0.930	0.018
Geobacterales_X_X	0.065	0.897	0.637	0.239	0.017	0.933
Ralstonia			0.019		0.414	0.414
Blastococcus_A	0.252	0.004	0.146	0.010	0.560	0.000
Acidobacteriaceae_X	0.460	0.378	0.119	0.441	0.281	0.675
UBA6092	0.828	0.480	0.000	0.548	0.030	0.001
Idiomarina			0.074		0.548	0.548
UBA7805	0.091	0.780	0.383	0.290	0.045	0.590
CSP1-2_X	0.986	0.009	0.000	0.008	0.001	0.022
Rhodococcus			0.149		0.640	0.640
HGW-BRC1-1	1.000	0.226	0.140	0.249	0.460	0.557
Caldilineales_X_X	0.391	0.542	0.031	0.441	0.064	0.563
Bacillaceae_H_X	0.096	0.951	0.933	0.180	0.085	1.000
UBA1568	0.323	0.323	0.087	1.000	0.894	0.936
UBA1268_X	0.596	0.351	0.000	0.421	0.001	0.000
Acidimicrobiaceae_X	0.255	0.980	0.043	0.424	0.071	0.386
Minicystis	0.395	0.297	0.004	0.139	0.935	0.003
Litorilinea	0.086	0.001	0.062	0.016	0.018	0.001
UTCFX2	0.650	0.001	0.000	0.094	0.127	0.000
Luteimonas	0.427	0.603	0.068	0.424	0.182	0.756
Geobacteraceae_X	0.271	0.255	0.129	0.180	0.607	0.099
Azospirillaceae_X	0.801	0.042	0.674	0.161	0.669	0.037
Sphingobium			0.000		0.060	0.060
MEBIC09517	0.000	0.002	0.000	0.180	0.009	0.190
Methylophilus	0.027	0.194	0.000	0.010	0.499	0.000
Bacillus_X			0.317		0.772	0.772
Caldilinea	0.626	0.001	0.090	0.008	0.099	0.001
Zobellella_B			0.149		0.640	0.640
Mizugakiibacter	0.598	0.621	0.000	0.607	0.002	0.087
Mucilaginibacter	0.777	0.777	0.000		0.116	0.116
Shinella			0.149		0.640	0.640

**Solitalea**			0.000		0.182	0.182
**Ferrovibrio**	0.412	0.867	0.001	0.504	0.024	0.046
**Cytophagales_X_X**	0.401	0.221	0.000	0.158	0.003	0.166
**Xanthomonadales_X_X**	0.082	0.197	0.000	0.072	0.002	0.894
**Anaerolineales_X_X**	0.800	0.092	0.000	0.290	0.031	0.605
**Massilia_B**	0.224	0.799	0.000	0.180	0.001	0.001
**A52C2**	0.164	0.016	0.000	0.032	0.044	0.001
**Flavihumibacter**	0.777	0.777	0.000		0.015	0.015
**R-RK-3**	0.501	0.008	0.883	0.161	0.545	0.009
**Altererythrobacter_A**	0.171	0.875	0.228	0.424	0.050	0.446
**Parapedobacter**			0.074		0.548	0.548
**Nitrosospira**	0.280	0.001	0.888	0.010	0.338	0.002
**Arthrobacter_F**	0.427	0.003	0.309	0.072	0.640	0.000
**Frateuria_A**	0.291	0.897	0.000	0.424	0.023	0.184
**Nocardia**	0.777	0.077	0.296	0.424	0.548	0.347
**OLB7**	0.744	0.163	0.058	0.526	0.648	0.114
**EW11**	0.603	0.097	0.000	0.797	0.446	0.745
**Actinocorallia**	0.890	0.305	0.073	0.607	0.405	0.077
**Aquamicrobium**	0.620	0.487	0.242	0.424	1.000	0.276
**Labilithrix**	1.000	0.677	0.000	0.833	0.034	0.002
**Ga0074140**	0.055	0.034	0.000	0.906	0.019	0.046
**Frankia**			0.074		0.548	0.548
**Franconibacter**			0.038		0.475	0.475
**Microbacteriaceae_X**		0.000	0.000	0.180	0.034	0.576
**Sulfuricaulis**	0.346	0.399	0.000	0.347	0.004	0.593
**Pseudoduganella**	0.941	0.157	0.001	0.480	0.233	0.628
**Beijerinckiaceae_X**	0.396	0.008	0.541	0.045	0.534	0.006
**UBA12294**	0.699	0.544	0.119	0.518	0.831	0.270
**Paceibacteria_X_X_X**	0.224	0.938	0.004	0.441	0.000	0.007
**MFUC01**	0.649	0.649	0.171			
**RBG-16-71-46_X_X_X**	0.171	0.171	0.003		0.640	0.640
**Mycobacteriaceae_X**	0.710	0.000	0.460	0.075	1.000	0.003
**Lacunisphaera**	0.024	0.559	0.043	0.180	0.459	0.241
**Glycomyces**	0.427	0.647	0.003	0.424	0.085	0.379
**Planococcaceae_X**	0.747	0.255	0.262	0.424	0.400	0.136
**UM-FILTER-40-16**	0.854	0.644	0.000	0.824	0.000	0.000
**2-12-FULL-35-15**	0.183	0.416	0.709	0.600	0.103	0.212
**Fen-1137**	0.007	0.375	0.054	0.072	0.000	0.772
**Algoriphagus**			0.149		0.640	0.640
**Chloroflexaceae_X**	0.100	0.029	0.301	0.389	0.238	0.068
**Roseomonas**	0.006		0.005	0.424	0.845	0.316
**UBA2475**	0.594	0.449	0.580	0.398	0.399	0.863
**Massilia**	0.559	0.442	0.004	0.424	0.116	0.548
**Fimbriimonadaceae_X**	0.870	0.956	0.000	1.000	0.001	0.009
**UTPRO1**	0.504	0.842	0.000	0.675	0.001	0.249
**Conexibacter_A**	0.857	0.001	0.218	0.067	0.338	0.000
**Ga0077550**	0.004	0.320	0.000	0.373	0.601	0.053
**Azoarcus_A**	0.777	0.777	0.000		0.028	0.028
**Bacillus_BE**			0.149		0.640	0.640
**Chamaesiphon**	0.427	0.561	0.026	0.424		0.007
**Paenibacillus_H**	0.559	0.559	0.231		0.362	0.362
**4572-78_X_X**	0.521	0.013	0.229	0.052	0.294	0.032

Omnitrophales_X_X	0.127	0.279	0.000	0.824	0.326	0.059
Microbacterium			0.005		0.316	0.316
Myxococcota_X_X_X_X	0.897	0.971	0.000	1.000	0.007	0.001
GCA-2683135	0.001	0.956	0.000	0.016	0.000	0.003
Labrys			0.000		0.028	0.028
GCA-002840015			0.317		0.772	0.772
Chloroflexia_X_X_X	0.759	0.053	0.000	0.421	0.001	0.000
Cellulosimicrobium	0.649	0.226	0.019	0.424	0.241	0.939
HRBIN29	0.196	0.588	0.584	0.180	0.099	0.764
Pseudomonadales_X_X	0.559	0.559	0.000		0.042	0.042
Coriobacteriales_X_X	0.398	0.171	0.000	0.045	0.000	0.284
Palsa-1382	0.777	0.777	0.000		0.042	0.042
HRBIN33	0.448	0.041	0.000	0.398	0.000	0.007
Comamonas	0.649	0.649	0.596		0.548	0.548
Acidovorax_B			0.010		0.362	0.362
UTBCD1	0.001	0.257	0.902	0.056	0.001	0.204
GJ-E10	0.866	0.940	0.002	1.000	0.152	0.267
UBA12499	0.867	0.023	0.000	0.072	0.005	0.414
Symbiobacterium	0.246	0.068	0.870	0.672	0.197	0.037
Thermoactinospora	0.559	0.559	0.090			
Cellulomonadaceae_X	0.006	0.006	0.149	1.000	0.284	0.284
Amantichitinum			0.317		0.772	0.772
Fimbriimonas	0.121	0.090	0.108	0.045	0.011	0.237
W-Firmicutes-14	0.559	0.559	0.965		0.548	0.548
Clostridiales_X_X		0.006	0.000	0.424	0.085	0.255
Stenotrophomonas_A			0.074		0.548	0.548
Cyclobacteriaceae_X	0.004	0.075	0.000	0.526	0.818	0.659
Afifella	0.082	0.137	0.137	0.072	0.116	0.068
UBA4722	0.224	1.000	0.000	0.424	0.009	0.136
Fuerstia	0.777	0.777	0.014		0.276	0.276
Azovibrio	0.777	0.777	0.080		0.414	0.414
Rubricoccus	0.218	0.898	0.053	0.236	0.026	0.315
PALSA-1448	0.255	0.255	0.021		0.640	0.640
Verrucomicrobiales_X_X	0.224	0.867	0.096	0.424	0.071	0.386
Abditibacterium	0.291	0.291	0.000		0.001	0.001
SZUA-309	0.756	0.981	0.037	1.000	0.387	0.347
Pseudonocardiaceae_X	0.565	0.028	0.777	0.205	0.393	0.020
Tistlia	0.389	0.869	0.000	0.829	0.037	0.042
HRBIN37	0.563	0.133	0.000	0.672	0.001	0.026
Ereboglobus	0.077	0.077	0.168	1.000	0.475	0.475
Nitrolancea	0.897	0.286	0.477	0.797	0.786	0.549
Phycisphaerales_X_X	0.114	0.771	0.001	0.236	0.001	0.229
Thermotalea	0.427	0.427	0.090		0.210	0.210
Bacillus_C	0.167	0.710	0.319	0.797	0.401	0.980
Roseiflexus	0.980	0.980	0.681	1.000	0.845	0.802
Marmoricola	0.769	0.055	0.233	0.181	0.936	0.004
Conexibacter	0.487	0.041	0.183	0.180	0.772	0.002
Rubrobacter	0.375	0.000	0.276	0.025	0.548	0.000
QHYU01	0.023	0.904	0.000	0.332	0.000	0.002
Mor1	0.632	0.255	0.023	0.180	0.015	0.640
UBA8087	0.621	0.752	0.006	0.607	0.475	0.048
Aquamicrobium_A		0.006	0.000	0.424	0.116	0.490

**SG8-30**	0.226	0.226	0.600	1.000	0.087	0.087
**Saccharimonadia_X_X_X**	0.278	0.978	0.000	0.441	0.055	0.023
**Micrococcaceae_X**	0.503	0.019	0.010	0.265	0.541	0.153
**Hyphomicrobium**	0.829	0.472	0.362	0.607	0.490	0.810
**GR16-43**	0.171	0.116	0.011	0.072	0.007	0.432
**Kineosporia**	0.375	0.009	0.181	0.072	0.640	0.000
**Chloroploca**	0.323	0.021	0.180	0.607	0.802	0.150
**Fen-1247**		0.006	0.074	0.424	0.548	0.347
**Patescibacteria_X_X_X_X**	0.294	0.544	0.244	0.917	0.111	0.190
**Hymenobacteraceae_X**	0.196	0.196	0.995		0.158	0.158
**Bacilli_A_X_X_X**	0.777	0.095	0.539	0.424	0.640	0.284
**Arsukibacterium**			0.149		0.640	0.640
**Magnetospirillum_A**	0.649	0.649	0.171			
**Tumebacillales_X_X**	0.559	0.559	0.694		0.475	0.475
**Vampirovibrionia_X_X_X**	0.095	0.777	0.000	0.424	0.275	0.050
**Methylocystis**	0.291	0.018	0.146	0.072	0.136	0.057
**Hydrogenedens**	0.070	0.766	0.002	0.180	0.007	0.387
**CG2-30-64-16**	0.978	0.978	0.008	1.000	0.007	0.007
**MED660**	0.205	0.252	0.000	1.000	0.247	0.214
**Vulgatibacter**	0.649	0.649	0.000		0.099	0.099
**Streptosporangiaceae_X**	0.442	0.559	0.320	0.424	0.108	0.772
**Bdellovibrio**	0.100	0.255	0.012	0.072	0.491	0.028
**Crossiella**		0.006		0.424		0.007
**Dermatophilaceae_X**	0.777	0.002	0.343	0.180		0.000
**2-12-FULL-57-22**	0.620	0.487	0.048	0.424	0.007	
**Firmicutes_E_X_X_X_X**	0.077	0.077	1.000	1.000	0.087	0.087
**Alkaliphilus**			0.038		0.475	0.475
**ABY1_X_X_X**	0.444	0.224	0.001	0.180	0.000	
**Methyloligella**	0.487	0.487	0.048			
**Blastochloris**	0.574	0.447	0.962	1.000	0.658	0.475
**Kribbella**	0.016	0.002	0.010	0.672	0.446	0.132
**Rhodomicrobiaceae_X**	0.224	0.224	0.105		0.475	0.475
**Brevibacillus**	0.777	0.777	0.284		0.548	0.548
**Pseudomonas**			0.038		0.475	0.475
**Lysinibacillus_B**	0.777	0.777	0.343			
**Bdellovibrionales_X_X**	0.839	0.171	0.646	0.424	0.980	0.210
**UBA3495_X**	0.648	0.082	0.002	0.072	0.007	0.362
**Ga0077541**	0.862	0.000	0.989	0.010	0.980	0.000
**TOLSYN**	0.026	0.082	0.001	0.007	0.000	0.548
**Hydrogenophaga**			0.000		0.034	0.034
**HZ-65**			0.002		0.276	0.276
**Crocinitomicaceae_X**	0.649	0.649	0.171			
**Bacillus_AJ**	0.000	0.000	0.019	1.000	0.112	0.087
**GCA-2725915**	0.760	0.002	0.027	0.091	0.192	0.000
**Cohnella**	0.649	0.649	0.969		0.640	0.640
**Zambryskibacteraceae_X**	0.070	0.070	0.000		0.640	0.640
**Bacteroidota_X_X_X_X**	0.255	0.255	0.000		0.009	0.009
**Enterobacterales_X_X**			0.317		0.772	0.772
**PALSA-1337**	0.059	0.375	0.014	0.072	0.000	
**SCGC-AG-212-F23**	0.559	0.442	0.001	0.424	0.116	0.361
**Bacillus_F**			0.074		0.548	0.548
**Mariniblastus**	0.224	0.444	0.205	0.180	0.362	0.125

**Pseudorhodoferax**			0.001		0.210	0.210
**JJ-A5**			0.001		0.210	0.210
**Elizabethkingia**			0.149		0.640	0.640
**Obscuribacter**	0.649	0.649	0.579		0.772	0.772
**Bacillus_AG**	0.668	0.355	0.392	0.480	0.479	0.632
**Sporomusaceae_X**	0.196	0.196	0.646	1.000	0.347	0.347
**Sphingobacteriaceae_X**	0.785	0.255	0.013	0.424	0.150	0.060
**Pajaroellobacter**	0.777	0.777	0.039		0.362	0.362
**Clostridium_AK**			0.005		0.316	0.316
**Zhizhongheella**	0.196	0.649	0.003	0.424	0.762	0.158
**Microlunatus**	0.054	0.002	0.393	0.265	0.010	0.000
**Mycobacterium_A**	0.487	0.447	0.048	0.424		0.007
**Bacillus**		0.006	0.317	0.424	0.772	0.087
**Pseudorhodoplanes**	0.649	0.649	0.011		0.210	0.210
**OLB9**	0.487	0.620	0.242	0.424	0.276	0.811
**UBA10212_X**			0.000		0.028	0.028
**Planctopirus**	0.932	0.151	0.000	0.441	0.036	0.815
**Moranbacterales_X_X**		0.006	0.000	0.424	0.050	0.133
**C7867-001**	0.777	0.777	0.539		0.640	0.640
**Bin61**	0.173	0.034	0.013	0.424	0.658	0.158
**4572-78**	0.777	0.777	1.000		0.772	0.772
**HRBIN40**	0.366	0.002	0.002	0.018	0.637	0.000
**AG11**	0.983	0.129	0.436	0.180	0.758	0.060
**Filimonas**	0.122	0.076	0.238	0.906	0.012	0.010
**Asinibacterium**	0.649	0.649	0.001		0.136	0.136
**Rummeliibacillus**	0.330	0.330	0.754		0.316	0.316
**OLB13**	1.000	0.001	0.003	0.091	0.375	0.000
**Gp7-AA6**	1.000	0.330	0.028	0.424	0.087	0.772
**Ga0074140_X**	0.224	0.224	0.015		0.640	0.640
**Desulfuromonadales_X_X**	0.000	0.649	0.358	0.072	0.003	0.475
**Exiguobacterium_A**			0.002		0.276	0.276
**Clostridium_AM**	0.777	0.077	0.076	0.424	0.414	0.777
**Ilumatobacter**	0.427	0.097	0.650	0.180	0.362	0.155
**UBA4735**	0.978	0.330	0.008	0.424	0.007	
**Elusimicrobia_X_X_X**	0.439	0.197	0.009	1.000	0.980	0.863
**Enhygromyxa**	0.463	0.345	0.005	0.797	0.236	0.980
**Feb-22**	0.777	0.777	0.343			
**Leptolyngbyaceae_X**		0.006		0.424		0.007
**Rhizobacter**			0.038		0.475	0.475
**UBA2337**	0.783	0.427	0.077	0.424	0.518	0.182
**Propionibacteriaceae_X**		0.006		0.424		0.007
**GCA-2737725**	0.559	0.559	0.004		0.158	0.158
**Paenibacillus_G**	0.649	0.649	0.171			
**Piscinibacter**	0.649	0.260	0.600	0.424	0.772	0.108
**Methylobacterium**		0.000	0.038	0.180	0.475	0.064
**GWA1-54-10**	0.978	0.330	0.604	0.424	0.863	0.210
**Solirubrobacterales_X_X**	0.179	0.215	0.082	1.000	0.010	0.012
**Nitrosomonas**	0.442	0.323	0.144	1.000	0.933	0.889
**Pseudomonas_D**			0.149		0.640	0.640
**Flavobacteriaceae_X**			0.038		0.475	0.475
**Pararhizobium**		0.006		0.424		0.007
**UBA4416**	0.566	0.534	0.289	0.724	0.157	0.157

**Palsa-465**	0.753	0.753	0.131	1.000	0.214	0.214
**SG-1**			0.317		0.772	0.772
**Lachnospiraceae_X**			0.002		0.276	0.276
**Actinomycetales_X_X**	0.260	0.196	0.600	1.000	0.108	0.087
**UBA4207**	0.513	0.171	0.158	0.424	0.213	0.071
**Fen-455**	0.649	0.649	0.171			
**Acetivibrio**	0.559	0.559	0.320		0.772	0.772
**Gp1-AA17**	0.255	0.255	0.470		0.158	0.158
**Paludisphaera**	0.330	0.016	0.012	0.072	0.085	0.252
**21-14-0-10-47-8**	0.561	0.427	0.133	0.424	0.863	0.210
**Limnobacter**	0.649	0.649	0.621		0.772	0.772
**UBA6065**	0.777	0.777	1.000		0.772	0.772
**UKL13-3**	0.109	0.427	0.794	0.180	0.073	0.475
**AG29**	0.649	0.649	0.171			
**Paenibacillus_C**			0.149		0.640	0.640
**UBA2396**	0.196	0.008	0.171	0.797	0.007	0.000
**Myxococcales_X_X**	0.829	0.224	0.292	0.424	0.826	0.414
**Sulfurifustaceae_X**	0.196	0.196	0.006		0.640	0.640
**Azotobacter**	0.777	0.777	0.343			
**UBA9983_A_X_X**	0.149	0.149	0.057		0.362	0.362
**UBA4124**	0.559	0.559	0.090			
**Asticcacaulis**			0.000		0.182	0.182
**Rickettsiaceae_X**	0.777	0.777	0.308		0.548	0.548
**Paenibacillus_E**	0.777	0.777	0.559		0.640	0.640
**Klebsiella_B**		0.006	0.149	0.424	0.640	0.284
**Vitreoscilla_A**		0.006	0.038	0.424	0.475	0.518
**Paracraurococcus**	0.799	0.000	0.054	0.010	0.518	0.000
**20-14-0-80-50-8**	0.178	0.070	0.000	0.424	0.007	
**SZUA-46**	0.777	0.777	0.343			
**Diaphorobacter**			0.149		0.640	0.640
**Caulobacterales_X_X**	0.649	0.649	0.393		0.475	0.475
**Mongoliimonas**			0.074		0.548	0.548
**Gimesia**	0.839	0.171	0.003	0.424	0.214	0.640
**Streptomyces**	0.196	0.196	0.564	1.000	0.347	0.387
**Nitrospira_D**	0.649	0.226	0.070	0.424	0.316	0.845
**Palsa-1315**	0.649	0.649	0.171			
**Dyella**			0.149		0.640	0.640
**Nannocystaceae_X**	0.111	0.934	0.073	0.180	0.276	0.318
**Palsa-1392**	0.649	0.260	0.026	0.424	0.241	0.818
**Jidaibacter**			0.005		0.316	0.316
**Bog-1198**	0.559	0.040	0.094	0.180	0.276	0.428
**Promicromonospora**	0.777	0.777	0.539		0.640	0.640
**Rhodothermales_X_X**	0.149	0.775	0.056	0.424	0.034	0.344
**Enterococcaceae_X**			0.149		0.640	0.640
**UBA10190**	0.850	0.330	0.082	0.424	0.214	0.640
**GCA-002862325**	0.255	0.338	0.364	0.180	0.362	0.112
**Pseudobacteroides**	0.006			0.424	0.007	
**Porphyrobacter**	0.559	0.323	0.043	0.424	0.241	0.939
**Fulvimonas**		0.006	0.010	0.424	0.362	0.883
**Peptostreptococcales_X_X**			0.010		0.362	0.362
**Bacteriovoracaceae_X**	0.777	0.002	0.003	0.180	0.210	0.684
**GCA-2770975**	0.905	0.224	0.004	0.424	0.230	0.042

**Bin94**	0.291	0.291	0.004			
**Rubritepida**	0.487	0.574	0.003	0.424	0.085	0.534
**Obscuribacteraceae_X**	0.378	0.070	0.001	0.072	0.004	0.414
**Paracaedibacter**			0.149		0.640	0.640
**Elainella**	0.427	0.076	0.026	0.180		0.000
**Geminicoccus**	0.649	0.196	0.238	0.424	0.414	0.593
**SG8-24_X**	0.649	0.649	0.171			
**Paenibacillus**			0.019		0.414	0.414
**A4b_X**	0.472	0.938	0.442	0.607	0.190	0.702
**UBA1268**	0.649	0.649	0.171			
**UBA12115**	0.980	0.255	0.002	0.424	0.007	
**GCA-002050365**	0.777	0.777	0.007		0.241	0.241
**Ga0077555**	0.938	0.291	0.040	0.424	0.214	0.640
**2013-40CM-41-45**	0.561	0.097	0.151	0.607	0.980	0.459
**ZC4RG24**	0.487	0.487	0.015		0.158	0.158
**Parachlamydiales_X_X**	0.330	0.330	0.610		0.414	0.414
**GCA-2699025**	0.196	0.196	0.903		0.210	0.210
**Clostridium_T**			0.074		0.548	0.548
**Moheibacter**			0.010		0.362	0.362
**Cronobacter**			0.317		0.772	0.772
**Gorillibacterium**		0.006	0.038	0.424	0.475	0.563
**Pelomonas**	0.777	0.777	0.161		0.475	0.475
**Hydrocarboniphaga**	0.777	0.777	0.073		0.414	0.414
**Gemmatimonas**	0.559	0.323	0.048	0.424	0.241	1.000
**Bacillus_BC**	0.916	0.396	0.541	0.656	0.918	0.299
**UBA1018_X_X**	0.224	0.649	0.012	0.424	0.640	0.247
**Spirosomaceae_X**	0.649	0.649	0.009		0.210	0.210
**Tatlockia**	0.323	0.559	0.320	0.424	0.087	0.772
**Pigmentiphaga**			0.149		0.640	0.640
**Micavibrio_B**	0.196	0.330	0.042	0.180	0.879	0.116
**GAS474**			0.149		0.640	0.640
**Hyphomicrobiaceae_X**	0.427	0.737	0.725	0.424	0.475	0.518
**Enterococcus**	0.006			0.424	0.007	
**Saccharopolyspora**	0.649	0.649	0.171			
**Tol-SR**	0.095	0.777	0.308	0.424	0.347	0.548
**Anaerobacillus**	0.196	0.557	0.113	0.180	0.414	0.141
**Weeksellaceae_X**			0.074		0.548	0.548
**Chryseomicrobium**			0.019		0.414	0.414
**Niabella**			0.074		0.548	0.548
**Bdellovibrionota_X_X_X_X**	0.777	0.777	1.000		0.772	0.772
**O2-12-FULL-42-9**	0.559	0.559	0.090			
**HO2-45-11b**	0.777	0.777	0.343			
**XYD1-FULL-53-11**	0.649	0.012	0.969	0.180	0.640	0.012
**GWC2-37-73_X**	0.006		0.317	0.424	0.108	0.772
**Angustibacter**			0.317		0.772	0.772
**Babeliales_X_X**	0.980	0.255	0.142	0.424	0.609	0.116
**Actinoplanes**		0.000	0.038	0.180	0.475	0.123
**Amycolatopsis**	0.649	0.260	0.171	0.424		0.007
**2-01-FULL-59-12**	0.912	0.060	0.518	0.106	0.849	0.104
**Nostocaceae_X**	0.886	0.668	0.169	1.000	0.284	0.214
**F0540**			0.038		0.475	0.475
**Desulfonispora**	0.574	0.487	0.007	0.424	0.375	0.136

**2-12-FULL-44-17**	0.487	0.487	0.048			
**SZUA-149**	0.070	0.252	0.075	0.424	0.182	0.792
**Bacillus_H**			0.317		0.772	0.772
**UBA10450_X**	0.487	0.447	0.583	0.424	0.414	0.729
**UBA920**	0.777	0.777	0.049		0.362	0.362
**Chitinibacteraceae_X**			0.317		0.772	0.772
**Xiphinematobacter**	0.559	0.442	1.000	0.424	0.548	0.475
**MPNL01**	0.559	0.559	0.650		0.475	0.475
**Sumerlaeaceae_X**		0.000		0.180		0.000
**Acidicaldus**		0.006		0.424		0.007
**1-14-0-10-56-10**	0.777	0.777	0.559		0.640	0.640
**PMMR1**			0.001		0.241	0.241
**Desulfitobacteriaceae_X**			0.317		0.772	0.772
**Herpetosiphonaceae_X**	0.777	0.077	0.037	0.424	0.362	0.791
**Mesorhizobium_A**			0.317		0.772	0.772
**Cesiribacter**			0.149		0.640	0.640
**OLB11**			0.038		0.475	0.475
**Uliginosibacterium**	0.777	0.777	0.045		0.362	0.362
**Propionibacteriales_X_X**	0.777	0.077	0.343	0.424		0.007
**Streptosporangium**	0.777	0.077	0.971	0.424	0.772	0.087
**Parachlamydiaceae_X**	0.777	0.077	0.539	0.424	0.640	0.214
**2-01-FULL-44-21**	0.427	0.427	0.026			
**SZUA-47**	0.777	0.777	0.343			
**UBA922_X**	0.777	0.777	0.343			
**Sporichthya**	0.649	0.260	0.131	0.424	0.362	0.791
**Thermanaerothrix**	0.330	0.330	0.030		0.772	0.772
**Peribacteraceae_X**	0.291	0.291	0.040		0.640	0.640
**Ellin6529_X_X_X**	0.487	0.620	0.048	0.424		0.007
**UBA6164_X**	0.777	0.777	0.343			
**AKYH767_X_X**	0.077	0.777	0.343	0.424	0.007	
**Taibaiella_B**	0.561	0.783	0.753	1.000	0.475	0.610
**32-67-11**			0.317		0.772	0.772
**Enterobacter_B**			0.074		0.548	0.548
**Paeniclostridium**	0.777	0.777	0.343			
**UBA6821**			0.149		0.640	0.640
**Mycolicibacillus**	0.649	0.649	0.171			
**Legionellaceae_X**	0.777	0.095	1.000	0.424	0.772	0.087
**Methyloceanibacter**	0.649	0.226	0.171	0.424		0.007
**2-02-FULL-42-43**	0.850	0.330	0.085	0.424	0.214	0.640
**2-02-FULL-39-32**	0.649	0.649	0.171			
**Koribacter**	0.777	0.777	1.000		0.772	0.772
**Kapabacteriales_X_X**	0.224	0.224	0.565		0.276	0.276
**Rickettsiella**			0.317		0.772	0.772
**Pseudescherichia**			0.074		0.548	0.548
**UBA9655**	0.487	0.487	0.766		0.414	0.414
**Geobacter_B**			0.149		0.640	0.640
**UBA9649**	0.330	0.330	0.082		0.640	0.640
**Lysinibacillus**	0.323	0.559	0.027	0.424	0.863	0.210
**Ga0074137**	0.753	0.167	0.469	0.607	0.936	0.318
**UBA9934**	0.649	0.649	0.663		0.548	0.548
**Melittangium**	0.559	0.559	0.090			
**Rudanella**	0.777	0.002	0.284	0.180	0.548	0.029

**Solimonas**	0.777	0.777	0.343			
**Comamonas_E**			0.317		0.772	0.772
**Microtrichales_X_X**	0.559	0.400	0.090	0.424		0.007
**Sporomusa**		0.006	0.317	0.424	0.772	0.087
**Risungbinella**	0.361	0.034	0.698	0.607	0.247	0.018
**PALSA-555**	0.777	0.777	0.343			
**Eubacterium_F**			0.317		0.772	0.772
**UBA11600**	0.559	0.559	0.665		0.475	0.475
**Acidisphaera**	0.095	0.777	0.559	0.424	0.214	0.640
**UBA9628**	0.892	0.196	0.020	0.180	0.057	0.548
**DDH964**	0.777	0.777	1.000		0.772	0.772
**Stackebrandtia**		0.006		0.424		0.007
**Methylacidiphilales_X_X**	0.444	0.255	0.009	0.180	0.003	0.772
**UBA11359_X**	0.777	0.777	0.091		0.414	0.414
**Brevibacillus_B**	0.777	0.777	0.343			
**UBA12053**	0.777	0.777	1.000		0.772	0.772
**Oligoflexus**	0.559	0.323	1.000	0.424	0.548	0.347
**Phreatobacter**			0.010		0.362	0.362
**SG8-24_X_X**	0.003	0.777	0.343	0.180	0.000	
**2-02-FULL-39-32_X_X**	0.559	0.559	0.090			
**Altererythrobacter_B**	0.777	0.777	0.154		0.475	0.475
**Turicibacter**			0.317		0.772	0.772
**Actinotalea**			0.317		0.772	0.772
**UBA11704**			0.149		0.640	0.640
**Thermoanaerobaculia_X_X_X**	0.323	0.559	1.000	0.424	0.347	0.548
**SZUA-55**	0.167	0.375	0.444	0.180	0.048	0.475
**QKMZ01**	0.649	0.649	0.990		0.640	0.640
**Nucleicultrix**			0.317		0.772	0.772
**Sphaerospermopsis_A**	0.777	0.095	0.343	0.424		0.007
**Faecalicatena**	0.777	0.777	0.343			
**Polyangia_X_X_X**	0.777	0.777	0.168		0.475	0.475
**Bacillus_L**			0.149		0.640	0.640
**OLB15**	0.649	0.649	0.001		0.136	0.136
**01-FULL-45-10b**	0.559	0.559	0.983		0.548	0.548
**UBA1931**			0.019		0.414	0.414
**GCA-2699585**	0.649	0.000	0.034	0.072	0.276	0.057
**UBA1547**			0.074		0.548	0.548
**2-12-FULL-60-19**	0.006			0.424	0.007	
**Aquimonas**			0.317		0.772	0.772
**Bacillus_AX**			0.149		0.640	0.640
**Khelaifiella**			0.317		0.772	0.772
**OLB5**	0.856	0.291	0.016	0.424	0.108	0.772
**OLB20_X**	0.487	0.487	0.694		0.548	0.548
**OLB23**	0.649	0.649	0.171			
**GCF-001939115**	0.777	0.077	0.343	0.424		0.007
**Paenibacillus_T**			0.317		0.772	0.772
**Vermiphilus**			0.019		0.414	0.414
**Nevskiales_X_X**	0.649	0.649	0.171			
**HR-BB**	0.649	0.226	0.171	0.424		0.007
**Cyanobacteria_X_X_X_X**	0.427	0.137	0.255	0.180	0.640	0.012
**Tumebacillus**	0.559	0.559	0.090			
**HdN1**	0.559	0.559	0.983		0.548	0.548

**2-02-FULL-49-12**			0.317		0.772	0.772
**Bacillus_Q**			0.317		0.772	0.772
**Sphingomonas**			0.317		0.772	0.772
**BOG-1112**	0.559	0.559	0.107		0.276	0.276
**UBA9973_X**	0.777	0.777	0.343			
**SZUA-521**	0.487	0.487	0.709		0.548	0.548
**UBA9631**	0.427	0.737	0.100	0.424	0.772	0.108
**Algiphilus**			0.019		0.414	0.414
**Gastranaerophilales_X_X**			0.038		0.475	0.475
**UBA1844**	0.649	0.649	0.621		0.772	0.772
**Larkinella**	0.777	0.077	0.076	0.424	0.414	0.637
**Bdellovibrionaceae_X**	0.006		0.317	0.424	0.087	0.772
**UBA4658**	0.447	0.487	0.947	1.000	0.475	0.475
**PALSA-1347**			0.317		0.772	0.772
**Deinococcaceae_X**	0.777	0.777	0.343			
**Serratia**			0.317		0.772	0.772
**Aquincola**			0.317		0.772	0.772
**Alsobacter**		0.000	0.149	0.072	0.640	0.000
**Chondromyces**	0.559	0.442	0.360	0.424	0.772	0.108
**UTPRO2**	0.649	0.649	0.600		0.772	0.772
**Bacillus_AT**	0.559	0.559	0.090			
**UBA11063**			0.149		0.640	0.640
**Ancylobacter**			0.317		0.772	0.772
**GCA-2707205**			0.149		0.640	0.640
**Deinococcus_C**			0.317		0.772	0.772
**SZUA-359**			0.317		0.772	0.772
**RBG-16-40-8**	0.487	0.487	0.183		0.772	0.772
**Deinococcales_X_X**	0.777	0.777	0.343			
**Paracaedibacteraceae_X**	0.649	0.649	0.358		0.475	0.475
**Thermoactinomycetaceae_X**	0.649	0.649	0.990		0.640	0.640
**Zoogloea**			0.149		0.640	0.640
**Paenibacillus_N**			0.317		0.772	0.772
**Oscillatoria**	0.095	0.777	0.343	0.424	0.007	
**Segetibacter**	0.649	0.649	0.171			
**Flavobacteriales_X_X**	0.559	0.559	0.414		0.414	0.414
**Ochrobactrum_A**			0.317		0.772	0.772
**Tistrella**	0.649	0.649	0.171			
**BAL199**	0.649	0.649	0.171			
**UBA1400_X_X**	0.777	0.777	0.343			
**UBA1565_X**	0.427	0.427	0.026			
**UBA920_X_X**	0.649	0.649	0.579		0.772	0.772
**Shewanella**			0.317		0.772	0.772
**Chelatococcus**			0.317		0.772	0.772
**Pedobacter**	0.777	0.777	0.519		0.640	0.640
**UBA1565_X_X**	0.777	0.777	1.000		0.772	0.772
**UBA1532**	0.559	0.559	0.090			
**XYD2-FULL-39-9**	0.777	0.777	0.343			
**UBA10212**	0.777	0.777	0.343			
**Legionella_A**			0.038		0.475	0.475
**UBA11426**	0.777	0.777	0.343			
**Aquabacterium**			0.149		0.640	0.640
**WO2-54-10**	0.777	0.777	0.343			

**Terribacillus**	0.649	0.649	0.596		0.548	0.548
**Dehalobacterium**	0.559	0.559	0.090			
**Vogesella**			0.317		0.772	0.772
**UBA1573**			0.149		0.640	0.640
**UBA6139**	0.649	0.649	0.171			
**UBA1369_X_X**			0.149		0.640	0.640
**Dietzia**			0.317		0.772	0.772
**Bacteroidales_X_X**			0.149		0.640	0.640
**Thiobacillus**			0.317		0.772	0.772
**Amphiplicatus**		0.006		0.424		0.007
**Desulfovibrionales_X_X**		0.006	0.317	0.424	0.772	0.087
**Amphibacillaceae_X**		0.006		0.424		0.007
**Brevibacillaceae_X**		0.006		0.424		0.007
**Paenibacillus_L**		0.000	0.074	0.180	0.548	0.029
**GWA2-52-8**	0.095	0.777	0.343	0.424	0.007	
**UBA10190_X**	0.442	0.559	0.090	0.424	0.007	
**Elusimicrobiota_X_X_X_X**	0.777	0.777	0.343			
**SZUA-592**	0.777	0.777	1.000		0.772	0.772
**Micavibrio**	0.649	0.649	0.579		0.772	0.772
**Odyssella**			0.317		0.772	0.772
**01-FULL-45-34b**			0.317		0.772	0.772
**Rubinisphaera**	0.777	0.777	0.971		0.772	0.772
**Simkania**			0.317		0.772	0.772
**Gracilibacteria_X_X_X**			0.317		0.772	0.772
**Sphingomonas_D**			0.317		0.772	0.772
**DSM-45891**			0.317		0.772	0.772
**Gracilibacillus**	0.649	0.649	0.621		0.772	0.772
**Siphonobacter**			0.074		0.548	0.548
**Caloramatoraceae_X**			0.149		0.640	0.640
**1NLA3E**	0.777	0.777	0.343			
**Longispora**			0.317		0.772	0.772
**Opitutales_X_X**			0.317		0.772	0.772
**Salinimicrobium**	0.777	0.077	0.343	0.424		0.007
**Leptolyngbya_A**	0.226	0.226	0.600	1.000	0.087	0.087
**Xanthomonas_A**			0.317		0.772	0.772
**Deinococcus**			0.149		0.640	0.640
**VKM-B-2647**	0.777	0.777	0.971		0.772	0.772
**21-14-0-10-47-8-A**			0.038		0.475	0.475
**Bdellovibrio_A**			0.317		0.772	0.772
**Ruminococcus_A**			0.149		0.640	0.640
**Desulfotomaculum_H**	0.777	0.777	0.148		0.475	0.475
**UBA2163_X**	0.777	0.777	0.087		0.414	0.414
**Ktedonobacter**	0.006			0.424	0.007	
**2-02-FULL-51-10**	0.559	0.559	0.090			
**OLB19**			0.038		0.475	0.475
**28-YEA-48**			0.317		0.772	0.772
**Palsa-1150**			0.317		0.772	0.772
**Enterococcus_A**			0.317		0.772	0.772
**OLB8**			0.149		0.640	0.640
**Kroppenstedtia**		0.006	0.149	0.424	0.640	0.247
**Ketobacteraceae_X**		0.006	0.317	0.424	0.772	0.108
**Elainellaceae_X**	0.777	0.777	0.343			

**Anaerocolumna**			0.317		0.772	0.772
**GCA-2686445**			0.149		0.640	0.640
**Bacteriovorax**			0.149		0.640	0.640
**Solirubrum**			0.149		0.640	0.640
**HO2-37-13b_X**	0.777	0.777	0.971		0.772	0.772
**Protochlamydia**			0.149		0.640	0.640
**Desulfobacterota_X_X_X_X**	0.777	0.777	0.559		0.640	0.640
**Diplorickettsia**	0.649	0.649	0.171			
**Magnetospirillaceae_X**	0.649	0.649	0.171			
**PALSA-968**			0.149		0.640	0.640
**GWA1-44-29**	0.649	0.649	0.171			
**UBA10103**	0.649	0.649	0.171			

Table S4: p-values from pairwise adonis between all collections of rhizosphere samples. The grey shaded boxes represent collections from same crop developmental stage.

	Collection-2	Collection-3	Collection-4	Collection-5	Collection-6	Collection-7	Collection-8
Collection-1	0.003	0.012	0.012	0.009	0.013	0.009	0.006
Collection-2		0.057	0.009	0.013	0.013	0.01	0.015
Collection-3			0.013	0.004	0.005	0.006	0.006
Collection-4				0.215	0.011	0.018	0.012
Collection-5					0.039	0.028	0.009
Collection-6						0.557	0.013
Collection-7							0.008

Table S5: p-values from Phylum abundance comparison of rhizosphere samples across crop development stages using Kruskal-Wallis test. BH = Benjamini Hochberg. The comparisons are made with and without R1 group.

	**Rhizosphere samples**		**Rhizosphere samples (except R1)**	
**Phyla**	p-value	BH adjusted p-value	p-value	BH adjusted p-value
**Proteobacteria**	0.00123	0.00230	0.07163	0.08000
**Firmicutes**	0.00002	0.00013	0.00018	0.00090
**Planctomycetota**	0.00043	0.00110	0.01768	0.02500
**Acidobacteriota**	0.00014	0.00051	0.00251	0.00610
**Verrucomicrobiota**	0.00017	0.00056	0.00514	0.01000
**Actinobacteriota**	0.00010	0.00046	0.00006	0.00038
**Bacteroidota**	0.00252	0.00400	0.15079	0.16000
**Unclassified Phylum**	0.02618	0.03000	0.04105	0.05400
**Firmicutes_A**	0.00011	0.00046	0.00220	0.00610
**Patescibacteria**	0.00000	0.00009	0.00003	0.00020
**Gemmatimonadota**	0.02247	0.02700	0.06004	0.07200
**Chloroflexota**	0.00161	0.00280	0.00256	0.00610
**Myxococcota**	0.00432	0.00590	0.01015	0.01700
**Methylomirabilota**	0.08080	0.08400	0.39593	0.40000
**Binatota**	0.00047	0.00110	0.00263	0.00610
**Chloroflexota_A**	0.00001	0.00009	0.00002	0.00020
**Firmicutes_I**	0.00057	0.00110	0.00071	0.00270
**Nitrospirota**	0.02021	0.02500	0.00566	0.01100
**Eisenbacteria**	0.03323	0.03600	0.04916	0.06100
**Armatimonadota**	0.00240	0.00400	0.16852	0.17000
**Bdellovibrionota**	0.00411	0.00590	0.01310	0.02000
**Sumerlaeota**	0.00001	0.00009	0.00002	0.00020
**Verrucomicrobiota_A**	0.00051	0.00110	0.00727	0.01300
**Firmicutes_K**	0.00654	0.00850	0.00394	0.00840
**Cyanobacteria**	0.02812	0.03100	0.02793	0.03800
**Desulfuromonadota**	0.00035	0.00094	0.00089	0.00300
**Dadabacteria**	0.11305	0.11000	0.06649	0.07700
**Hydrogenedentota**	0.00419	0.00590	0.01271	0.02000
**Omnitrophota**	0.00002	0.00012	0.00001	0.00020
**Elusimicrobiota**	0.00025	0.00074	0.00044	0.00190

Table S6: p-values from Genus abundance comparison of rhizosphere samples across crop development stages using Kruskal-Wallis test. BH = Benjamini Hochberg. The comparisons are made with and without R1 group.

	Rhizosphere samples		Rhizosphere samples (except R1)	
Genus	p-value	BH adjusted p-value	p-value	BH adjusted p-value
**Pseudomonas_M**	0.00004	0.00062	0.00073	0.00540
**Acinetobacter**	0.01698	0.03600	0.08592	0.15000
**Enterobacter_D**	0.06747	0.11000	0.10919	0.18000
**Bacillus_W**	0.00008	0.00097	0.00051	0.00450
**Bacillus_AK**	0.00001	0.00031	0.00006	0.00170
**UBA2421**	0.00128	0.00520	0.07523	0.14000
**Bacillaceae_A_X**	0.00020	0.00160	0.00342	0.01400
**Enterobacteriaceae_X**	0.00670	0.01800	0.00279	0.01300
**Pseudomonadaceae_X**	0.00014	0.00130	0.00081	0.00580
**Sphingomonadaceae_X**	0.00003	0.00060	0.00027	0.00320
**Lysobacter**	0.02530	0.05000	0.02267	0.05600
**Bacteria_X_X_X_X_X**	0.02618	0.05200	0.04105	0.08800
**Luteitalea**	0.00032	0.00210	0.01199	0.03500
**Rhizobiaceae_X**	0.00080	0.00380	0.04073	0.08800
**Bacillales_X_X**	0.00014	0.00130	0.00028	0.00330
**OLB17**	0.00026	0.00190	0.00720	0.02400
**Peptostreptococcaceae_X**	0.00009	0.00110	0.00181	0.00990
**QHWT01**	0.00051	0.00270	0.00849	0.02700
**Chthoniobacter**	0.00032	0.00210	0.01220	0.03500
**Flavobacterium**	0.00184	0.00680	0.00298	0.01300
**Haloferula**	0.00170	0.00640	0.10821	0.18000
**Pseudosphingobacterium**	0.00037	0.00230	0.00039	0.00380
**Xylophilus**	0.00004	0.00064	0.00003	0.00099
**Sphningosinicella**	0.00005	0.00067	0.00043	0.00410
**Xanthomonadaceae_X**	0.00014	0.00130	0.00089	0.00600
**Verrucomicrobium**	0.00647	0.01700	0.55587	0.62000
**Chryseobacterium**	0.01300	0.02900	0.11021	0.18000
**Bacillus_Y**	0.00011	0.00120	0.00125	0.00750
**Alphaproteobacteria_X_X_X**	0.00003	0.00060	0.00013	0.00200
**Burkholderiaceae_X**	0.00031	0.00210	0.00070	0.00530
**Saccharimonadaceae_X**	0.00004	0.00064	0.00021	0.00280
**Sphingopyxis_A**	0.00272	0.00920	0.00878	0.02800
**Pontibacter**	0.00010	0.00110	0.00024	0.00300
**GCA-2746885**	0.00001	0.00031	0.00008	0.00170
**Fimbriiglobus**	0.00013	0.00130	0.00196	0.01000
**Clostridiaceae_X**	0.00090	0.00410	0.00947	0.02900
**Acidimicrobiia_X_X_X**	0.00203	0.00730	0.00809	0.02600
**Planctomycetaceae_X**	0.00006	0.00078	0.00049	0.00440
**Neorhizobium**	0.00082	0.00380	0.00857	0.02700
**Pseudoxanthomonas_A**	0.25065	0.33000	0.73479	0.77000
**Clostridium**	0.00098	0.00440	0.01744	0.04500
**Sphingomonas_A**	0.00008	0.00097	0.00082	0.00580
**QHXM01**	0.01064	0.02500	0.00595	0.02100
**UKL13-2**	0.00000	0.00024	0.00001	0.00062
**Pirellulales_X_X**	0.00030	0.00210	0.00960	0.02900
**Mycoplana**	0.02031	0.04200	0.18103	0.27000
**Flavisolibacter**	0.00158	0.00610	0.04345	0.09200

**Leaf454**	0.00007	0.00086	0.00084	0.00580
**Sphingobacterium**	0.42680	0.50000	0.35992	0.46000
**Gemmata**	0.00055	0.00290	0.00096	0.00630
**Clostridium_AD**	0.00202	0.00730	0.00662	0.02300
**Alteromonadaceae_X**	0.00000	0.00006	NA	NA
**Steroidobacter**	0.00019	0.00160	0.00298	0.01300
**Pirellulaceae_X**	0.00203	0.00730	0.11488	0.19000
**Microvirga**	0.00008	0.00097	0.00038	0.00380
**Pedosphaeraceae_X**	0.00000	0.00024	0.00001	0.00062
**UBA1161_X**	0.00011	0.00120	0.00303	0.01300
**Pirellula**	0.00117	0.00490	0.03018	0.06900
**Lysobacter_A**	0.10411	0.16000	0.84049	0.87000
**Gemmatimonadaceae_X**	0.00390	0.01200	0.01019	0.03000
**UBA4664**	0.00000	0.00024	0.00001	0.00062
**Cellvibrio**	0.07179	0.12000	0.58967	0.65000
**Gp6-AA56**	0.00517	0.01500	0.01702	0.04500
**Pedosphaera**	0.00001	0.00031	0.00001	0.00062
**Chitinophagaceae_X**	0.00040	0.00230	0.00909	0.02800
**Rhodocyclaceae_X**	0.00004	0.00060	0.00006	0.00170
**Stenotrophomonas**	0.49462	0.55000	0.37927	0.47000
**Bacilli_X_X_X**	0.00029	0.00200	0.00209	0.01100
**Actinobacteriota_X_X_X_X**	0.00459	0.01300	0.00153	0.00880
**Palsa-739**	0.00028	0.00190	0.00037	0.00380
**Bacillus_BD**	0.00003	0.00060	0.00008	0.00170
**Pedosphaerales_X_X**	0.00001	0.00031	0.00007	0.00170
**Micromonosporaceae_X**	0.00004	0.00062	0.00012	0.00200
**Burkholderiales_X_X**	0.00021	0.00170	0.00061	0.00500
**Ohtaekwangia**	0.00011	0.00120	0.00281	0.01300
**Kosakonia**	0.47176	0.53000	0.52575	0.60000
**Cupriavidus**	0.60486	0.65000	0.65812	0.71000
**UBA4665_X**	0.00581	0.01600	0.49543	0.58000
**GWC2-73-18**	0.00001	0.00031	0.00002	0.00094
**UBA9968_X**	0.00041	0.00240	0.00252	0.01200
**Streptomycetaceae_X**	0.05109	0.09000	0.02943	0.06800
**UBA11741**	0.02010	0.04100	0.01805	0.04700
**Gemmataceae_X**	0.00013	0.00130	0.00108	0.00690
**UBA6082**	0.00002	0.00047	0.00002	0.00094
**GWA2-73-35_X**	0.07795	0.13000	0.09104	0.16000
**Chitinophaga**	0.17823	0.24000	0.49279	0.58000
**Polyangiales_X_X**	0.00031	0.00210	0.00034	0.00350
**Phenylobacterium**	0.06722	0.11000	0.21108	0.30000
**Bacillus_AA**	0.00072	0.00350	0.00473	0.01800
**Methylibium**	0.00051	0.00270	0.00352	0.01400
**Pararheinheimera**	0.00000	0.00024	0.51252	0.58000
**BOG-1338**	0.00000	0.00024	0.00001	0.00085
**Adhaeribacter**	0.01232	0.02800	0.28323	0.38000
**Exiguobacterium**	0.00154	0.00600	0.00188	0.01000
**Methylophilaceae_X**	0.00429	0.01300	0.00330	0.01400
**AR5**	0.01011	0.02400	0.02800	0.06500
**Gp7-AA10**	0.00119	0.00500	0.00197	0.01000
**Planobispora**	0.00037	0.00230	0.00019	0.00260
**Rhizobium**	0.00425	0.01300	0.00815	0.02600

**Erwinia**	0.49197	0.55000	0.67882	0.73000
**JKG1**	0.00252	0.00870	0.00272	0.01300
**Bryobacteraceae_X**	0.00016	0.00140	0.00008	0.00170
**Dongia**	0.00175	0.00660	0.03319	0.07400
**CP2B**	0.12506	0.18000	0.07174	0.13000
**Achromobacter**	0.99524	1.00000	0.97549	0.98000
**Pyrinomonadaceae_X**	0.00053	0.00280	0.00083	0.00580
**Flavobacterium_A**	0.08219	0.13000	0.05472	0.11000
**Vicinamibacterales_X_X**	0.00349	0.01100	0.19728	0.28000
**Geminicoccaceae_X**	0.01219	0.02800	0.00528	0.01900
**Pseudoxanthomonas**	0.41638	0.49000	0.29413	0.39000
**2-12-FULL-64-23**	0.00003	0.00060	0.00003	0.00099
**Paenibacillaceae_X**	0.00086	0.00390	0.00400	0.01600
**SZUA-115**	0.00070	0.00350	0.00026	0.00320
**Gemmatirosa**	0.01289	0.02900	0.02589	0.06100
**Inquilinus**	0.00430	0.01300	0.00896	0.02800
**Vitiosangium**	0.01092	0.02600	0.00707	0.02400
**Paramesorhizobium**	0.03308	0.06200	0.04284	0.09100
**Aeromonas**	0.00238	0.00830	0.00579	0.02000
**Gammaproteobacteria_X_X_X**	0.00014	0.00130	0.00029	0.00330
**Terrimicrobium**	0.00851	0.02200	0.35115	0.45000
**Arthrobacter_I**	0.00348	0.01100	0.00198	0.01000
**FW-11**	0.01492	0.03300	0.00901	0.02800
**Acidimicrobiales_X_X**	0.01485	0.03300	0.05663	0.11000
**Geodermatophilus**	0.00122	0.00500	0.00071	0.00540
**Kouleothrix**	0.04937	0.08800	0.06509	0.12000
**Saprospiraceae_X**	0.00013	0.00130	0.00113	0.00700
**Palsa-1233**	0.00001	0.00031	0.00001	0.00062
**ARS98**	0.00341	0.01100	0.00397	0.01600
**Rhizobiales_X_X**	0.00003	0.00060	0.00003	0.00110
**Zavarzinella**	0.00000	0.00024	0.00001	0.00062
**UBA5946**	0.00590	0.01600	0.20359	0.29000
**Bordetella_A**	0.00049	0.00270	0.00123	0.00750
**Bradyrhizobium**	0.00006	0.00085	0.00011	0.00200
**Planctomycetes_X_X_X**	0.00396	0.01200	0.00812	0.02600
**Fictibacillus_B**	0.00148	0.00590	0.00399	0.01600
**QHVH01**	0.01477	0.03300	0.04376	0.09200
**Citrobacter**	0.44090	0.51000	0.37275	0.47000
**Chthoniobacterales_X_X**	0.00044	0.00250	0.01841	0.04700
**Chitinophagales_X_X**	0.00497	0.01400	0.00293	0.01300
**UBA10511**	0.00099	0.00440	0.03189	0.07200
**Burkholderia**	0.09485	0.15000	0.27921	0.38000
**Thermomicrobiaceae_X**	0.01786	0.03800	0.09357	0.16000
**Catelliglobosispora**	0.00442	0.01300	0.05223	0.10000
**Alishewanella**	0.00000	0.00024	0.51252	0.58000
**Arboricoccus**	0.00004	0.00062	0.00006	0.00170
**Novosphingobium**	0.10310	0.16000	0.09184	0.16000
**Opitutaceae_X**	0.00003	0.00060	0.00004	0.00130
**Dyadobacter**	0.06207	0.11000	0.25745	0.35000
**UBA1020**	0.00218	0.00760	0.00980	0.02900
**MM2**	0.12106	0.18000	0.16562	0.25000
**Saccharimonadales_X_X**	0.00110	0.00470	0.01210	0.03500

**Domibacillaceae_X**	0.00198	0.00720	0.00243	0.01200
**Ilumatobacter_A**	0.00096	0.00430	0.00556	0.02000
**Aeromicrobium**	0.00025	0.00190	0.00085	0.00580
**Saccharibacillus**	0.59183	0.64000	0.51252	0.58000
**Rubrobacter_B**	0.00040	0.00230	0.00216	0.01100
**Gemmatimonadales_X_X**	0.01604	0.03500	0.00903	0.02800
**Blastomonas**	0.11504	0.17000	0.20507	0.29000
**Pirellula_B**	0.00003	0.00060	0.00005	0.00160
**Plantactinospora_A**	0.01007	0.02400	0.00892	0.02800
**Nitrospira_A**	0.00821	0.02100	0.01136	0.03300
**PMNU01**	0.00632	0.01700	0.02056	0.05100
**PALSA-1355**	0.15875	0.22000	0.26390	0.36000
**Myxococcus**	0.01577	0.03400	0.07624	0.14000
**Acetobacteraceae_X**	0.00056	0.00290	0.01632	0.04300
**Reyranella**	0.00022	0.00170	0.00055	0.00470
**Arenimonas**	0.00025	0.00190	0.00024	0.00300
**Isosphaeraceae_X**	0.00792	0.02100	0.02659	0.06300
**Rhodobacteraceae_X**	0.01076	0.02600	0.00480	0.01800
**Pseudomonas_A**	0.00488	0.01400	0.01972	0.05000
**Taibaiella_A**	0.40981	0.49000	0.90105	0.92000
**Sphingopyxis**	0.03213	0.06100	0.01744	0.04500
**Verrucomicrobiae_X_X_X**	0.00025	0.00190	0.00136	0.00800
**Rhizorhabdus**	0.63676	0.68000	0.52951	0.60000
**UBA2386**	0.00003	0.00060	0.00015	0.00220
**Devosia**	0.02770	0.05400	0.01940	0.04900
**Aromatoleum**	0.00058	0.00300	0.00135	0.00800
**Chryseolinea_A**	0.00003	0.00060	0.00006	0.00170
**Prosthecobacter**	0.00334	0.01100	0.15507	0.24000
**Mycobacteriales_X_X**	0.04231	0.07700	0.02376	0.05800
**Pseudomonas_F**	0.00097	0.00440	0.01408	0.03900
**Thauera**	0.00012	0.00130	0.00012	0.00200
**AR19**	0.00957	0.02300	0.01301	0.03600
**Opitutus**	0.00001	0.00031	0.00001	0.00062
**Rubellimicrobium**	0.06249	0.11000	0.10845	0.18000
**Actinobacteria_X_X_X**	0.00374	0.01200	0.00191	0.01000
**Nevskiaceae_X**	0.00014	0.00130	0.00099	0.00640
**SCN-70-22**	0.49677	0.55000	0.49443	0.58000
**Bosea**	0.02917	0.05600	0.61713	0.67000
**Solirubrobacteraceae_X**	0.00994	0.02400	0.01316	0.03700
**Steroidobacteraceae_X**	0.00019	0.00160	0.00248	0.01200
**Herpetosiphon**	0.01769	0.03700	0.03774	0.08200
**Solirubrobacter**	0.02432	0.04900	0.01188	0.03500
**Rhodanobacteraceae_X**	0.00003	0.00060	0.00012	0.00200
**Noviherbaspirillum**	0.01943	0.04000	0.01253	0.03600
**Bin18**	0.00483	0.01400	0.01743	0.04500
**Niastella**	0.07539	0.12000	0.14674	0.23000
**NS-102**	0.33786	0.42000	0.88803	0.91000
**Pseudomonas_H**	0.00033	0.00210	NA	NA
**Flavisolibacter_B**	0.10034	0.15000	0.46412	0.55000
**Pseudonocardia**	0.01286	0.02900	0.00755	0.02500
**SCN-69-37**	0.00168	0.00640	0.01245	0.03600
**Palsa-89**	0.00185	0.00680	0.03284	0.07400

**Blastocatellia_X_X_X**	0.10054	0.15000	0.08042	0.14000
**Proteobacteria_X_X_X_X**	0.01481	0.03300	0.01484	0.04000
**UBA4720**	0.00111	0.00470	0.00360	0.01500
**Bacteroidia_X_X_X**	0.00741	0.02000	0.06012	0.12000
**Azohydromonas**	0.01161	0.02700	0.26433	0.36000
**Nocardioidaceae_X**	0.05884	0.10000	0.03005	0.06900
**Caulobacteraceae_X**	0.06953	0.12000	0.10297	0.17000
**Nitrospira_C**	0.08562	0.13000	0.08484	0.15000
**Root149**	0.11882	0.18000	0.06223	0.12000
**Planctomyces_A**	0.00540	0.01500	0.12163	0.20000
**Jiangella**	0.00050	0.00270	0.00033	0.00350
**Isoptericola_B**	0.00137	0.00550	0.03643	0.08000
**Xanthobacteraceae_X**	0.02674	0.05200	0.04531	0.09400
**SM1A02_X**	0.00003	0.00060	0.00015	0.00220
**ZC4RG30**	0.01140	0.02700	0.06633	0.12000
**Blastococcus**	0.03364	0.06300	0.05609	0.11000
**Agromyces**	0.07840	0.13000	0.69366	0.74000
**BOG-1460**	0.00064	0.00320	0.00045	0.00420
**Phycisphaerae_X_X_X**	0.02851	0.05500	0.35656	0.45000
**SWB02**	0.04978	0.08800	0.30522	0.40000
**Mycolicibacterium**	0.07255	0.12000	0.49231	0.58000
**Ramlibacter**	0.03473	0.06400	0.07266	0.13000
**IMCC26207**	0.03566	0.06600	0.02784	0.06500
**Aneurinibacillus_A**	0.00256	0.00880	0.00176	0.00970
**Dactylosporangium**	0.02619	0.05200	0.36211	0.46000
**P52-10**	0.00003	0.00060	0.00013	0.00210
**Thermoleophilia_X_X_X**	0.00753	0.02000	0.00528	0.01900
**Berkiella**	0.00005	0.00067	0.00014	0.00210
**Domibacillus**	0.57932	0.63000	0.60304	0.66000
**Herminiimonas**	0.02520	0.05000	0.04775	0.09800
**Bin134**	0.00071	0.00350	0.00067	0.00530
**Paracoccus**	0.22298	0.30000	0.19454	0.28000
**UBA11883**	0.00055	0.00290	0.00159	0.00910
**Delftia**	0.31964	0.40000	0.33461	0.43000
**Hungatella**	0.10310	0.16000	0.09184	0.16000
**Chloroflexota_X_X_X_X**	0.14402	0.21000	0.51298	0.58000
**Ralstonia**	0.00000	0.00006	NA	NA
**Anaeromyxobacter**	0.00085	0.00390	0.00090	0.00600
**URHD0088**	0.00001	0.00031	0.00003	0.00099
**Rubrobacteraceae_X**	0.01214	0.02800	0.01059	0.03200
**Polymorphum**	0.00468	0.01400	0.06574	0.12000
**SZUA-252**	0.00952	0.02300	0.01974	0.05000
**Lentzea**	0.00008	0.00097	0.00009	0.00180
**Streptacidiphilus**	0.00099	0.00440	0.40038	0.49000
**Idiomarina**	0.00033	0.00210	NA	NA
**Rhodococcus**	0.21850	0.29000	0.19155	0.28000
**HGW-BRC1-1**	0.00001	0.00031	0.00002	0.00099
**KBS-96**	0.00027	0.00190	0.00058	0.00500
**Azospirillum**	0.16074	0.22000	0.15747	0.24000
**Acidimicrobiaceae_X**	0.59066	0.64000	0.60568	0.66000
**AV2**	0.00002	0.00058	0.00007	0.00170
**Litorilinea**	0.00108	0.00470	0.00094	0.00620

**Nonomuraea**	0.00446	0.01300	0.00227	0.01100
**Brevundimonas**	0.05530	0.09600	0.03788	0.08200
**Luteimonas**	0.33298	0.41000	0.23310	0.32000
**Nocardioides**	0.00270	0.00920	0.00585	0.02100
**Tumebacillus_A**	0.00284	0.00940	0.00200	0.01000
**Gemmatimonadetes_X_X_X**	0.01950	0.04000	0.01332	0.03700
**Sphingobium**	0.02712	0.05300	0.03523	0.07800
**Bacillus_X**	0.59183	0.64000	0.51252	0.58000
**Zobellella_B**	0.00885	0.02200	NA	NA
**Terrimonas**	0.00280	0.00940	0.00660	0.02300
**Spirillospora**	0.75549	0.77000	0.60147	0.66000
**Shinella**	0.21850	0.29000	0.19155	0.28000
**Solitalea**	0.03540	0.06600	0.06003	0.12000
**Ferrovibrio**	0.00058	0.00300	0.00035	0.00360
**Cytophagales_X_X**	0.00655	0.01800	0.03026	0.06900
**Xanthomonadales_X_X**	0.00502	0.01400	0.10029	0.17000
**RBG-16-71-46**	0.01618	0.03500	0.01421	0.03900
**A52C2**	0.00152	0.00600	0.00304	0.01300
**Flavihumibacter**	0.00878	0.02200	0.02797	0.06500
**Parapedobacter**	0.06464	0.11000	0.06181	0.12000
**Nitrosospira**	0.00123	0.00500	0.00066	0.00530
**Polyangiaceae_X**	0.00011	0.00120	0.00009	0.00180
**Frateuria_A**	0.02342	0.04700	0.18035	0.27000
**Acidobacteriales_X_X**	0.00135	0.00540	0.00120	0.00740
**OLB7**	0.00051	0.00270	0.00175	0.00970
**Schlesneria**	0.04276	0.07800	0.04452	0.09300
**Actinocorallia**	0.00037	0.00230	0.00031	0.00340
**Caldilinea**	0.00365	0.01100	0.01121	0.03300
**Myxococcaceae_X**	0.04850	0.08600	0.03845	0.08300
**Ga0074140**	0.00062	0.00320	0.00938	0.02900
**Frankia**	0.00033	0.00210	0.00047	0.00420
**Franconibacter**	0.57911	0.63000	0.58283	0.65000
**Byssovorax**	0.47790	0.54000	0.32074	0.42000
**Microbacteriaceae_X**	0.02066	0.04200	0.09379	0.16000
**Mucilaginibacter**	0.02822	0.05400	0.01885	0.04800
**QHBO01**	0.00085	0.00390	0.00074	0.00540
**Bacillaceae_H_X**	0.13731	0.20000	0.37628	0.47000
**Mycobacteriaceae_X**	0.07250	0.12000	0.08986	0.16000
**Nannocystis**	0.08015	0.13000	0.05089	0.10000
**Lacunisphaera**	0.00484	0.01400	0.00676	0.02300
**Cnuella**	0.12257	0.18000	0.09181	0.16000
**Glycomyces**	0.00820	0.02100	0.01430	0.03900
**Planococcaceae_X**	0.00162	0.00620	0.00352	0.01400
**Acidobacteriota_X_X_X_X**	0.64910	0.69000	0.44943	0.54000
**Algoriphagus**	0.00885	0.02200	NA	NA
**Geodermatophilaceae_X**	0.01414	0.03100	0.00793	0.02600
**Roseomonas**	0.08578	0.13000	0.10085	0.17000
**UBA2475**	0.00013	0.00130	0.00021	0.00280
**Oscillochloris**	0.46749	0.53000	0.83177	0.86000
**Massilia**	0.46785	0.53000	0.33486	0.43000
**Dehalococcoidia_X_X_X**	0.02969	0.05700	0.01796	0.04600
**55-13**	0.00077	0.00370	0.01993	0.05000

**Azoarcus_A**	0.00111	0.00470	0.00066	0.00530
**Pseudoduganella**	0.09675	0.15000	0.04356	0.09200
**Bacillus_BE**	0.00885	0.02200	NA	NA
**Paenibacillus_H**	0.45427	0.52000	0.50261	0.58000
**Microbacterium**	0.40169	0.48000	0.40685	0.50000
**Labrys**	0.00887	0.02200	0.02994	0.06900
**GCA-002840015**	0.59183	0.64000	0.51252	0.58000
**Cellulosimicrobium**	0.72871	0.75000	0.95503	0.96000
**40CM-3-62-11**	0.01223	0.02800	0.01651	0.04400
**HRBIN29**	0.23306	0.31000	0.17934	0.27000
**Pseudomonadales_X_X**	0.21163	0.29000	0.17849	0.27000
**Palsa-1382**	0.00277	0.00940	0.01299	0.03600
**Comamonas**	0.05413	0.09400	0.51252	0.58000
**Acidovorax_B**	0.87625	0.88000	0.98639	0.99000
**GJ-E10**	0.00008	0.00097	0.00022	0.00280
**Sandaracinus**	0.00360	0.01100	0.00217	0.01100
**Cellulomonadaceae_X**	0.67825	0.72000	0.62725	0.68000
**Amantichitinum**	0.15860	0.22000	0.13278	0.21000
**Hyalangium**	0.38754	0.47000	0.41069	0.50000
**Fimbriimonas**	0.00685	0.01800	0.04264	0.09100
**Clostridiales_X_X**	0.01132	0.02700	0.03373	0.07500
**Stenotrophomonas_A**	0.88048	0.89000	0.89177	0.91000
**Cyclobacteriaceae_X**	0.00003	0.00060	0.00011	0.00200
**Azospirillaceae_X**	0.26505	0.34000	0.16890	0.26000
**UBA4655**	0.16810	0.23000	0.09704	0.16000
**R-RK-3**	0.00790	0.02100	0.00438	0.01700
**UBA4722**	0.01353	0.03000	0.21727	0.30000
**Anaerolineae_X_X_X**	0.01230	0.02800	0.07543	0.14000
**Azovibrio**	0.00020	0.00160	0.00033	0.00350
**Nocardia**	0.47533	0.54000	0.44715	0.54000
**Blastococcus_A**	0.65042	0.69000	0.48017	0.57000
**Verrucomicrobiales_X_X**	0.38654	0.47000	0.48335	0.57000
**Abditibacterium**	0.00016	0.00140	0.00402	0.01600
**Nitrolancea**	0.02631	0.05200	0.04453	0.09300
**Isosphaera**	0.00432	0.01300	0.00476	0.01800
**Aquamicrobium_A**	0.05655	0.09800	0.05469	0.11000
**Saccharimonadia_X_X_X**	0.00026	0.00190	0.00170	0.00960
**Chryseolinea**	0.03640	0.06700	0.08485	0.15000
**Rubricoccus**	0.05380	0.09400	0.06158	0.12000
**Sorangium**	0.89712	0.90000	0.78395	0.82000
**Par-f-2**	0.01228	0.02800	0.00605	0.02100
**Arthrobacter_F**	0.44793	0.52000	0.51252	0.58000
**Chloroflexia_X_X_X**	0.14376	0.21000	0.08864	0.15000
**Chthonomonas**	0.00548	0.01500	0.00273	0.01300
**Bacilli_A_X_X_X**	0.44793	0.52000	0.51252	0.58000
**Rhodospirillum_A**	0.16059	0.22000	0.07941	0.14000
**Arsukibacterium**	0.00885	0.02200	NA	NA
**UBA6092**	0.01718	0.03600	0.02847	0.06600
**UBA7805**	0.06791	0.11000	0.12036	0.19000
**Vampirovibrionia_X_X_X**	0.00077	0.00370	0.00310	0.01300
**Geobacteraceae_X**	0.00120	0.00500	0.00274	0.01300
**Hydrogenedens**	0.00419	0.01300	0.01271	0.03600

**Vulgatibacter**	0.01665	0.03600	0.02081	0.05100
**Altererythrobacter_A**	0.03258	0.06100	0.04968	0.10000
**Bdellovibrio**	0.07644	0.12000	0.07094	0.13000
**QKVK01**	0.00025	0.00190	0.00148	0.00860
**Chloroflexales_X_X**	0.07322	0.12000	0.14416	0.23000
**CSP1-2_X**	0.16800	0.23000	0.10395	0.17000
**Planctomicrobium**	0.06160	0.10000	0.05532	0.11000
**Massilia_B**	0.00511	0.01400	0.00758	0.02500
**Alkaliphilus**	0.74600	0.77000	0.77361	0.81000
**Thermotalea**	0.27307	0.35000	0.39992	0.49000
**UTCFX2**	0.08293	0.13000	0.11473	0.19000
**Brevibacillus**	0.30151	0.38000	0.27897	0.38000
**Pseudomonas**	0.39357	0.47000	0.38381	0.48000
**Binatia_X_X_X**	0.33938	0.42000	0.22685	0.32000
**W-Chloroflexi-9**	0.09365	0.15000	0.13548	0.21000
**TOLSYN**	0.32508	0.40000	0.30471	0.40000
**Micrococcaceae_X**	0.27415	0.35000	0.17939	0.27000
**Hydrogenophaga**	0.53042	0.59000	0.96262	0.97000
**Labilithrix**	0.01863	0.03900	0.02751	0.06500
**UBA12294**	0.02072	0.04200	0.01352	0.03700
**HZ-65**	0.00046	0.00260	0.00068	0.00530
**G233**	0.04676	0.08400	0.02427	0.05800
**MEBIC09517**	0.16913	0.23000	0.16280	0.25000
**Aquamicrobium**	0.38872	0.47000	0.48005	0.57000
**Tumebacillales_X_X**	0.22757	0.30000	0.61596	0.67000
**Bacillus_C**	0.01516	0.03300	0.02022	0.05000
**GCA-2723275**	0.03173	0.06000	0.32493	0.42000
**Bacteroidota_X_X_X_X**	0.00017	0.00140	0.00042	0.00410
**Enterobacterales_X_X**	0.15860	0.22000	NA	NA
**SCGC-AG-212-F23**	0.00625	0.01700	0.01574	0.04200
**UBA4093**	0.25836	0.33000	0.18719	0.28000
**Bacillus_F**	0.49581	0.55000	0.47056	0.56000
**Minicystis**	0.00179	0.00670	0.00212	0.01100
**UTBCD1**	0.00323	0.01100	0.00334	0.01400
**Pseudorhodoferax**	0.27802	0.36000	0.40740	0.50000
**Kineosporia**	0.21850	0.29000	0.19155	0.28000
**Azoarcus_B**	0.00565	0.01600	0.00336	0.01400
**Patescibacteria_X_X_X_X**	0.00034	0.00210	0.00113	0.00700
**UBA1268_X**	0.12753	0.19000	0.17897	0.27000
**JJ-A5**	0.91850	0.92000	0.96514	0.97000
**Caldilineales_X_X**	0.72764	0.75000	0.74962	0.79000
**UBA6960_X**	0.00319	0.01000	0.00251	0.01200
**Elizabethkingia**	0.41877	0.49000	0.36907	0.46000
**Sphingobacteriaceae_X**	0.13396	0.20000	0.14799	0.23000
**Pseudonocardiaceae_X**	0.05997	0.10000	0.07037	0.13000
**LHW63021**	0.11695	0.17000	0.08807	0.15000
**Pajaroellobacter**	0.00046	0.00260	0.00067	0.00530
**Clostridium_AK**	0.27880	0.36000	0.33574	0.43000
**GR16-43**	0.00017	0.00150	0.00009	0.00180
**Elioraea**	0.11461	0.17000	0.07087	0.13000
**GWC2-70-10**	0.05660	0.09800	0.40135	0.49000
**Zhizhongheella**	0.00038	0.00230	0.00076	0.00550

**HRBIN30**	0.04526	0.08200	0.23293	0.32000
**Cryptosporangium**	0.04347	0.07900	0.08291	0.15000
**Methylocystis**	0.00152	0.00600	0.00096	0.00630
**UBA10212_X**	0.00105	0.00460	0.00664	0.02300
**Moranbacterales_X_X**	0.01368	0.03000	0.05241	0.10000
**Planctomycetota_X_X_X_X**	0.00014	0.00130	0.00030	0.00330
**C7867-001**	0.67825	0.72000	0.62725	0.68000
**EW11**	0.00207	0.00740	0.00504	0.01900
**Planctopirus**	0.01905	0.04000	0.10261	0.17000
**Symbiobacterium**	0.07488	0.12000	0.05877	0.11000
**Asinibacterium**	0.02285	0.04600	0.04526	0.09400
**Rummeliibacillus**	0.73037	0.76000	0.57388	0.64000
**Caulobacter**	0.68465	0.72000	0.91652	0.93000
**AG11**	0.77286	0.79000	0.75501	0.79000
**Exiguobacterium_A**	0.02705	0.05300	0.03402	0.07500
**Clostridium_AM**	0.18362	0.25000	0.19200	0.28000
**Z2-YC6860**	0.00391	0.01200	0.01423	0.03900
**Rhizobacter**	0.87140	0.88000	0.91027	0.93000
**UBA2337**	0.00006	0.00085	0.00011	0.00200
**Cystobacter**	0.39990	0.48000	0.26871	0.37000
**GCA-2737725**	0.10739	0.16000	0.18804	0.28000
**Hymenobacteraceae_X**	0.08693	0.14000	0.04479	0.09400
**Methylobacterium**	0.10643	0.16000	0.10791	0.18000
**UBA5793**	0.72874	0.75000	0.70938	0.75000
**Nitrosomonas**	0.00078	0.00370	0.06181	0.12000
**Pseudomonas_D**	0.00885	0.02200	NA	NA
**Flavobacteriaceae_X**	0.89008	0.90000	0.93557	0.95000
**SZUA-42**	0.27088	0.35000	0.92730	0.94000
**Ilumatobacter**	0.55557	0.61000	0.38736	0.48000
**UBA4416**	0.01165	0.02700	0.01252	0.03600
**SG-1**	0.59183	0.64000	0.51252	0.58000
**Lachnospiraceae_X**	0.13785	0.20000	0.08590	0.15000
**UBA4207**	0.29479	0.37000	0.23156	0.32000
**Paludisphaera**	0.01900	0.04000	0.04929	0.10000
**Bacillus_AG**	0.04772	0.08500	0.02562	0.06100
**21-14-0-10-47-8**	0.60179	0.65000	0.85333	0.88000
**Limnobacter**	0.15860	0.22000	NA	NA
**Kribbella**	0.08404	0.13000	0.11440	0.19000
**NIC37A-2**	0.11058	0.17000	0.06327	0.12000
**GCA-002686595_X**	0.07679	0.12000	0.10587	0.18000
**Paenibacillus_C**	0.21850	0.29000	0.19155	0.28000
**Hyphomicrobium**	0.01604	0.03500	0.03351	0.07500
**Beijerinckiaceae_X**	0.16671	0.23000	0.11760	0.19000
**Myxococcales_X_X**	0.02131	0.04300	0.02511	0.06000
**40CM-68-15**	0.06587	0.11000	0.03637	0.08000
**Singulisphaera**	0.35234	0.43000	0.67130	0.72000
**Asticcacaulis**	0.33225	0.41000	0.52435	0.59000
**Klebsiella_B**	0.35833	0.44000	0.31433	0.41000
**Vitreoscilla_A**	0.00001	0.00031	0.00002	0.00094
**2-12-FULL-35-15**	0.19631	0.27000	0.31097	0.41000
**Diaphorobacter**	0.00885	0.02200	NA	NA
**Mongoliimonas**	0.45326	0.52000	0.42187	0.51000

**SZUA-318**	0.10874	0.16000	0.27122	0.37000
**Dyella**	0.35833	0.44000	0.31433	0.41000
**Afifella**	0.38660	0.47000	0.24714	0.34000
**Piscinibacter**	0.15860	0.22000	NA	NA
**Jidaibacter**	0.00526	0.01500	0.00679	0.02300
**Promicromonospora**	0.67825	0.72000	0.62725	0.68000
**Rhodothermales_X_X**	0.50578	0.56000	0.70297	0.75000
**Enterococcaceae_X**	0.67825	0.72000	0.62725	0.68000
**Porphyrobacter**	0.98774	0.99000	0.95981	0.97000
**Anaerolineales_X_X**	0.22808	0.30000	0.30467	0.40000
**Fulvimonas**	0.70085	0.73000	0.78303	0.82000
**Rhodomicrobiaceae_X**	0.52558	0.58000	0.52308	0.59000
**Methylophilus**	0.15549	0.22000	0.08073	0.14000
**Peptostreptococcales_X_X**	0.32189	0.40000	0.36206	0.46000
**UBA1568**	0.33804	0.42000	0.42397	0.51000
**Bacteriovoracaceae_X**	0.48379	0.55000	0.68568	0.73000
**GCA-2770975**	0.00070	0.00350	0.00128	0.00760
**Desulfuromonadales_X_X**	0.42662	0.50000	0.42323	0.51000
**Rubritepida**	0.00296	0.00970	0.00391	0.01600
**Paracaedibacter**	0.10310	0.16000	0.09184	0.16000
**Palsa-1392**	0.40118	0.48000	0.55402	0.62000
**Paenibacillus**	0.69730	0.73000	0.75272	0.79000
**Rubrobacter**	0.45326	0.52000	0.42187	0.51000
**Fimbriimonadaceae_X**	0.05846	0.10000	0.05227	0.10000
**2013-40CM-41-45**	0.59071	0.64000	0.80782	0.84000
**Euzebya**	0.02642	0.05200	0.02389	0.05800
**ZC4RG24**	0.52063	0.58000	0.85377	0.88000
**Clostridium_T**	0.42657	0.50000	0.39908	0.49000
**Ga0077550**	0.00283	0.00940	0.00197	0.01000
**Moheibacter**	0.18516	0.25000	0.20586	0.29000
**Omnitrophales_X_X**	0.00115	0.00480	0.00061	0.00500
**Gorillibacterium**	0.73787	0.76000	0.76319	0.80000
**67-14**	0.36423	0.44000	0.23918	0.33000
**Geobacterales_X_X**	0.00209	0.00740	0.00283	0.01300
**Fluviicola**	0.04661	0.08400	0.07750	0.14000
**Pelomonas**	0.56736	0.62000	0.56776	0.63000
**Hydrocarboniphaga**	0.00489	0.01400	0.00567	0.02000
**Gemmatimonas**	0.03217	0.06100	0.04631	0.09500
**Bacillus_BC**	0.17694	0.24000	0.11922	0.19000
**Spirosomaceae_X**	0.16162	0.22000	0.12269	0.20000
**Pigmentiphaga**	0.21850	0.29000	0.19155	0.28000
**Micavibrio_B**	0.01077	0.02600	0.02268	0.05600
**UKL13-3**	0.00294	0.00970	0.00340	0.01400
**GAS474**	0.35833	0.44000	0.31433	0.41000
**Weeksellaceae_X**	0.30151	0.38000	0.19155	0.28000
**Coleofasciculus**	0.07520	0.12000	0.19155	0.28000
**IMCC26256**	0.47186	0.53000	0.45860	0.55000
**SZUA-320**	0.14251	0.21000	0.13224	0.21000
**Chryseomicrobium**	0.06035	0.10000	0.06181	0.12000
**Niabella**	0.42657	0.50000	0.39908	0.49000
**UBA12499**	0.15505	0.22000	0.16054	0.25000
**BD2-11**	0.53131	0.59000	0.36138	0.46000

**Babeliales_X_X**	0.00182	0.00680	0.00440	0.01700
**Actinoplanes**	0.41581	0.49000	0.27897	0.38000
**Ga0077541**	0.01370	0.03000	0.00578	0.02000
**4572-78_X_X**	0.71287	0.74000	0.51333	0.58000
**Acidobacteriaceae_X**	0.70110	0.73000	0.97674	0.98000
**F0540**	0.71453	0.74000	0.73274	0.77000
**2-01-FULL-59-12**	0.00761	0.02000	0.00650	0.02200
**Desulfonispora**	0.15423	0.22000	0.31149	0.41000
**UBA10450_X**	0.13554	0.20000	0.13581	0.21000
**Bog-1198**	0.05942	0.10000	0.07675	0.14000
**Nostocaceae_X**	0.21850	0.29000	0.19155	0.28000
**Bacillus_AJ**	0.06566	0.11000	0.06181	0.12000
**PMMR1**	0.08407	0.13000	0.11182	0.18000
**Chloroflexaceae_X**	0.07607	0.12000	0.04994	0.10000
**Pseudorhodoplanes**	0.00842	0.02200	0.00560	0.02000
**UTPRO1**	0.02418	0.04800	0.14826	0.23000
**Herpetosiphonaceae_X**	0.03813	0.07000	0.04556	0.09400
**Cesiribacter**	0.21850	0.29000	0.19155	0.28000
**OLB11**	0.00001	0.00031	0.00002	0.00094
**Uliginosibacterium**	0.19355	0.26000	0.20684	0.29000
**Chloroploca**	0.74861	0.77000	0.58277	0.65000
**Sporichthya**	0.29302	0.37000	0.33282	0.43000
**GCA-2699025**	0.11800	0.18000	0.17153	0.26000
**Paracraurococcus**	0.09078	0.14000	0.19155	0.28000
**Nitrospiraceae_X**	0.22975	0.30000	0.13592	0.21000
**Gp1-AA17**	0.82893	0.84000	0.83676	0.86000
**Enterobacter_B**	0.32508	0.40000	0.30471	0.40000
**Bin61**	0.15499	0.22000	0.09644	0.16000
**UBA11740**	0.03287	0.06200	0.00909	0.02800
**Coriobacteriales_X_X**	0.41877	0.49000	0.51252	0.58000
**Hyphomicrobiaceae_X**	0.08686	0.14000	0.08756	0.15000
**Roseiflexus**	0.50763	0.56000	0.60344	0.66000
**Pseudescherichia**	0.64685	0.69000	0.62454	0.68000
**Caulobacterales_X_X**	0.56736	0.62000	0.56776	0.63000
**Bog-159**	0.38984	0.47000	0.33004	0.42000
**Lysinibacillus**	0.15827	0.22000	0.24176	0.33000
**HRBIN40**	0.66008	0.70000	0.51010	0.58000
**GCA-2725915**	0.07931	0.13000	0.09587	0.16000
**Blastochloris**	0.25399	0.33000	0.71877	0.76000
**Elusimicrobia_X_X_X**	0.00385	0.01200	0.00557	0.02000
**GCA-002050365**	0.68917	0.72000	0.56781	0.63000
**Paenibacillus_E**	0.21850	0.29000	0.19155	0.28000
**Nitrospira_D**	0.28178	0.36000	0.31593	0.41000
**Phreatobacter**	0.44202	0.51000	0.49993	0.58000
**Haliangium**	0.41699	0.49000	0.28045	0.38000
**Parachlamydiales_X_X**	0.51742	0.57000	0.53694	0.61000
**Fen-1247**	0.49581	0.55000	0.47056	0.56000
**Gp18-AA60**	0.25151	0.33000	0.16039	0.25000
**Altererythrobacter_B**	0.28055	0.36000	0.27254	0.37000
**Tol-SR**	0.01835	0.03900	0.01880	0.04800
**Taibaiella_B**	0.39357	0.47000	0.25589	0.35000
**Marmoricola**	0.55527	0.61000	0.57241	0.64000

**GWC2-71-9_X**	0.79691	0.81000	0.68896	0.74000
**Fen-1342**	0.33457	0.41000	0.25182	0.35000
**OLB15**	0.00196	0.00720	0.00427	0.01700
**01-FULL-45-10b**	0.00033	0.00210	0.00047	0.00420
**UBA1931**	0.01368	0.03000	0.01634	0.04300
**UBA8087**	0.43835	0.51000	0.30471	0.40000
**GCA-2699585**	0.02367	0.04800	0.03101	0.07000
**UBA1547**	0.63428	0.68000	0.60908	0.67000
**Conexibacter_A**	0.31637	0.40000	0.21416	0.30000
**SZUA-149**	0.38298	0.46000	0.30606	0.40000
**UBA920**	0.86486	0.88000	0.73405	0.77000
**UBA3495_X**	0.15812	0.22000	0.14297	0.22000
**Vermiphilus**	0.42486	0.50000	0.44243	0.53000
**Nannocystaceae_X**	0.11167	0.17000	0.13145	0.21000
**Bdellovibrionales_X_X**	0.24162	0.32000	0.37619	0.47000
**OLB9**	0.05104	0.09000	0.06218	0.12000
**HRBIN37**	0.27870	0.36000	0.17256	0.26000
**Sulfuricaulis**	0.34662	0.43000	0.59295	0.66000
**GWA1-54-10**	0.03332	0.06200	0.05299	0.11000
**Phycisphaerales_X_X**	0.84574	0.86000	0.76384	0.80000
**Sporomusaceae_X**	0.44037	0.51000	0.41447	0.50000
**Algiphilus**	0.00939	0.02300	0.01090	0.03200
**Gastranaerophilales_X_X**	0.05154	0.09000	0.05136	0.10000
**Ga0074137**	0.46674	0.53000	0.59881	0.66000
**Cyanobacteriales_X_X**	0.76175	0.78000	0.68275	0.73000
**Larkinella**	0.84446	0.86000	0.92068	0.93000
**Kapabacteriales_X_X**	0.00968	0.02400	0.00242	0.01200
**BOG-1112**	0.68822	0.72000	0.82917	0.86000
**Tistlia**	0.56635	0.62000	0.44844	0.54000
**UBA9983_A_X_X**	0.00216	0.00760	0.00319	0.01400
**Polyangia_X_X_X**	0.20295	0.27000	0.20180	0.29000
**Fuerstia**	0.14342	0.21000	0.17965	0.27000
**Myxococcota_X_X_X_X**	0.29851	0.38000	0.32880	0.42000
**GCA-2683135**	0.04539	0.08200	0.02366	0.05800
**A4b_X**	0.04632	0.08300	0.02379	0.05800
**UBA9655**	0.20022	0.27000	0.21407	0.30000
**Flavobacteriales_X_X**	0.23603	0.31000	0.24407	0.34000
**GCA-2683825**	0.00712	0.01900	0.00211	0.01100
**Geminicoccus**	0.00361	0.01100	0.00457	0.01700
**OLB13**	0.10364	0.16000	0.18531	0.28000
**Cyanobacteriia_X_X_X**	0.25145	0.33000	0.28724	0.38000
**Enhygromyxa**	0.13637	0.20000	0.06572	0.12000
**UBA11359_X**	0.04976	0.08800	0.05450	0.11000
**HRBIN33**	0.75078	0.77000	0.85017	0.88000
